# Synthesis of molecular metallic barium superhydride: pseudocubic BaH_12_

**DOI:** 10.1038/s41467-020-20103-5

**Published:** 2021-01-11

**Authors:** Wuhao Chen, Dmitrii V. Semenok, Alexander G. Kvashnin, Xiaoli Huang, Ivan A. Kruglov, Michele Galasso, Hao Song, Defang Duan, Alexander F. Goncharov, Vitali B. Prakapenka, Artem R. Oganov, Tian Cui

**Affiliations:** 1 State Key Laboratory of Superhard Materials, College of Physics, Jilin University, Changchun, 130012 China; 2 Skolkovo Institute of Science and Technology, Skolkovo Innovation Center, 3 Nobel Street, Moscow, 143026 Russia; 3 Moscow Institute of Physics and Technology, 9 Institutsky Lane, Dolgoprudny, 141700 Russia; 4 Dukhov Research Institute of Automatics (VNIIA), Moscow, 127055 Russia; 5 Earth and Planets Laboratory, Carnegie Institution of Washington, 5251 Broad Branch Road NW, Washington, DC 20015 USA; 6 Center for Advanced Radiation Sources, The University of Chicago, 5640 South Ellis Avenue, Chicago, IL 60637 USA; 7 School of Physical Science and Technology, Ningbo University, Ningbo, 315211 China

**Keywords:** Electronic properties and materials, Structure of solids and liquids, Superconducting properties and materials, Computational science

## Abstract

Following the discovery of high-temperature superconductivity in the La–H system, we studied the formation of new chemical compounds in the barium-hydrogen system at pressures from 75 to 173 GPa. Using in situ generation of hydrogen from NH_3_BH_3_, we synthesized previously unknown superhydride BaH_12_ with a pseudocubic (*fcc*) Ba sublattice in four independent experiments. Density functional theory calculations indicate close agreement between the theoretical and experimental equations of state. In addition, we identified previously known *P*6*/mmm*-BaH_2_ and possibly BaH_10_ and BaH_6_ as impurities in the samples. Ab initio calculations show that newly discovered semimetallic BaH_12_ contains H_2_ and H_3_^–^ molecular units and detached H_12_ chains which are formed as a result of a Peierls-type distortion of the cubic cage structure. Barium dodecahydride is a unique molecular hydride with metallic conductivity that demonstrates the superconducting transition around 20 K at 140 GPa.

## Abstract (web summary)

Metallization of pure hydrogen via overlapping of electronic bands requires high pressure above 3 Mbar. Here the authors study the Ba-H system and discover a unique superhydride BaH_12_ that contains molecular hydrogen, which demonstrates metallic properties and superconductivity below 1.5 Mbar.

## Introduction

In recent years, the search for new hydride superconductors with *T*_C_ close to room temperature attracts great attention of researchers in the field of high-pressure materials science. Variation of pressure opens prospects of synthesis of novel functional materials with unexpected properties^1^. For example, according to theoretical models^2–5^, compression of molecular hydrogen over 500 GPa should lead to the formation of an atomic metallic modification with *T*_C_ near room temperature. Pressures of 420–480 GPa were achieved in experiments with toroidal diamond anvil cells^6^; however, for conventional high-pressure cells with a four-electrode electric setup, pressures above 200 GPa remain challenging.

In 2004, Ashcroft^7^ suggested an alternative method of searching for high-*T*_C_ superconductors that uses other elements, metals or nonmetals, to precompress the hydrogen atoms, which should lead to a dramatic decrease in the metallization pressure. A decade later this idea found its experimental proof. Extraordinarily high superconducting transition temperatures were demonstrated in compressed $Im\bar 3m$-H_3_S^8–11^ (203 K at 150 GPa), $Im\bar 3m$-YH_6_^12^ and *P*6_3_*/mmc*-YH_9_^13^ (224 K at 166 GPa and 243 K at 237 GPa, respectively), $Fm\bar 3m$-ThH_10_^14^ (161 K at 174 GPa), *P*6_3_*/mmc*-CeH_9_ (~100 K)^15^, and lanthanum decahydride $Fm\bar 3m$-LaH_10_^16–18^ with *T*_C_ > 250 K at 175 GPa.

The neighbor of lanthanum, barium is a promising element for superhydride synthesis. The calculated maximum *T*_C_ is only about 30–38 K^19,20^ for predicted *P*4*/mmm*-BaH_6_ stable at 100 GPa, which has a hydrogen sublattice consisting of H_2_ molecules and H^–^ anions^19^. Lower barium hydride, BaH_2_, well-known for its extraordinarily anionic (H^–^) conductivity^21^, exists in *Pnma* modification below 2.3 GPa, whereas above 2.3 GPa it undergoes a transition to hexagonal Ni_2_In-type *P*6_3_*/mmc* phase^22^. At pressures above 41 GPa, BaH_2_ transforms into *P*6*/mmm* modification, which metallizes at over 44 GPa, but its superconducting *T*_C_ is close to zero^23^. So far, no relevant experiments at pressures above 50 GPa have been reported.

In this work we experimentally and theoretically investigate the chemistry of the barium-hydrogen system at pressures from 75 to 173 GPa filling the gap of previous studies. We discover new pseudocubic BaH_12_ that has molecular structure with H_2_ and H_3_^–^ molecular units and detached H_12_ chains formed due to Peierls-type distortion. These structural features lead to metallic conductivity of unique molecular hydride and to the superconducting transition around 20 K at 140 GPa.

## Results

### Synthesis at 160 GPa and Stability of BaH_12_

To investigate the formation of new chemical compounds in the Ba–H system at high pressures, we loaded four high-pressure diamond anvil cells (DACs #B0-B3) with sublimated ammonia borane NH_3_BH_3_ (AB), used as both a source of hydrogen and a pressure transmitting medium. A tungsten foil with a thickness of about 20 μm was used as a gasket. Additional parameters of the high-pressure diamond anvil cells are given in Supplementary Table S1.

**Fig. 1:**
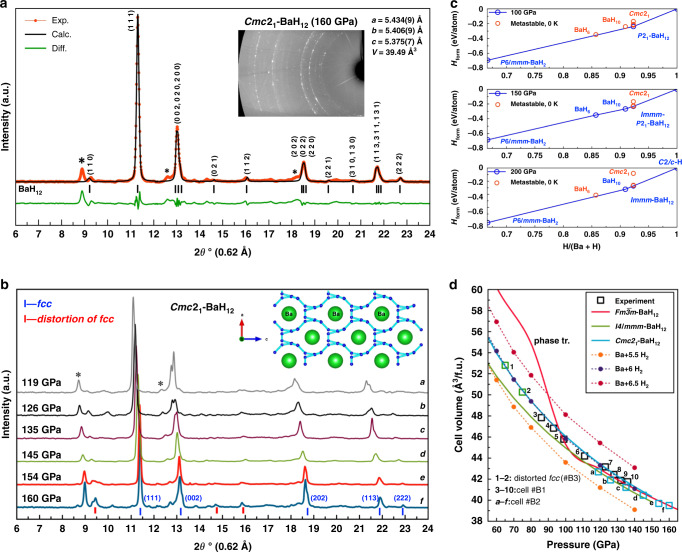
XRD patterns of synthesized samples at various pressures with theoretical analysis of their stability. **a** Experimental X-ray diffraction pattern from DAC #B1 at 160 GPa and the Le Bail refinement of the pseudocubic *Cmc*2_1_-BaH_12_ phase. The experimental data, fitted line, and residues are shown in red, black, and green, respectively. Unidentified reflections are indicated by asterisks. **b** X-ray diffraction patterns at pressures of 119 to 160 GPa. The inset shows the projection of the *Cmc*2_1_ structure to the (*ac*) plane. The hydrogen network is shown by light blue lines. **c** Convex hulls of the Ba–H system at 100, 150, and 200 GPa calculated with zero-point energy (ZPE) contribution. **d** Calculated equations of state for different possible crystal modifications of BaH_12_ (*fcc*, *I*4*/mmm*, and *Cmc*2_1_) and Ba+*n*H_2_. The experimental data are shown by hollow squares.

The first attempt of the experimental synthesis was made in DAC #B1 heated to 1700 K by an infrared laser pulse with a duration of ~0.5 s at a pressure of 160 GPa. During heating, the Ba particle underwent significant expansion and remained nontransparent. The obtained synchrotron X-ray diffraction pattern (XRD, λ = 0.62 Å, Fig. 1a) consists of a series of strong reflections specific to cubic crystals. Decreasing the pressure in DAC #B1 to 119 GPa (Fig. 1b) gave a series of diffraction patterns that can mostly be indexed by a slightly distorted face-centered cubic structure (e.g., pseudocubic *Cmc*2_1_, Fig. 1a). Recently, similar cubic diffraction patterns have been observed at pressures above 150 GPa for the La–H (*fcc*-LaH_10_)^17,18^ and Th-H (*fcc*-ThH_10_)^14^ systems. By analogy with the La–H system, and considering the lack of previously predicted cubic superhydrides BaH_*x*_^19–21^, we used the USPEX code^24–26^ to perform theoretical crystal structure evolutionary searches, both variable- and fixed-composition, for stable Ba–H compounds at pressures of 100–200 GPa and temperatures of 0–2000 K.

According to the USPEX calculations, *P*6*/mmm-*BaH_2_ remains stable up to 150–200 GPa (Fig. 1c; Supplementary Tables S7–S12, Supplementary Figs. S2 and S3). This compound was experimentally detected in DAC #B0 at 173–130 GPa with the cell volume ~3% smaller than theoretically predicted (Supplementary Table S14). At 100–200 GPa, several new barium polyhydrides lying on or near the convex hulls were found: BaH_6_, BaH_10_, and BaH_12_ with the unit cell Ba_4_H_48_ and Ba_8_H_96_ (Fig. 1c). In subsequent experiments at 142 and 154–173 GPa we have detected a series of reflections that can be indexed by BaH_6_ and BaH_10_ with the unit cell volumes close to the calculated ones (see Supporting Information, p. S25-27). However, the main phase in almost all diffraction patterns is the pseudocubic barium superhydride which will be described below.

The analysis of the experimental data within space group $Fm\bar 3m$ (Fig. 1b and Supplementary Table S3) of Ba-sublattice and its comparison with density functional theory (DFT) calculations show that the stoichiometry of barium hydride synthesized in DAC #B1 is close to BaH_12_. Examining the results of the fixed-composition search, we found that an ideal $Fm\bar 3m$-BaH_12_ (similar to *fcc*-YB_12_) is unstable and cannot exist, while pseudocubic *P*2_1_-BaH_12_, whose predicted diffraction pattern is similar to the experimental one, lies on the convex hull at 100–150 GPa. There are also pseudocubic *P*1-Ba_8_H_96_, located very close to the convex hull at 150 GPa, and *Cmc*2_1_-BaH_12_ (= Ba_4_H_48_) with a similar X-ray diffraction (XRD) pattern, lying a bit farther. Above 190 GPa the *P*2_1_-BaH_12_ transforms to other possible candidate, orthorhombic *Immm*-BaH_12_, which stabilizes between 150 and 200 GPa, but does not correspond to the experimental XRD pattern (Fig. 1a, Supplementary Fig. S1) and is not considered further.

**Fig. 2:**
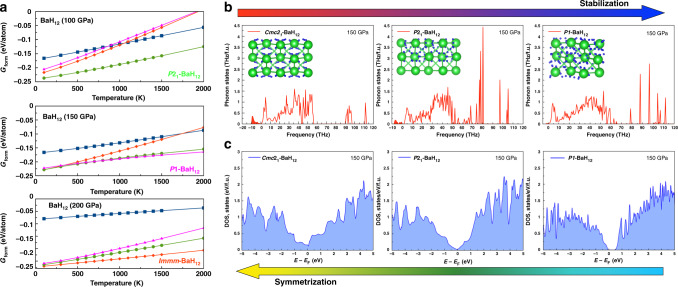
Theoretical study of stability and electronic properties. **a** Dependence of the Gibbs free energies of formation on the temperature for different modifications of BaH_12_ — *Immm*, pseudocubic *Cmc*2_1_, *P*2_1_, and *P*1 — calculated within the harmonic approach in the temperature range of 100 to 2000 K at 100, 150, and 200 GPa. **b** Phonon and **c** electron densities of states for pseudocubic BaH_12_ structures with various degrees of distortion: *Cmc*2_1_, *P*2_1_, and *P*1.

The computed equation of state of $Fm\bar 3m$-BaH_12_ (Fig. 1d) corresponds well to the experimental volume-pressure dependence above 100 GPa. However, the DFT calculations show that the ideal $Fm\bar 3m$ barium sublattice is unstable (it is > 0.19 eV/atom above the convex hull, Supplementary Fig. S4) both thermodynamically and dynamically, and transforms spontaneously to *Cmc*2_1_ or *P*2_1_ via distortion (Fig. 2). Studying the temperature dependence of the Gibbs free energy (Fig. 2a), we found that *P*2_1_-BaH_12_ is the most stable modification at 0–2000 K and 100–150 GPa. Moreover, high-symmetry cubic phases cannot explain the weak reflections at 8.9–9.4°, 14.5, 16, 19.5, and 20.6° present in many XRD patterns (Fig. 1a, b).

To clarify the question of dynamical stability of pseudocubic structures, we calculated a series of phonon densities of states for different modifications of BaH_12_ (Fig. 2b). Within the harmonic approach, symmetric and corresponding to the experimental data *Cmc*2_1_-BaH_12_ has a number of imaginary phonon modes. Its distortion to much more stable *P*2_1_-BaH_12_ leads to the disappearance of many of the imaginary phonon modes and deepening of the pseudogap (Fig. 2c) in the electronic density of states *N*(*E*). The subsequent distortion of *P*2_1_ to *P*1 converts BaH_12_ to a semiconductor with a bandgap exceeding 0.5 eV. However, the experimental data show that BaH_12_ remains opaque in the visible range, does not give Raman signals (Supplementary Figs. S39-40), retains an almost *fcc* crystal structure, and exhibits metallic properties (see next sections) down to 75 GPa. For this reason, the electronic band structure and parameters of the superconducting state were further investigated only for *Cmc*2_1_-BaH_12_, which does not have a bandgap at 100–150 GPa. Stability of all considered polymorphic modifications of BaH_12_ at different pressures with respect to other Ba-H phases and with respect to each other are shown in Supplementary Figs. S4 and S5 (see Supporting Information).

The comparative analysis of *Cmc*2_1_, *P*2_1_, and *P*1 structures of BaH_12_ shows that semimetallic *Cmc*2_1_ explains well the experimental results of X-ray diffraction (see Supplementary Fig. S1, Supporting Information) and lies closer to the convex hull than $Fm\bar 3m$ or *I*4*/mmm* modifications. *P*1-BaH_12_ shows a complex picture of splitting of the diffraction signals, both *P*1-BaH_12_ and *P*2_1_-BaH_12_ have a bandgap above 0.5 eV at 100 GPa (Supplementary Fig. S38b) which does not correspond to the experimental data. Therefore, pseudocubic *Cmc*2_1_-BaH_12_, whose cell volume is near that of the close-packed $Fm\bar 3m$-BaH_12_, is the appropriate explanation of the experimental results despite the presence of a few imaginary phonon modes.

The molecular dynamics simulation of *Cmc*2_1_-BaH_12_ and *P*2_1_-BaH_12_ at 10–1500 K, after averaging the coordinates, both lead to a distorted pseudocubic *P*1-BaH_12_ with the similar XRD pattern. However, all structures retrieved by molecular dynamics are less stable both dynamically and thermodynamically than *P*1-BaH_12_, *P*2_1_-BaH_12_, and *Cmc*2_1_-BaH_12_ found by USPEX. More accurate analysis accounting for the anharmonic nature of hydrogen oscillations^27^, which is actually beyond the scope of this work, may help to explain the experimental stability of higher-symmetry BaH_12_ modifications compared to lower-symmetry *P*1-BaH_12_.

### Synthesis of BaH_12_ at 146 GPa

**Fig. 3:**
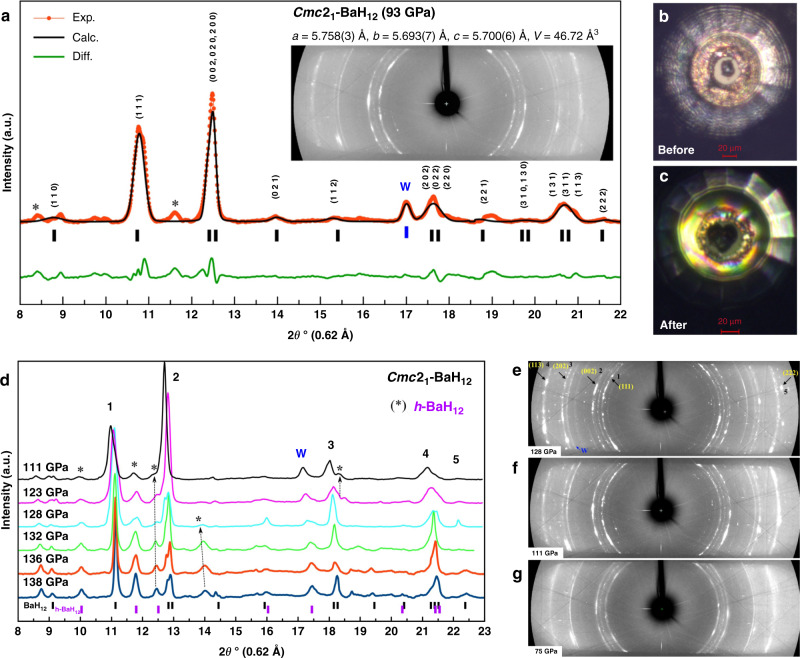
Analysis of experimental XRD patterns of *Cmc*2_1_-BaH_12_ synthesized in DAC #B2. **a** Le Bail refinement of pseudocubic *Cmc*2_1_-BaH_12_ and *bcc*-W at 93 GPa. The experimental data, fitted line, and residues are shown in red, black, and green, respectively. Inset shows the 2D diffraction image. Weak reflections, indicated by asterisks, may correspond to the impurity: possibly *P*6_3_*/mmc* or *P*6_3_*mc*-BaH_12_. **b**, **c** Microphotographs of the culet of cell #B2 with the Ba/AB sample before and after the laser heating. **d** Experimental XRD patterns from cell #B2 at pressures decreasing from 138 to 111 GPa. **e**, **f**, **g** Powder X-ray diffraction images at 128, 111, and 75 GPa. Reflections corresponding to the crystallographic planes in the ideal $Fm\bar 3m$-BaH_12_ phase are indicated by arrows.

Similar X-ray diffraction patterns were obtained in the next experiment (DAC #B2) where the Ba sample was heated at an initial pressure of 146 GPa, which led to a decrease in pressure to 140 GPa. During the heating and subsequent unloading of the cell, the sample remained opaque down to ~40 GPa. Unlike the synthesis at high pressure (cell #B1, 160 GPa, Fig. 1a, b), in this experiment we observed many more side phases and corresponding side reflections than before (Fig. 3 and Supporting Information).

Similar to the experiment with DAC #B1, five reflections from the pseudocubic Ba sublattice dominate in a wide range of pressures (65–140 GPa), whereas side reflections change their intensities and, at some pressures, almost disappear (Fig. 3d and Supplementary Figs. S33 and S35). The diffraction circles corresponding to the ideal cubic barium sublattice have pronounced granularity (Fig. 3e–g, Supplementary Fig. S34), which suggests that all “cubic” reflections belong to the same phase.

At pressures below 65 GPa, it is no longer possible to refine the cell parameters of pseudocubic BaH_12_. The parameters of the *Cmc*2_1_-BaH_12_ unit cell, refined to the experimental data, are presented in Supplementary Table S6. Fitting this pressure-volume data in the pressure range from 75 to 173 GPa by the third-order Birch–Murnaghan equation of state^28^ gives the cell volume *V*_100_ = 45.47 ± 0.13 Å^3^, bulk modulus *K*_100_ = 305 ± 8.5 GPa, and its derivative with respect to pressure $K_{100}^\prime$ = 3.8 ± 0.48 (the index 100 designates values at 100 GPa). Fitting the theoretical data yields similar values: *V*_100_ = 46.0 Å^3^, *K*_100_ = 315.9 GPa, and $K_{100}^\prime$ = 2.94.

### Synthesis of BaH_12_ at 90 GPa

**Fig. 4:**
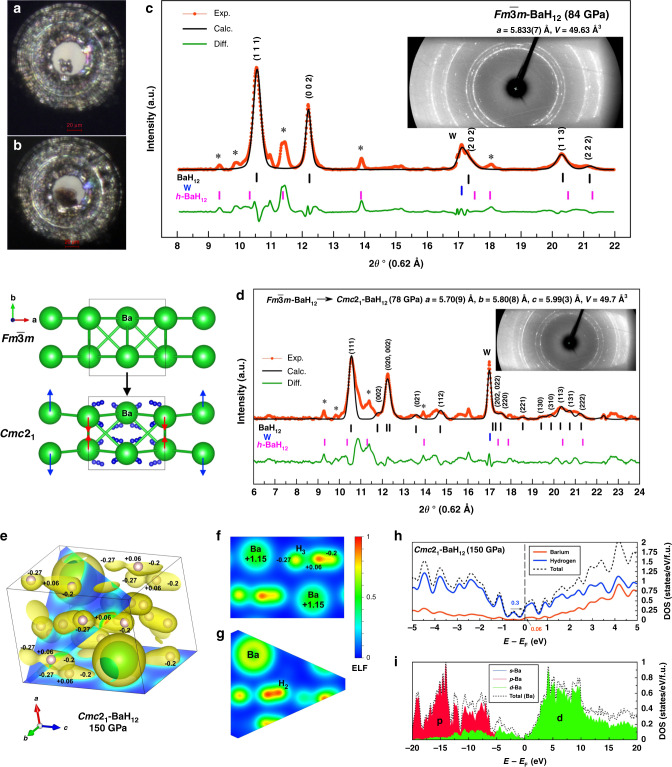
Low-pressure synthesis and physical properties of BaH_12_. Loaded Ba/AB sample **a** before and **b** after the laser heating in DAC #B3. Experimental XRD pattern and the Le Bail refinement of **c** the ideal $Fm\bar 3m$*-*BaH_12_ structure at 84 GPa and **d** *Cmc*2_1_-BaH_12_ obtained via the distortion of $Fm\bar 3m$-BaH_12_. The reflections indicated by asterisks may correspond to unidentified hexagonal barium polyhydride BaH_~12_. The experimental data, model fit for the structure, and residues are shown in red, black, and green, respectively. **e** Electron localization function (ELF), projected onto **f** the (100) plane and **g** the $(11\bar 1)$ plane, and Bader charges of the Ba and H atoms in *Cmc*2_1_-BaH_12_ at 150 GPa. **h** Contribution of barium and hydrogen to the electronic density of states of BaH_12_. **i** d-character of Ba electrons near the Fermi level.

In the experiment with DAC #B3, we investigated the possibility to synthesize BaH_12_ at pressures below 100 GPa. After the laser heating of Ba/AB to 1600 K, the pressure in the cell decreased from 90 to 84 GPa. The observed diffraction pattern is generally similar to those in the previous experiments with DAC #B1, except the presence of the impurity, *h*-BaH_~12_, whose reflections may be indexed by hexagonal space groups *P*6_3_*/mmc* or *P*6_3_*mc* (*a* = 3.955(7) Å, *c* = 7.650(7) Å, *V* = 51.84 Å^3^ at 78 GPa). For the main set of reflections, slightly distorted cubic BaH_12_ is the best solution (Fig. 4). The refined cell parameters of BaH_12_ (Supplementary Table S4) agree well with the results obtained previously with DACs #B1 and B2. When the pressure was reduced to 78 GPa, barium dodecahydride began to decompose, and subsequent diffraction patterns (e.g., at 68 GPa, see Supporting Information) show a complex image of broad reflections that confirms the lower experimental bound of BaH_12_ stability of ~75 GPa mentioned above.

## Discussion

### Electronic properties of BaH_12_

BaH_12_ is the first known metal hydride with such a high hydrogen content that is stable at such low pressures (~75 GPa). We further investigated its electronic structure and the charge state of the hydrogen and barium atoms. The electron localization function (ELF) analysis^29^ (Fig. 4e–g) shows that hydrogen in BaH_12_, similar to NaH_7_^30^, is present in the form of H_2_ (*d*_H–H_ = 0.78 Å) and almost linear H_3_ (*d*_H–H_ = 0.81 and 1.07 Å) molecular fragments that form separate flat horseshoe-like H_12_ chains (*d*_H–H_ < 1.27 Å, Fig. 4).

Bader charge analysis of *Cmc*2_1_-BaH_12_, performed in accordance with our previous experience^31,32^ (Supplementary Table S18), shows that the Ba atoms serve as a source of electrons for the hydrogen sublattice. The charge of the barium atoms in BaH_12_ is +1.15 at 150 GPa, whereas most of the hydrogen atoms have a negative charge. In the H_3_ fragments, the charge of the end atoms is close to –0.2 and –0.27, while the H bridge has a small positive charge of +0.06 (Fig. 4e–g). In general, H_3_^–^ anion, similar to one found in the structure of NaH_7_^30^, has a total charge of –0.4 | *e* | , whereas molecular fragments H_2_ (*d*_H–H_ = 0.78 Å) have a charge of only –0.1 | *e* | . Therefore, the Ba–H bonds in BaH_12_ have substantial ionic character, whereas the H–H bonds are mainly covalent.

The low electronic density of states *N*(*E*) in semimetallic *Cmc*2_1_-BaH_12_ looks typical for one-dimensional …H–H–H… chains (Fig. 4h, Supplementary Figs. S36 and S38) which are divided into H_2_, H_3_ fragments due to the Peierls-type distortion^33^. In fact, all of the discussed structures of BaH_12_ can be viewed as a result of Peierls-type distortion. The main contribution to *N*(*E*_F_), 83% at 150 GPa, comes from hydrogen (Fig. 4h), and ¾ of this contribution is related to s orbitals. At 150 GPa, barium in BaH_12_ exhibits the properties of a d-block element, and its bonding orbitals have a significant d-character (Fig. 4i). Electrical conductivity is localized in the H layers consisting of quasi-one-dimensional …H–H–H… chains which are interconnected in non-trivial way (Fig. 4e–g, Supplementary Table S2 for crystal structure). Thus, barium dodecahydride is the first known molecular superhydride with metallic conductivity embedded in layers and one-dimensional chains of molecular hydrogen.

### Superconductivity of BaH_12_

**Fig. 5:**
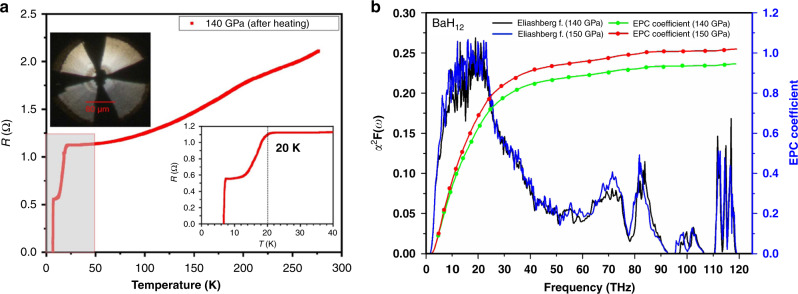
Electrical measurements and calculations of superconducting properties. **a** Four-probe measurements of the electrical  resistance *R*(*T*) of the BaH_*x*_ sample synthesized using the laser heating at 140 GPa. The superconducting transition was detected at ~20 K. **b** Calculated Eliashberg spectral functions and electron-phonon coupling parameter (λ) for pseudocubic BaH_12_ at 140 (black) and 150 GPa (blue).

On the basis of powder diffraction experiments and thermodynamic calculations alone, we cannot unambiguously determine the structure of the H sublattice in BaH_12_, which is essential for understanding superconductivity. To clarify this, we measured the electrical resistance *R* of barium hydride samples using the well-known four-probe technique in the temperature range of 2–300 K. At pressures of 90–140 GPa, all five BaH_*x*_ samples (DACs E#1-5, see Supporting Information) synthesized in electric DACs behave as typical metals with an almost linear decrease of *R*(*T*). At low temperatures the resistance of the samples drops sharply, indicating a possible superconducting transition at about 5–7 K below 130 GPa (Supplementary Fig. S41), and ~20 K at 140 GPa (cell #E5, Fig. 5a). This DAC #E5 was assembled with an 80 µm diamond anvil culet, *c*-BN/epoxy insulating gasket, 45 × 32 µm Ba piece, and sputtered 0.5 µm thick Mo electrodes. After the laser heating at 1600 K and 140 GPa, the Ba/AB sample demonstrated the superconducting transition at around 20 K (Fig. 5a). When we tried to change the pressure, the cell collapsed and pressure dropped to 65 GPa.

The obtained data together with the measured Raman spectra and optical microscopy exclude low-symmetry BaH_12_ semiconducting structures, leaving for consideration only metallic and semimetallic modifications (Supplementary Figs. S39 and S40).

The harmonic DFT calculations (Fig. 5b) demonstrate that the low density of electronic states near the Fermi level in *Cmc*2_1_-BaH_12_ is associated with a weak electron-phonon coupling, mostly related to low-frequency Ba and H phonon modes (1–10 THz), resulting in relatively low λ = 1.02, ω_log_ = 677 K, *T*_C_ = 39–53 K, and µ_0_*H*_c2_(0) = 5.1–7 T at 150 GPa (µ* = 0.15–0.1). Decreasing pressure leads to a decrease of λ and *T*_C_ (from 53 to 46 K) at 140 GPa with a slope *dT*_C_/*dP* = 0.7 K/GPa.

One of the roles of metal atoms in superhydrides is to donate electrons to antibonding orbitals of the H_2_ molecules and weaken the H-H bonds. In BaH_12_, each H atom accepts few electrons, on average 0.16 electrons. As a result, H_2_ and H_3_ groups are still present in the structure, and we have a rather low *T*_*С*_. We think that at high pressures, due to dissociation of molecular groups, BaH_12_ may have a network of weak H-H bonds (rather than discrete H_2_ and H_3_-groups) and, as a result, a much higher *T*_*С*_. Increasing the pressure will also facilitate further metallization of BaH_12_ and symmetrization of the hydrogen sublattice, increasing *N*(*E*_*F*_). To estimate the possible improvement, we calculated at 120-135 GPa the superconducting parameters of *I*4*/mmm*-BaH_12_ and $Fm\bar 3m$-BaH_12_, isostructural to YB_12_, the structures that were considered as possible solutions at the first step of the XRD interpretation. The calculations show that filling of the pseudogap in *N*(*E*) makes it possible to reach *T*_C_ ~ 200 K with λ ≥ 3 in these compounds (Supplementary Figs. S42 and S43).

In conclusion, studying the high-pressure chemistry of the Ba–H system in four independent DACs we successfully synthesized novel barium superhydride BaH_12_ with a pseudocubic crystal structure, stabilized in the pressure range of 75–173 GPa. The compound was obtained by laser-heating metallic barium with an excess of ammonia borane compressed to 173, 160, 146, and 90 GPa. The Ba sublattice structure of BaH_12_ was resolved using the synchrotron XRD, evolutionary structure prediction, and several postprocessing Python scripts, including an XRD matching algorithm. Discovered BaH_12_ has unique metallic conductivity, localized in the layers of molecular hydrogen, and the highest hydrogen content (>92 mol%) among all metal hydrides synthesized so far. The experimentally established lower limit of stability of barium dodecahydride is 75 GPa. The third-order Birch–Murnaghan equation of state and unit cell parameters of BaH_12_ were found in the pressure range of 75–173 GPa: *V*_100_ = 45.47 ± 0.13 Å^3^, *K*_100_ = 305 ± 8.5 GPa, and $K_{100}^\prime$ = 3.8 ± 0.48. The ab initio calculations confirm a small distortion of the ideal *fcc*-barium sublattice to space group *Cmc*2_1_ or *P*2_1_, determined by the presence of additional weak reflections in the diffraction patterns. The impurity phase analysis indicates possible presence of BaH_6_ and BaH_10_. According to the theoretical calculations and experimental measurements, BaH_12_ exhibits metallic and superconducting properties, with *T*_C_ = 20 K at 140 GPa, and its crystal structure contains H_2_ and H_3_^–^ groups. The results of these experiments confirm that the comparative stability of superhydrides increases with the increase of the period number of a hydride-forming element in the periodic table^20^. Our work opens prospects for the synthesis of even more hydrogen rich compounds like predicted LaH_16_^34^ and ErH_15_^20^, and new ternary high-*T*_*C*_ polyhydrides in such systems as Ba-Y-H and Ba-La-H.

## Methods

### Experimental details

The barium metal samples with a purity of 99.99% were purchased from Alfa Aesar. All diamond anvil cells (100 μm and 50 μm culets) were loaded with a metallic Ba sample and sublimated ammonia borane (AB) in an argon glove box. The tungsten gasket had a thickness of 20 ± 2 μm. The heating was carried out by 2–3 pulses of an infrared laser (1.07 μm, Nd:YAG), each pulse had a duration of 0.3–0.5 s. The temperature was determined using the decay of blackbody radiation within the Planck formula. The applied pressure was measured by the edge of diamond Raman signal^35^ using the Horiba LabRAM HR800 Ev spectrometer with an exposure time of 10 s. The XRD patterns from samples in diamond anvil cells (DACs) were recorded on the BL15U1 synchrotron beamline at the Shanghai Synchrotron Research Facility (SSRF, China) using a focused (5 × 12 μm) monochromatic X-ray beam with a linear polarization (20 keV, 0.6199 Å). Mar165 CCD was used as a detector.

The experiment with DAC #B0 was carried out at the Advanced Photon Source, Argonne, U.S. The loaded particle was successively heated up to 2100 K using millisecond-long pulses (4 × 0.04 s) of a 1064 nm Yb-doped fiber laser. We used this pulsed laser heating mode to avoid the premature breakage of a diamond. The synchrotron XRD measurements (the X-ray wavelength was 0.2952 Å) were performed at the GSECARS of the Advanced Photon Source^36^ with about 3 × 4 µm X-ray beam spot.

The experimental XRD images were analyzed and integrated using Dioptas software package (version 0.5)^37^. The full profile analysis of the diffraction patterns and the calculation of the unit cell parameters were performed in the Materials studio^38^ and JANA2006^39^ using the Le Bail method^40^.

To investigate the electrical resistivity of barium polyhydrides, we performed 5 runs of measurements in Cu-Be DACs #E1-5 using the four-probe technique. The preparation of all cells was similar. Tungsten gasket with initial thickness of 250 μm was precompressed to about 25 GPa. Then a hole with a diameter of 20% bigger than the culet diameter was drilled using pulse laser (532 nm). Cubic boron nitride (c-BN) powder mixed with epoxy was used as an insulating layer. We filled the chamber with MgO and compressed it to about 5 GPa. Then, in the obtained transparent MgO layer, a hole with diameter about 40μm was drilled by laser. UV lithography was used to prepare four electrodes on the diamond culet. We deposited the 500 nm thick Mo layer by magnetron sputtering (field of 200 V at 300 K) and removed the excess of metal by acid etching. Four deposited Mo electrodes were extended by platinum foil. The chamber was filled with the sublimated ammonia borane (AB) and a small piece of Ba was placed on the culet of upper diamond with the four electrodes. All preparations were made in an argon glove box (O_2_ < 0.1 ppm, H_2_O < 0.01 ppm). After that, the DACs were closed and compressed to a required pressure. We used 1.07 µm infrared pulse (~0.1 s, 1600 K) laser to heat the Ba/AB samples. Electrical resistance of the samples was studied in a cryostat (1.5-300 K, JANIS Research Company Inc.; in magnetic fields 0-9 T, Cryomagnetics Inc.) with applied current of 1 mA. More details about DACs #E1-5 are given in the Table S20.

### Computational details

The study is based on the structural search for stable compounds in the Ba–H system using the USPEX code, for pressures of 50, 100, 150, 200, and 300 GPa, with a variable-composition evolutionary search from 0 to 24 atoms of each type (Ba, H). The first generation of the search (120 structures) was created using a random symmetric generator, all subsequent generations (100 structures) contained 20% of random structures and 80% of those created using the heredity, soft mutation, and transmutation operators. The results contain the files extended_convex_hull and extended_convex_hull_POSCARS, which were postprocessed using the Python scripts change_pressure.py, split_CIFs.py and xr_screening.py (see Scripts for XRD Postprocessing with USPEX section). The postprocessing script change_pressure.py performs an isotropic deformation of the unit cell of structures predicted by USPEX, bringing them to approximately the experimental pressure. All three lattice constants of the structures are multiplied by a factor k, calculated under the assumption of validity of the Birch–Murnaghan equation of state^28^ with the bulk modulus *K*_*0*_ = 300 GPa and its first derivative *K’* = 3. This approach is a quick alternative to the script that uses a crude DFT reoptimization of a set of theoretically possible structures, bringing them to the experimental pressure. The script split_CIFs.py converts the set of POSCARS recorded in the extended_convex_hull_POSCARS file into a set of CIF files, simultaneously symmetrizing the unit cells and sorting the files by ascending fitness (the distance from the convex hull). The CIF files created in such a way can be directly analyzed using Dioptas^37^, JANA2006^39^ and other software. Finally, the script xr_screening.py automatically searches for the structures found by USPEX and translated to the experimental pressure that exhibit a high similarity between the experimental and predicted XRD patterns (the latter are obtained using pymatgen^41^ Python library). The analysis of complex mixtures consisted of two steps: first, we searched for the main component having the most intense reflections, then the already explained reflections were excluded to analyze the side phases.

To calculate the equations of state (EoS) of BaH_12_, we performed structure relaxations of phases at various pressures using the density functional theory (DFT)^42,43^ within the generalized gradient approximation (Perdew–Burke–Ernzerhof functional)^44^ and the projector augmented wave method^45–49^ as implemented in the VASP code^15–17^. The plane wave kinetic energy cutoff was set to 1000 eV, and the Brillouin zone was sampled using the Γ-centered k-points meshes with a resolution of 2π × 0.05 Å^−1^. The obtained dependences of the unit cell volume on the pressure were fitted by the Birch–Murnaghan equation^28^ to determine the main parameters of the EoS — the equilibrium volume V_0_, bulk modulus K_0_, and its derivative with respect to pressure K’ — using EOSfit7 software^50^. We also calculated the phonon densities of states for the studied materials using the finite displacement method (VASP and PHONOPY)^51,52^.

The calculations of the phonons, electron-phonon coupling, and superconducting *T*_C_ were carried out with QUANTUM ESPRESSO (QE) package^53,54^ using the density functional perturbation theory^55^, employing the plane-wave generalized gradient approximation with Perdew–Burke–Ernzerhof functional^44^. In our ab initio calculations of the electron-phonon coupling (EPC) parameter λ of *Cmc*2_1_-Ba_4_H_48_, the first Brillouin zone was sampled by 2 × 2 × 2 q-points mesh and 4 × 4 × 4 or 8 × 8 × 8 k-points meshes with the smearing σ = 0.005–0.05 Ry that approximates the zero-width limits in the calculation of λ. The critical temperature *T*_C_ was calculated using the Allen–Dynes equations^56^.

Bader charges were calculated using Critic2^57,58^ software with the atomic partition generated using the YT^59^ method. The electron localization function (ELF) of BaH_12_ and isosurface are shown for an isovalue of 0.12. The lattice planes (100) and $\left( {11\bar 1} \right)$ are shown at distances from the origin of 0 and 2.289 Å, respectively. We projected the ELF on these planes to show H_3_ and H_2_ bonding.

The average structure of BaH_12_ was analyzed using the ab initio molecular dynamics (AIMD) simulations within the general gradient approximation^44^ and using the augmented plane wave method^45,47–49^ implemented in VASP software^30–32^. The total number of atoms in the model was 52, including 48 hydrogen atoms and 4 barium atoms (*Cmc*2_1_-Ba_4_H_48_). The positions of the barium atoms were fixed during the simulation. The energy cutoff was set to 400 eV. The behavior of the hydrogen atoms in the BaH_12_ crystal structure was studied upon annealing from 1500 to 10 K using the Nosé–Hoover thermostat^60,61^. The total simulation time was 10 ps with the time step of 1 fs.

## Supplementary information

Supporting Information

Peer Review File

## Data Availability

The authors declare that the data supporting the findings of this study are available within the paper and its supplementary information files.
