# Supplementary material for: Synthesis of molecular metallic barium superhydride: pseudocubic BaH12
Source: Nat Commun. 2021 Jan 11;12:273. doi: 10.1038/s41467-020-20103-5 (PMC7801595; doi:10.1038/s41467-020-20103-5)
Supplement: Supplementary file 1 — Supporting Information [file 41467_2020_20103_MOESM1_ESM.pdf]

Supporting Information

Synthesis of Molecular Metallic Barium Superhydride: Pseudocubic BaH_{12}

Wuhao Chen,[1,†] Dmitrii V. Semenok,[2,†] Alexander G. Kvashnin,[2,†] Xiaoli Huang,[1,*] Ivan A. Kruglov,[3,4] Michele Galasso,[2] Hao Song,[1] Defang Duan,[1] Alexander F. Goncharov,[5] Vitali B. Prakapenka,[6] Artem R. Oganov,[2*] and Tian Cui[7,1,*]

[1] State Key Laboratory of Superhard Materials, College of Physics, Jilin University, Changchun 130012, China
[2] Skolkovo Institute of Science and Technology, Skolkovo Innovation Center, 3 Nobel Street, Moscow 143026, Russia
[3] Moscow Institute of Physics and Technology, 9 Institutsky Lane, Dolgoprudny 141700, Russia
[4] Dukhov Research Institute of Automatics (VNIIA), Moscow 127055, Russia
[5] Earth and Planets Laboratory, Carnegie Institution of Washington, 5251 Broad Branch Road NW, Washington, D.C. 20015, U.S.
[6] Center for Advanced Radiation Sources, The University of Chicago, 5640 South Ellis Avenue, Chicago, Illinois 60637, U.S.
[7] School of Physical Science and Technology, Ningbo University, Ningbo, 315211, China

Corresponding authors
*Prof. A.R. Oganov, a.oganov@skoltech.ru
*Dr. X. Huang, huangxiaoli@jlu.edu.cn
*Prof. T. Cui, cuitian@jlu.edu.cn
†These authors contributed equally to this work.

Contents

Experimental details.................S2
Structural Information.................S3
Thermodynamic Stability of Barium Hydrides.................S8
Le Bail Refinements and XRD Data.................S14
Synthesis at 173 and 154 GPa: Side Products.................S26
Experimental Stability of BaH_{12}.................S29
Electronic Properties of Barium Superhydrides.................S30
Bader Charges.................S31
Raman Spectra.................S32
Temperature Dependence of Resistance.................S34
Superconductivity.................S36
Scripts for XRD Postprocessing with USPEX.................S38
References.................S44

Experimental details

Table S1. Experimental parameters of the DACs that were used to synthesize barium hydrides

Cell #	Synthesis pressure, GPa	Culet size, μm	Sample size, μm	Composition/load
B0	173	50	17	Ba/BH_3NH_3
B1	160	50	18	Ba/BH_3NH_3
B2	146	100	25	Ba/BH_3NH_3
B3	90	100	20	Ba/BH_3NH_3

Structural Information

Table S2. Calculated crystal structure of the discovered or proposed Ba–H phases.

Phase	Pressure, GPa	Lattice parameters	Coordinates			
$Cmc2_1$-BaH_{12}* 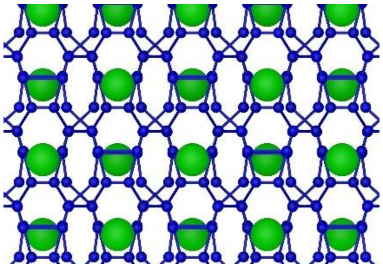	135	a = 5.523 Å<br>b = 5.532 Å<br>c = 5.469 Å<br>α = β = γ = 90°	Ba1	0	-0.27659	0.27659
			H1	0.33839	0.12599	-0.07283
			H2	-0.42628	0.39423	-0.07181
			H3	-0.36584	0.13890	-0.26709
			H4	0.36927	0.40211	-0.28294
			H5	-0.33092	0.11005	-0.40847
			H6	0.32262	0.42112	-0.41622
$P2_1$-Ba_2H_{24}	135	a = 5.462 Å<br>b = 5.514 Å<br>c = 5.514 Å<br>α = γ = 90°<br>β = 89.351°	Ba1	0.4846	0.16288	0.27181
			Ba2	-0.01301	-0.33312	0.22845
			H1	-0.15533	0.09071	0.42612
			H2	0.31936	-0.16502	0.37603
			H3	0.12822	-0.3465	-0.151
			H4	-0.28921	-0.12938	0.44329
			H5	-0.21294	-0.12717	-0.05641
			H6	-0.37488	-0.20024	0.35571
			H7	-0.12458	-0.19785	-0.144
			H8	-0.17247	7.6E-4	0.14518
			H9	-0.3871	-0.20512	0.115
			H10	-0.1306	-0.46983	-0.13562
			H11	0.39717	0.01551	-0.1029
			H12	-0.37675	-0.45958	0.34763
			H13	0.10333	0.00969	0.39987
			H14	0.36826	-0.32842	0.35221
			H15	0.10721	0.16772	0.38177
			H16	0.09193	0.34581	0.41362
			H17	0.33751	-0.48387	0.35569
			H18	-0.45122	0.01689	-0.09278
			H19	0.21009	-0.04841	0.04815
			H20	-0.30083	0.45752	0.43174
			H21	-0.32777	0.17731	-0.06437
			H22	-0.15843	0.3184	0.15846
			H23	-0.1509	0.23521	0.42021
			H24	-0.44591	0.28849	-0.1049

* In general, the $Cmc2_1$-BaH_{12} structure can be represented as a layered crystal with relatively weak links between not only the Ba layers ($d_{Ba–Ba}$ = 2.75 Å), but also between the H layers ($d_{L1–L2}$ = 1.35 Å).

Phase		Pressure,	Lattice parameters	Coordinates			
*P*1-Ba_8H_{96}		150	*a* = 7.727 Å<br>*b* = 7.511 Å<br>*c* = 7.675 Å<br>α = 60.803°<br>β = 118.963°<br>γ = 118.956°	H48	0.17264	0.01277	-0.18272
				H49	0.23144	0.22148	0.11669
				H50	0.05565	-0.17192	-0.16543
				H51	-0.34542	-0.14652	-0.1522
				H52	0.06725	0.14151	0.20013
				H53	0.22976	-0.2849	0.1272
Coordinates							
Ba1	0.48696	0.46107	0.48313	H54	-0.04364	0.29346	-0.19771
Ba2	-0.00691	-0.03146	-0.01115	H55	0.30128	-0.05179	0.17834
Ba3	0.48927	-0.04802	0.49273	H56	-0.24055	0.07344	-0.27219
Ba4	-0.00643	0.45997	-0.00329	H57	-0.30543	-0.13609	0.03353
Ba5	-0.00514	-0.49087	0.4946	H58	-0.08256	-0.17134	0.29316
Ba6	0.49522	0.02179	-0.00318	H59	0.14913	0.36582	-0.17646
Ba7	0.00365	0.02142	0.49413	H60	0.24599	0.1425	-0.13413
Ba8	0.49604	-0.4998	0.0075	H61	-0.21699	-0.3592	-0.10294
H1	-0.34209	0.35848	-0.17877	H62	0.18795	0.29434	0.30678
H2	0.22995	-0.44111	0.25528	H63	-0.48507	0.30054	0.32906
H3	-0.46098	0.12896	-0.30954	H64	-0.19087	-0.00765	-0.34461
H4	0.31709	-0.39014	-0.49048	H65	-0.15169	0.20186	-0.12599
H5	0.34692	0.14461	-0.32254	H66	0.09885	-0.29727	0.22767
H6	0.19972	0.33228	-0.4283	H67	-0.2552	0.21875	-0.39141
H7	-0.20358	-0.30211	0.4269	H68	0.36188	0.07504	0.1536
H8	-0.47811	-0.30631	0.19236	H69	0.30738	0.20284	-0.04212
H9	-0.37977	-0.39253	-0.26799	H70	0.06868	0.20361	-0.31799
H10	0.28026	0.1169	0.41818	H71	-0.17524	-0.37418	0.172
H11	0.33358	-0.48137	0.14774	H72	-0.21935	-0.05563	0.20032
H12	-0.3248	-0.19432	0.48291	H73	0.32315	-0.31976	0.00116
H13	-0.27065	-0.3905	0.35535	H74	-0.04433	-0.31569	-0.36633
H14	-0.26159	-0.26906	-0.41683	H75	0.33963	-0.37682	-0.32999
H15	0.36621	0.39084	-0.27358	H76	0.211	-0.16227	-0.41188
H16	0.27969	0.21442	-0.34396	H77	0.2513	-0.25133	-0.1006
H17	0.27468	0.38365	0.20761	H78	0.19214	-0.42629	-0.18797
H18	-0.23734	-0.40572	-0.26686	H79	0.17345	-0.15866	0.47458
H19	-0.48413	-0.36554	-0.32179	H80	0.37917	-0.13532	-0.24147
H20	0.34374	0.1033	-0.46152	H81	0.4733	-0.17866	-0.15826
H21	0.38624	-0.23863	0.28298	H82	0.07129	-0.29489	-0.29782
H22	-0.18594	-0.4807	-0.35021	H83	0.27055	-0.35731	-0.26904
H23	0.25587	-0.40761	0.39794	H84	0.23634	-0.11771	-0.25086
H24	-0.3269	0.28519	-0.21274	H85	-0.27111	0.36523	0.09828
H25	-0.16278	-0.37708	0.01406	H86	0.07859	0.37219	0.34759
H26	-0.05301	0.11145	0.14803	H87	-0.33498	0.46772	0.32823
H27	0.39536	0.24377	0.28409	H88	-0.33289	-0.01163	0.33692
H28	-0.20378	-0.13054	-0.30565	H89	-0.18774	0.17815	0.17482
H29	-0.20998	0.11799	-0.03176	H90	-0.04538	0.33535	0.31225
H30	0.00261	-0.39347	0.1776	H91	-0.30454	0.24534	0.32361
H31	-0.32844	0.28961	-0.48155	H92	-0.43817	0.20147	0.20768
H32	-0.49505	-0.1924	0.31467	H93	-0.34617	0.35763	-0.02808
H33	0.46667	0.35674	-0.19715	H94	-0.20441	0.1941	0.4434
H34	-0.04947	0.17444	-0.34218	H95	-0.19793	0.4287	0.20345
H35	-0.21024	-0.29015	0.16478	H96	-0.26232	0.12266	0.25664
H36	-0.28389	-0.12742	0.28126				
H37	0.17498	0.30041	0.01259				
H38	-0.06383	-0.23466	-0.20918				
H39	-0.32774	-0.21439	-0.18933				
H40	0.21155	0.05533	0.27503				
H41	0.18291	-0.19436	0.02756				
H42	-0.19054	0.40261	-0.31848				
H43	0.18641	-0.18923	0.30159				
H44	-0.37423	0.06867	-0.23703				
H45	-0.3929	-0.24806	0.12955				
H46	0.03416	-0.11713	0.31037				
H47	0.22253	0.38199	-0.2341				

Phase	Pressure, GPa	Lattice	Coordinates			
Fm$\bar{3}$*m*-BaH_{12}* 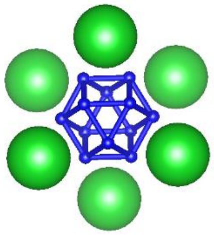	135	a = 5.4830 Å α = β = γ = 90°	Ba1	0	0	0
			H1	0.5	0.16374	0.16374
*I*4/*mmm*-BaH_{12}** 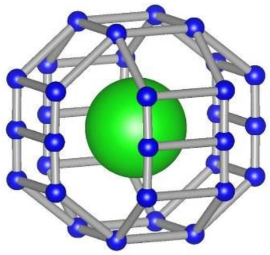	135	a = 3.857 Å c =5.522 Å α = β = γ = 90°	Ba1	0	0	0.5
			H1	-0.274	0	0
			H2	0	-0.28045	0.16392

**fcc*-BaH_{12} isostructural with YB_{12}. Several cubic structures may be proposed to explain the experimental XRD patterns, for instance, *Pm*$\bar{3}$-BaH_{12}. However, the latter one has a much lower theoretical volume compared with the experimental one (e.g., at 160 GPa the calculated volume of *Pm*$\bar{3}$-BaH_{12} is 37.68 Å^3, whereas the experimental one is 39.49 Å^3).

** This phase was used in the modeling and for comparison. Tetragonal *I*4/*mmm*-BaH_{12} (with the unit cell Ba_2H_{24}) may explain most of the weak reflections, but *Cmc*2_1 and *P*2_1-BaH_{12} lie closer to the convex hull at 100–150 GPa and are close to achieving the dynamic stability. At the same time, tetragonal BaH_{12}, lying more than 0.1 eV/atom above the convex hull, is unstable both dynamically and thermodynamically.

Phase	Pressure, GPa	Lattice parameters	Coordinates			
*P*2/*m*-BaH_{10}	142	a = 3.718 Å b = 3.838 Å c = 5.444 Å α = γ = 90° β = 107.012°	Ba1	0.4607	0.0	-0.2554
			H1	0.0373	0.2139	-0.0622
			H2	0.2218	0.5	-0.2895
			H3	0.2596	0.5	-0.1326
			H4	0.3935	0.5	0.4999
			H5	0.0959	0.2510	0.3577
			H6	0.0	0.2889	0.5
			H7	-0.2241	0.5	-0.2209
			H8	-0.3078	0.5	-0.1016

Table S3. Experimental cell parameters of the refined $Fm\bar{3}m$-BaH_{12} structure compared with the DFT results. Volumes are given per Ba atom.

Pressure, GPa	a, Å	V, Å^3	V_{DFT}, Å^3
160	5.4065	39.51	39.48
154	5.4136	39.66	39.90
145	5.4545	40.57	40.68
135	5.4785	41.10	41.54
126	5.5126	41.88	42.38
119	5.5400	42.51	43.04
111	5.6111	44.17	43.97
99	5.6763	45.72	45.37
93	5.7200	46.79	46.08
86	5.7569	47.70	46.95
75	5.8530	50.13	48.41

Table S4. Experimental cell parameters of the refined pseudocubic $Cmc2_1$-BaH_{12} structure (DAC #B3). Volumes are given per Ba atom. When the pressure decreases below 78 GPa, the refinement using this structure is no longer possible.

Pressure, GPa	a, Å	b, Å	c, Å	V, Å^3	V_{DFT}, Å^3
84	5.833(7)	5.833(7)	5.833(7)	49.63	48.70
78	5.70(9)	5.80(8)	5.99(3)	49.7	49.78

Table S5. Experimental cell parameters of the refined pseudocubic $Cmc2_1$-BaH_{12} structure (Fig. 1 and 3). Volumes are given per Ba atom. When the pressure decreased below 119 GPa, the diamond anvil cell broke.

Pressure, GPa	a	b	c	V	V
160	5.434(9)	5.406(9)	5.375(7)	39.49	39.48
154	5.442(5)	5.417(3)	5.384(1)	39.69	39.99
145	5.480(8)	5.416(3)	5.453(2)	40.47	40.80
135	5.485(5)	5.502(6)	5.463(3)	41.22	41.77
126	5.561(0)	5.507(1)	5.475(5)	41.92	42.74
119	5.585(9)	5.537(9)	5.520(3)	69	43.53

* Some deviations (~1–2%) of the experimental cell volume obtained in DACs #B1 and B2 from the calculated values at 132–138 GPa may be due to either errors or irregularities in the pressure measurements when the pressure decreases in small steps, or small variations in stoichiometry (±0.25H).

Table S6. Experimental cell parameters of refined pseudocubic *Cmc*2$_1$-BaH_{12} and proposed *h*-BaH_{12} (impurity). Volumes are given per Ba atom. When the pressure decreases below 75 GPa, the refinement using this structure is no longer possible. Below 38 GPa, the diamond anvil cell broke.

	Pressure, GPa	*a*, Å	*b*, Å	*c*, Å	*V*, Å^3	V_{DFT}, Å^3
*Cmc*2$_1$-BaH_{12}	140	5.500(1)	5.481(6)	5.539(3)	41.75	41.27
	138	5.526(9)	5.567(4)	5.500(3)	42.31	41.45
	136	5.535(2)	5.576(2)	5.517(7)	42.58	41.68
	132	5.546(4)	5.595(6)	5.510(8)	42.75	42.07
	128	5.542(1)	5.590(4)	5.505(0)	42.64	42.50
	123	5.560(1)	5.606(3)	5.525(9)	43.06	43.05
	111	5.602(6)	5.638(8)	5.571(2)	44.00	44.50
	99	5.706(5)	5.665(6)	5.637(4)	45.57	46.21
	93	5.758(3)	5.693(7)	5.700(6)	46.72	47.12
	86	5.759(9)	5.812(0)	5.717(9)	47.85	48.30
	75	5.831(0)	5.891(8)	5.797(2)	49.72	50.37
h-BaH_{12}	140	4.0798	4.0798	5.7151	41.19	40.97
	136	4.0998	4.0998	5.7151	41.59	41.32
	132	4.1050	4.1050	5.7351	41.85	41.71
	111	4.1250	4.1250	5.7751	42.55	43.99

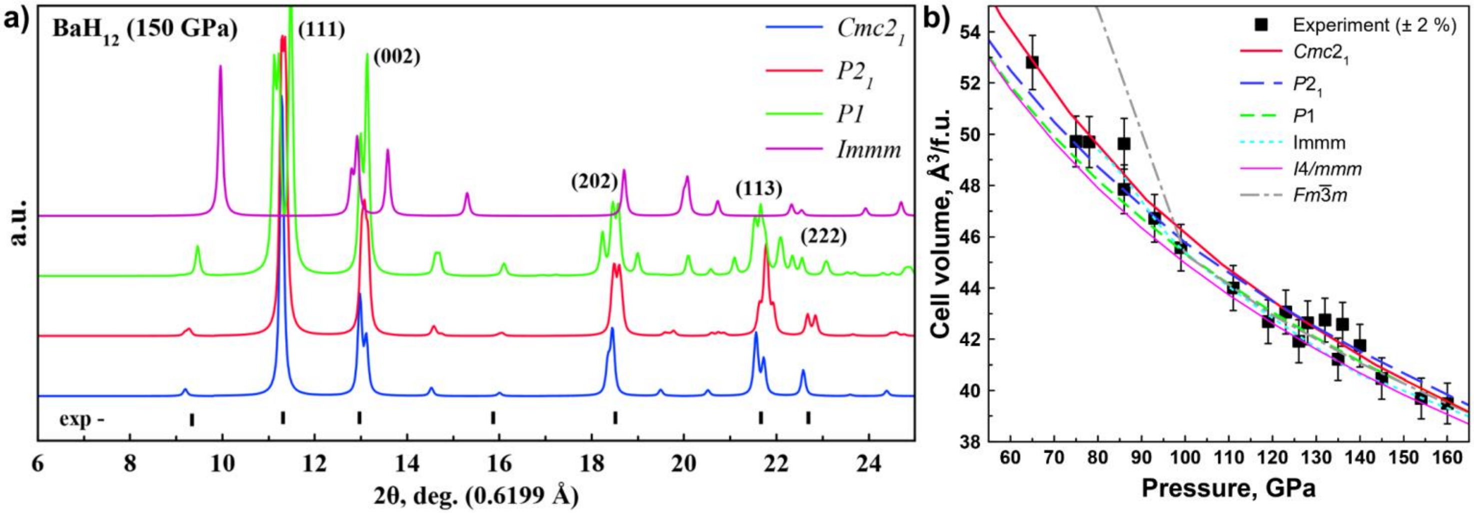

Fig. S1. (a) Comparison of the predicted XRD patterns of DFT relaxed (PAW_PBE) structures of *Cmc*2$_1$-, *P*2$_1$-, *P*1- and *Immm*-BaH_{12} at 150 GPa. (b) Comparison of the predicted equations of state of different modifications of BaH_{12} with the experimental data.

Thermodynamic Stability of Barium Hydrides

We computed the Gibbs free energy as:

$$G(T) = E_0 + PV + k_B T \int g(\omega) \ln\left[1 - \exp\left(-\frac{\hbar\omega}{k_B T}\right)\right] d\omega + \frac{1}{2}\int g(\omega)\hbar\omega d\omega, \quad \text{(S1)}$$

where $E_0 + PV$ is the total energy from the DFT calculations, $g(\omega)$ is the phonon density of states at a given pressure calculated using the finite displacements method as implemented in PHONOPY,[17,18] with forces computed using VASP.[33–35]

The molecular dynamics simulation of $Cmc2_1$-BaH_{12} and $P2_1$-BaH_{12} at 10–1500 K, after averaging the coordinates, leads to distorted pseudocubic $P1$-BaH_{12} with the same XRD pattern. All structures retrieved by molecular dynamics are less stable both dynamically and thermodynamically than $P1$-BaH_{12}, $P2_1$-BaH_{12}, and $Cmc2_1$-BaH_{12} found by USPEX.

Table S7. Enthalpy and energy of formation with and without the ZPE for various Ba–H phases at 100 GPa.

Phase	Ba	H	x (H/H+Ba)	E, eV	E per atom, eV/atom	ZPE, eV/atom	E+ZPE, eV/atom	H_{form}, eV/atom
$P6_3/mmc$-Ba	2	0	0	27.6673	13.8336	0.0048	13.8384	0
$P6/mmm$-BaH_2	4	8	0.666	37.5762	3.1313	0.00753	3.1388	-0.693
$Imm2$-BaH_6	2	12	0.857	8.2995	0.5928	0.03449	0.6273	-0.346
$Cmmm$-BaH_{10}	2	20	0.909	-1.8225	-0.0828	0.03759	-0.0452	-0.239
$Fm\bar{3}m$-BaH_{12}	1	12	0.923	2.4031	0.1848	0.03435	0.2192	0.235
$I4/mmm$-BaH_{12}	1	12	0.923	-2.7966	-0.2151	0.03554	-0.1795	-0.163
$Immm$-BaH_{12}	1	12	0.923	-3.5139	-0.2703	0.03396	-0.2363	-0.220
$Cmc2_1$-BaH_{12}	4	48	0.923	-11.4236	-0.2196	0.03541	-0.1842	-0.168
$P2_1$-BaH_{12}	4	48	0.923	-14.8417	-0.2854	0.03108	-0.2543	-0.238
$P1$-BaH_{12}	8	96	0.923	-29.0781	-0.2795	0.05466	-0.2795	-0.209
$C2/c$-H	0	24	1	-28.9758	-1.2073	0.036978	-1.1703	0

Table S8. Enthalpy and energy of formation with and without the ZPE for various Ba–H phases at 150 GPa.

Phase	Ba	H	x (H/H+Ba)	E, eV	E per atom, eV/atom	ZPE, eV/atom	E+ZPE, eV/atom	H_{form}, eV/atom
$P6_3/mmc$-Ba	2	0	0	38.7429	19.37147	0.00539	19.3768	0
$P6/mmm$-BaH_2	4	8	0.666	65.1135	5.426130	0.00782	5.43395	-0.684
$Imm2$-BaH_6	2	12	0.857	27.1473	1.939094	0.04106	1.98015	-0.350
$Cmmm$-BaH_{10}	2	20	0.909	21.8757	0.994335	0.03609	1.03043	-0.266
$Fm\bar{3}m$-BaH_{12}	1	12	0.923	15.6908	1.20691	0.03445	1.24144	0.222
$I4/mmm$-BaH_{12}	1	12	0.923	10.5822	0.81402	0.03854	0.85256	-0.167
$Immm$-BaH_{12}	1	12	0.923	9.726517	0.74819	0.04028	0.78719	-0.231
$Cmc2_1$-BaH_{12}	4	48	0.923	41.4804	0.7977	0.037256	0.834956	-0.182
$P2_1$-BaH_{12}	4	48	0.923	38.61635	0.74262	0.045935	0.78855	-0.230
$P1$-BaH_{12}	8	96	0.923	77.98175	0.74982	0.044537	0.79436	-0.224
$C2/c$-H	0	24	1	-13.3079	-0.55449	0.043624	-0.51086	0

Table S9. Enthalpy and energy of formation with and without the ZPE for various Ba–H phases at 200 GPa.

Phase	Ba	H	x (H/H+Ba)	E, eV	E per atom, eV/atom	ZPE, eV/atom	E+ZPE, eV/atom	H_{form}, eV/atom
$P6_3/mmc$-Ba	2	0	0	49.84141	24.920703	0.006796	24.9274	0
$P6/mmm$-BaH_2	4	8	0.666	90.10875	7.5090629	0.010282	7.519344	-0.830
$Imm2$-BaH_6	2	12	0.857	44.1656	3.154686	0.046283	3.200969	-0.412
$Cmmm$-BaH_{10}	2	20	0.909	43.17913	1.9626875	0.036142	1.998830	-0.322
$Fm\bar{3}m$-BaH_{12}	1	12	0.923	27.6961	2.1304	0.03481	2.16528	0.191
$I4/mmm$-BaH_{12}	1	12	0.923	22.2309	1.7101	0.04055	1.75062	-0.223
$Immm$-BaH_{12}	1	12	0.923	21.58738	1.6605681	0.041234	1.701802	-0.271

Phase	Ba	H	x (H/H+Ba)	E, eV	E per atom, eV/atom	ZPE, eV/atom	E+ZPE, eV/atom	H_{form}, eV/atom
*Cmc*2₁-BaH₁₂	4	48	0.923	88.6132	1.7041	0.044276	1.748376	-0.229
*P*2₁-BaH₁₂	4	48	0.923	86.41456	1.6618185	0.04593	1.707754	-0.265
*P*1-BaH₁₂	8	96	0.923	1	1.6704903	0.04249	1.670490	-0.260
*C*2/*c*-H	0	24	1	0.364101	0.0151709	0.0456539	0.060824	0

Table S10. Temperature dependence of the Gibbs free energy of formation (G_{form}, eV/atom), computed with the ZPE for various Ba–H phases at 100 GPa.

Temperature, K	100	300	500	700	900	1100	1300	1500	2000
$P6_3/mmc$-Ba	0	0	0	0	0	0	0	0	0
$P6/mmm$-BaH_2	-0.681	-0.616	-0.526	-0.421	-0.305	-0.181	-0.050	0.0862	0.4488
$Imm2$-BaH_6	-0.343	-0.330	-0.318	-0.305	-0.293	-0.282	-0.270	-0.258	-0.229
$Cmmm$-BaH_{10}	-0.237	-0.232	-0.229	-0.228	-0.227	-0.228	-0.230	-0.232	-0.240
$Fm\bar{3}m$-BaH_{12}	0.237	0.242	0.245	0.245	0.245	0.244	0.242	0.239	0.231
$I4/mmm$-BaH_{12}	-0.161	-0.151	-0.142	-0.134	-0.126	-0.118	-0.111	-0.104	-0.086
$Immm$-BaH_{12}	-0.217	-0.198	-0.1768	-0.1544	-0.1311	-0.107	-0.082	-0.0570	0.0081
$Cmc2_1$-BaH_{12}	-0.166	-0.155	-0.144	-0.132	-0.121	-0.109	-0.098	-0.0862	-0.0564
$P2_1$-BaH_{12}	-0.236	-0.227	-0.216	-0.205	-0.194	-0.182	-0.170	-0.157	-0.124
$P1$-BaH_{12}	-0.205	-0.186	-0.164	-0.141	-0.119	-0.096	-0.072	-0.049	0.0098
$C2/c$-H	0	0	0	0	0	0	0	0	0

Table S11. Temperature dependence of the Gibbs free energy of formation (G_{form}, eV/atom), computed with the ZPE for various Ba–H phases at 150 GPa.

Temperature, K	100	300	500	700	900	1100	1300	1500	2000
$P6_3/mmc$-Ba	0	0	0	0	0	0	0	0	0
$P6/mmm$-BaH_2	-0.674	-0.615	-0.532	-0.434	-0.325	-0.207	-0.083	0.0467	0.3932
$Imm2$-BaH_6	-0.347	-0.335	-0.325	-0.317	-0.309	-0.302	-0.296	-0.290	-0.276
$Cmmm$-BaH_{10}	-0.264	-0.253	-0.238	-0.221	-0.203	-0.184	-0.163	-0.142	-0.085
$Fm\bar{3}m$-BaH_{12}	0.223	0.227	0.231	0.234	0.236	0.239	0.242	0.244	0.251
$I4/mmm$-BaH_{12}	-0.181	-0.173	-0.166	-0.159	-0.155	-0.145	-0.138	-0.131	-0.114
$Immm$-BaH_{12}	-0.228	-0.215	-0.200	-0.185	-0.169	-0.153	-0.136	-0.119	-0.076
$Cmc2_1$-BaH_{12}	-0.182	-0.175	-0.167	-0.1602	-0.152	-0.1436	-0.1349	-0.1259	-0.1027
$P2_1$-BaH_{12}	-0.228	-0.217	-0.207	-0.199	-0.191	-0.183	-0.176	-0.169	-0.154
$P1$-BaH_{12}	-0.222	-0.213	-0.205	-0.198	-0.192	-0.186	-0.181	-0.176	-0.164
$C2/c$-H	0	0	0	0	0	0	0	0	0

Table S12. Temperature dependence of the Gibbs free energy of formation (G_{form}, eV/atom), computed with the ZPE for various Ba–H phases at 200 GPa.

Temperature, K	100	300	500	700	900	1100	1300	1500	2000
$P6_3/mmc$-Ba	0	0	0	0	0	0	0	0	0
$P6/mmm$-BaH_2	-0.821	-0.763	-0.680	-0.581	-0.472	-0.355	-0.230	-0.099	0.2480
$Imm2$-BaH_6	-0.411	-0.406	-0.405	-0.406	-0.409	-0.414	-0.419	-0.425	-0.444
$Cmmm$-BaH_{10}	-0.317	-0.310	-0.301	-0.291	-0.281	-0.270	-0.258	-0.246	-0.214
$Fm\bar{3}m$-BaH_{12}	0.192	0.194	0.195	0.197	0.198	0.200	0.203	0.205	0.213
$I4/mmm$-BaH_{12}	-0.222	-0.216	-0.208	-0.202	-0.194	-0.186	-0.178	-0.171	-0.150
$Immm$-BaH_{12}	-0.271	-0.2640	-0.2574	-0.2508	-0.2442	-0.2375	-0.230	-0.2238	-0.2064
$Cmc2_1$-BaH_{12}	-0.229	-0.225	-0.2202	-0.2154	-0.2107	-0.2061	-0.2016	-0.1971	-0.1861
$P2_1$-BaH_{12}	-0.264	-0.254	-0.243	-0.232	-0.220	-0.209	-0.198	-0.186	-0.157
$P1$-BaH_{12}	-0.259	-0.248	-0.235	-0.221	-0.207	-0.191	-0.175	-0.159	-0.116
$C2/c$-H	0	0	0	0	0	0	0	0	0

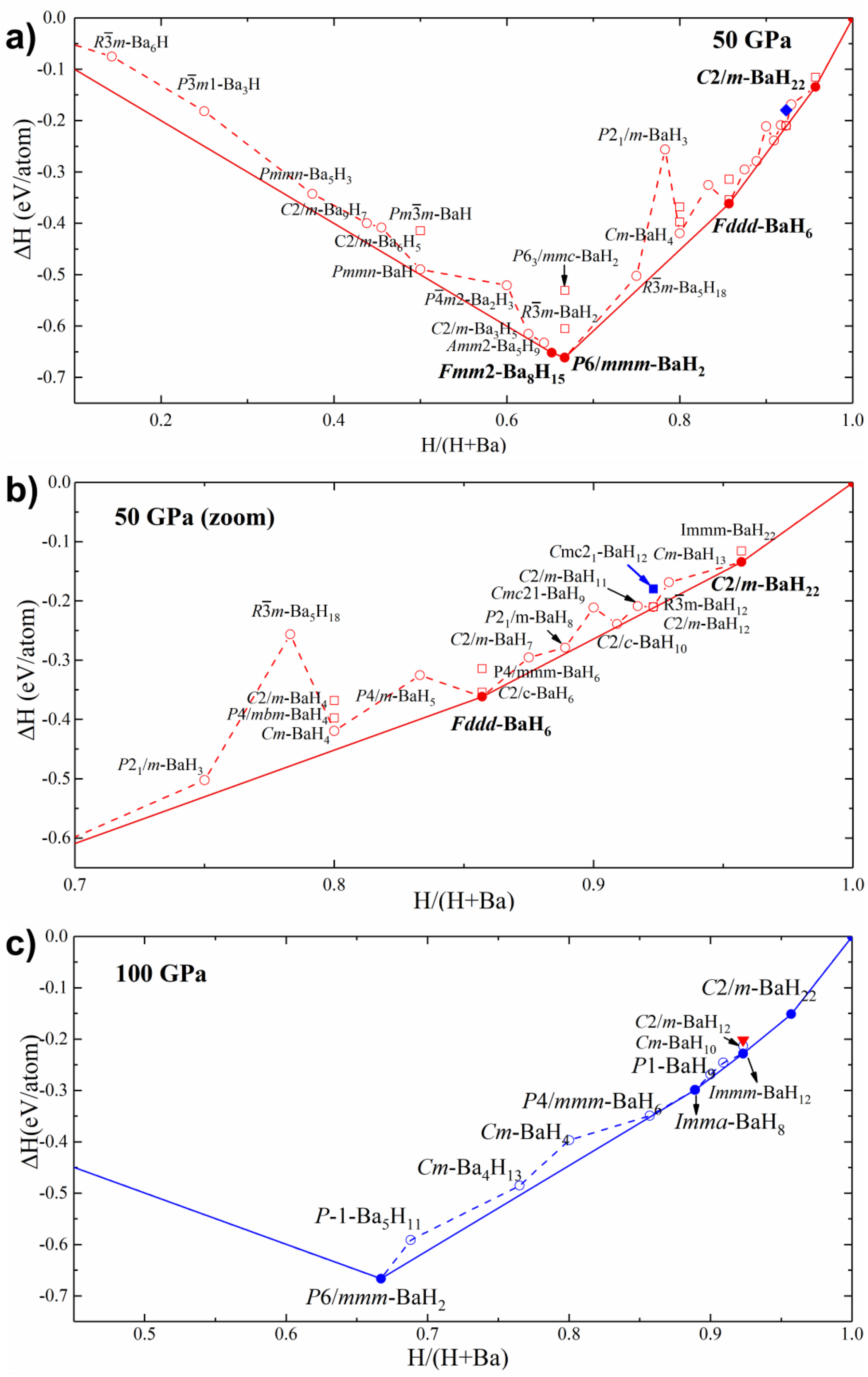

Fig. S2. Convex hull diagrams for the Ba–H system at (a, b) 50 GPa and (c) 100 GPa calculated using USPEX without the ZPE at 0 K. Many phases can be stable and may appear in experiments. At 50 GPa, the stable phases are the known *P6/mmm*-BaH_2, *Fddd*-BaH_6, and the higher molecular hydride *C2/m*-BaH_{22}.

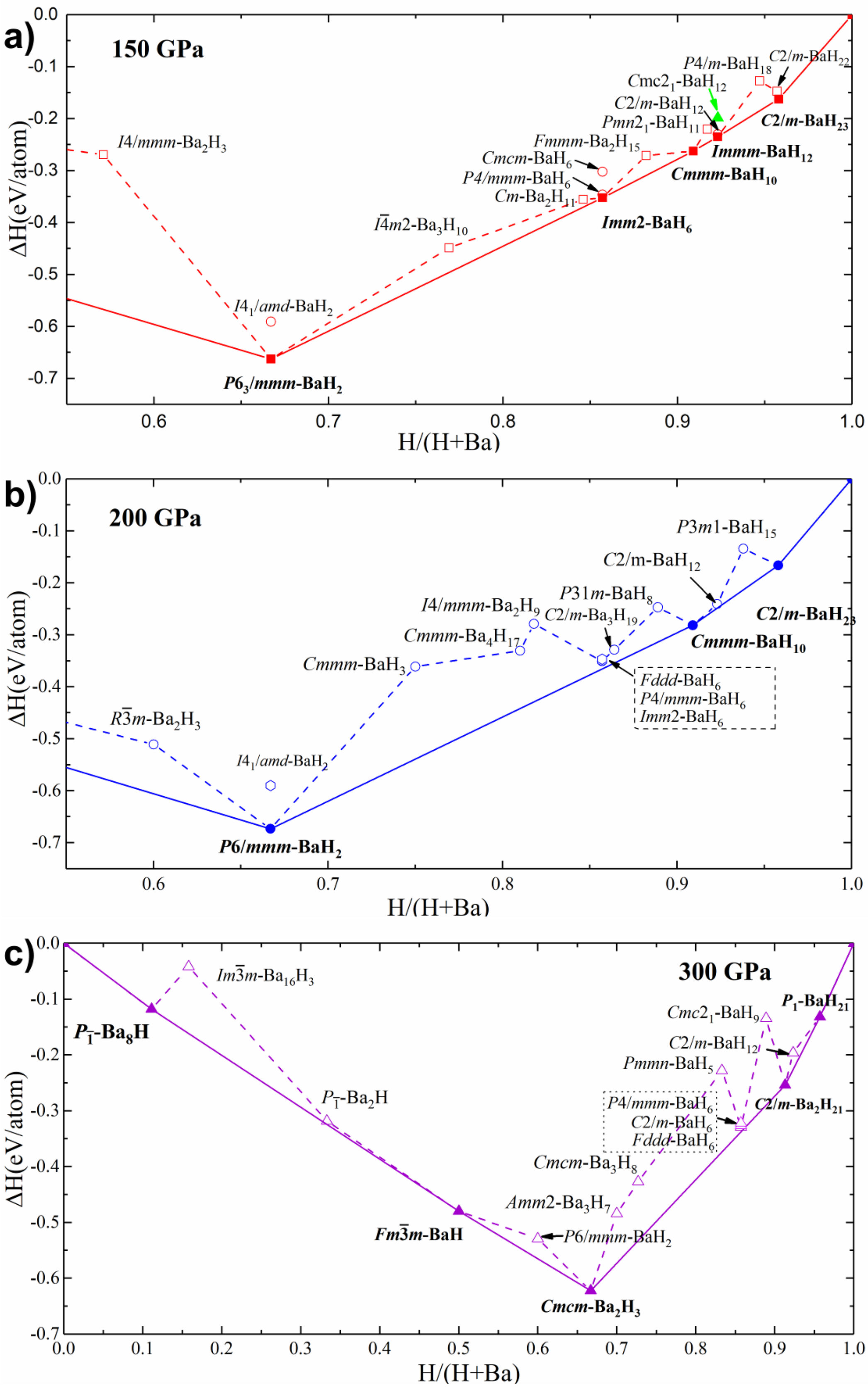

Fig. S3. Complex convex hull diagrams for the Ba–H system at (a) 150 GPa, (b) 200 GPa, and (c) 300 GPa calculated using USPEX without the ZPE at 0 K. We predict the absence of highly symmetric barium superhydrides and the stability of stoichiometric BaH_2, BaH_6, BaH_{10}, BaH_{12}, and higher hydride BaH_{21-23} in the pressure range of 50–300 GPa.

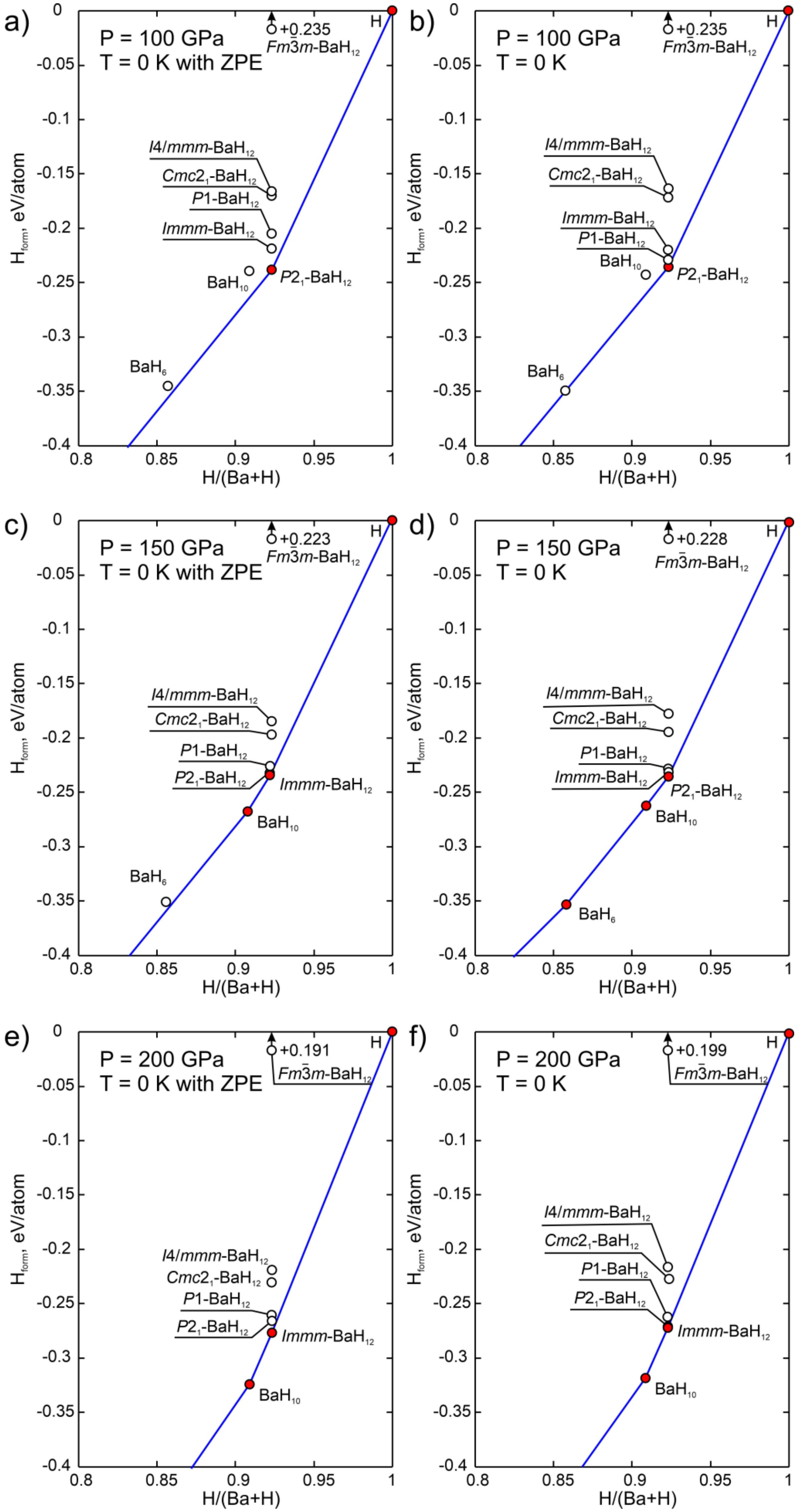

Fig. S4. Comparison of Ba-H convex hulls at 100-200 GPa with and without accounting of ZPE at 0 K.

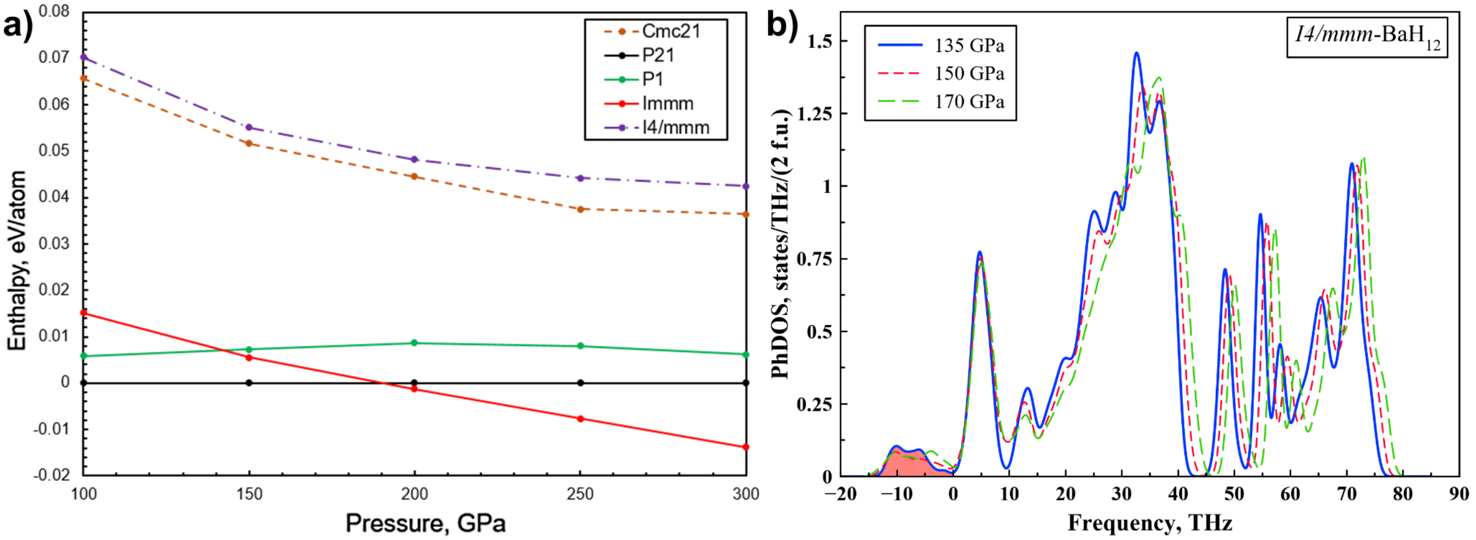

Fig. S5. (a) Enthalpy-pressure diagram and predicted phase transition $P2_1$-BaH_{12} → *Immm*-BaH_{12} at ~190 GPa (0 K). (b) Phonon density of states for *I*4/*mmm*-BaH_{12} at 135 (blue line), 150 (red dashed line) and 170 GPa (green dashed line).

Le Bail Refinements and XRD Data

Table S13. Peaks from cell #B1.

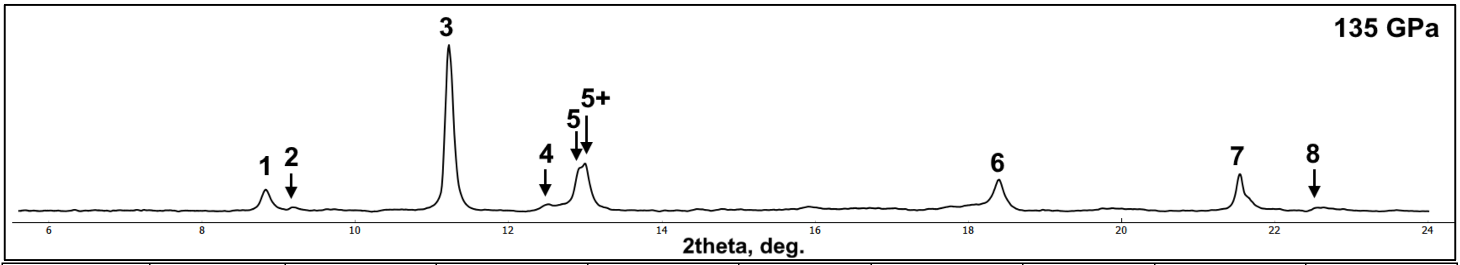

Pressure, GPa	Peak 1, 2θ	Peak 2, 2θ	Peak 3, 2θ	Peak 4, 2θ	Peak 5, 2θ	Peak 5+, 2θ	Peak 6, 2θ	Peak 7, 2θ	Peak 8, 2θ
160	8.982	9.45	11.403	12.723	13.136	-	18.629	21.906	22.909
154	8.96	9.442	11.389	12.695	13.115	-	18.617	21.868	22.88
145	8.895	9.276	11.312	12.599	13.027	-	18.521	21.718	22.723
135	8.837	9.2	11.238	12.515	12.927	12.993	18.401	21.55	22.561
126	8.748	9.139	11.176	12.374	12.812	12.926	18.311	21.512	22.453
119	8.715	-	11.125	-	12.757	12.876	18.263	-	-

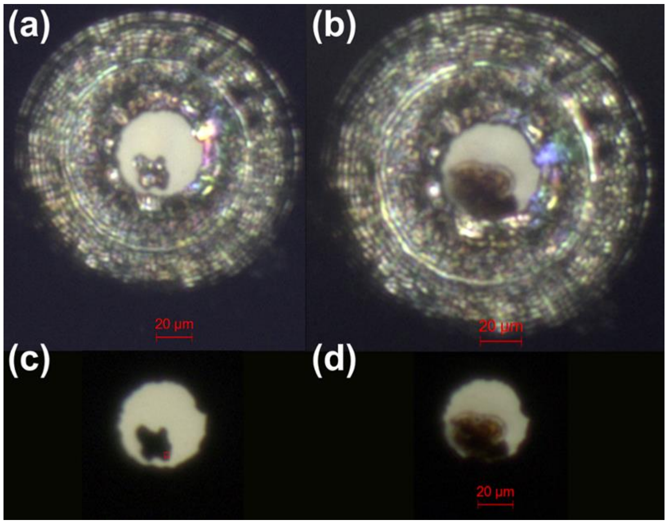

Fig. S6. Samples and culet of cell #B3 heated at 90 GPa: (a) before heating, in reflected light; (b) after heating, in reflected light; (c) before heating, in transmitted light; (d) after heating, in transmitted light. The presence of a semitransparent border may indicate the formation of nonmetallic molecular hydrides BaH_x and byproducts such as salts with NH_3BH_3 derivatives (e.g., $Ba(AB)_2$).

Table S14. Experimental cell parameters of the refined *P6/mmm*-BaH_2 and *P6₃/mmc*-Ba structures in cell #B0 (Fig. S4 and S5). Volumes are given per Ba atom.

Compound	Pressure, GPa	*a*, Å	*b*, Å	*c*, Å	*V*, Å^3	V_{DFT}, Å^3 *
$P6_3/mmc$-Ba	173	2.89(9)	2.89(9)	4.92(9)	17.9(4)	16.28
P6/mmm-BaH_2	173	2.822(3)	2.822(3)	2.717(8)	18.75	1
P6/mmm-BaH_2	154	2.860(6)	2.860(6)	2.755(8)	19.53	2
P6/mmm-BaH_2	130	2.925(8)	2.925(8)	2.795(1)	20.72	21.19

* Deviation from the experiment may be due to irregular distribution of pressure in the sample and the nonstoichiometric content of hydrogen in *P6/mmm*-BaH_{2-x} (x = 0–0.5).

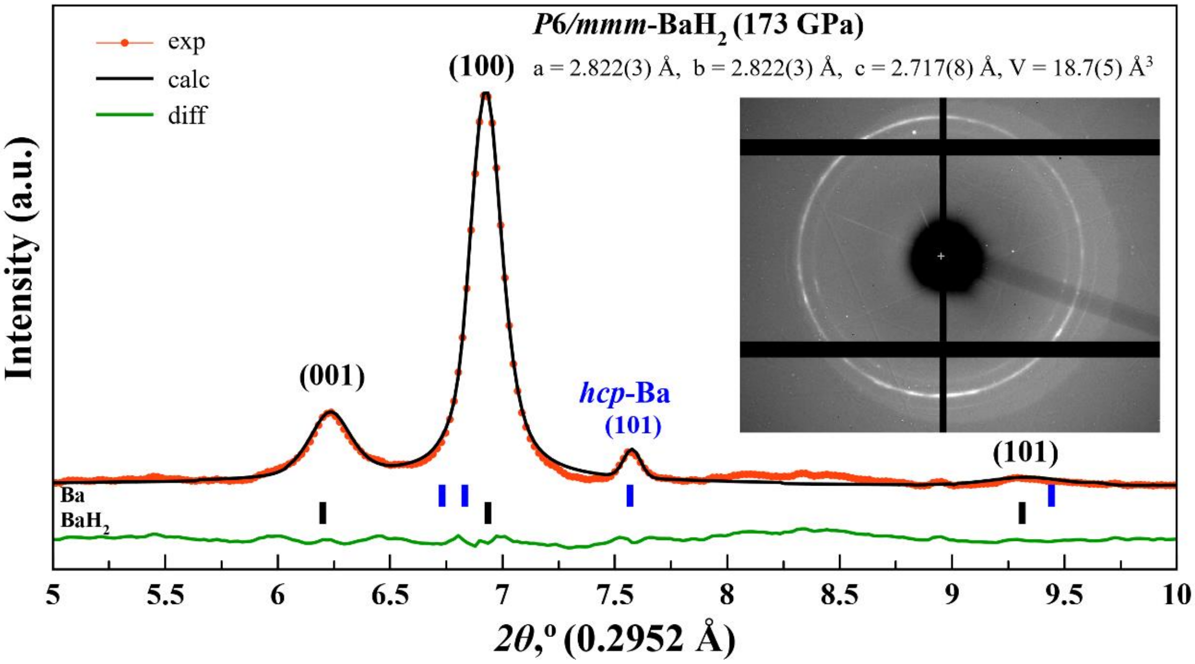

Fig. S7. Le Bail refinement of *P*6/*mmm*-BaH_2 and, possibly, *P*6_3/*mmc*-Ba, and the experimental XRD pattern at 173 GPa (cell #B0). The experimental data, model fit for the structure, and residues are shown in red, black, and green, respectively. The range of recording was limited to ~9º.

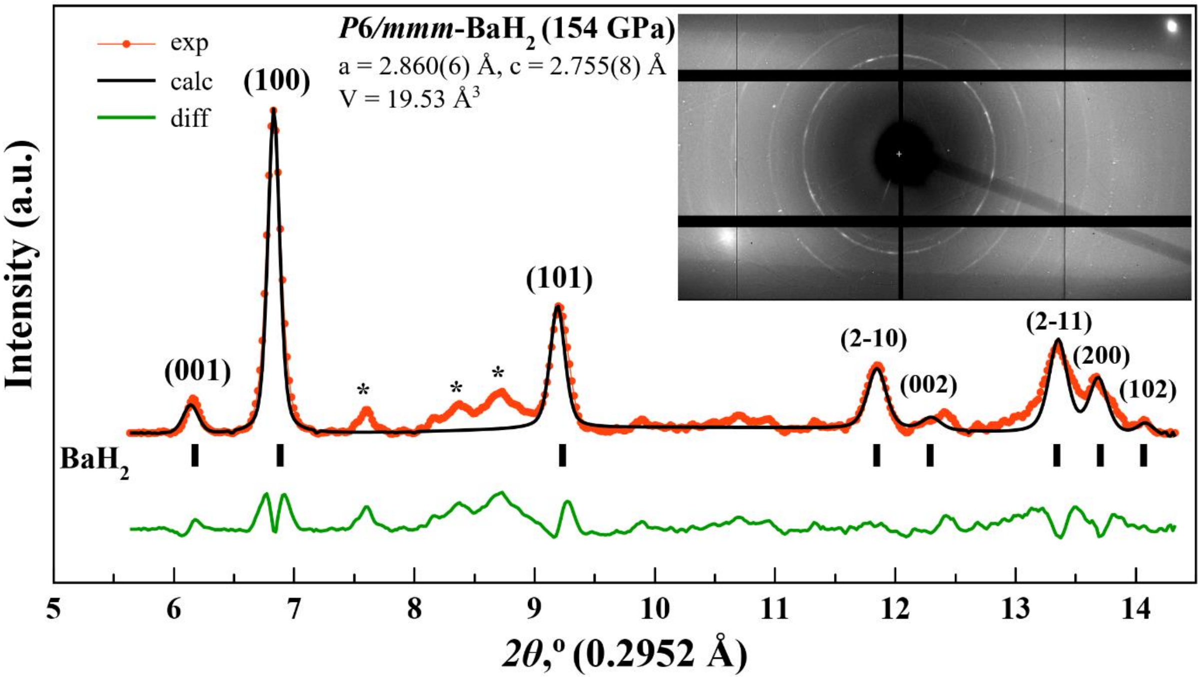

Fig. S8. Le Bail refinement of *P*6/*mmm*-BaH_2 and the experimental XRD pattern at 154 GPa (cell #B0). The experimental data, model fit for the structure, and residues are shown in red, black, and green, respectively. Unidentified reflections are indicated by asterisks.

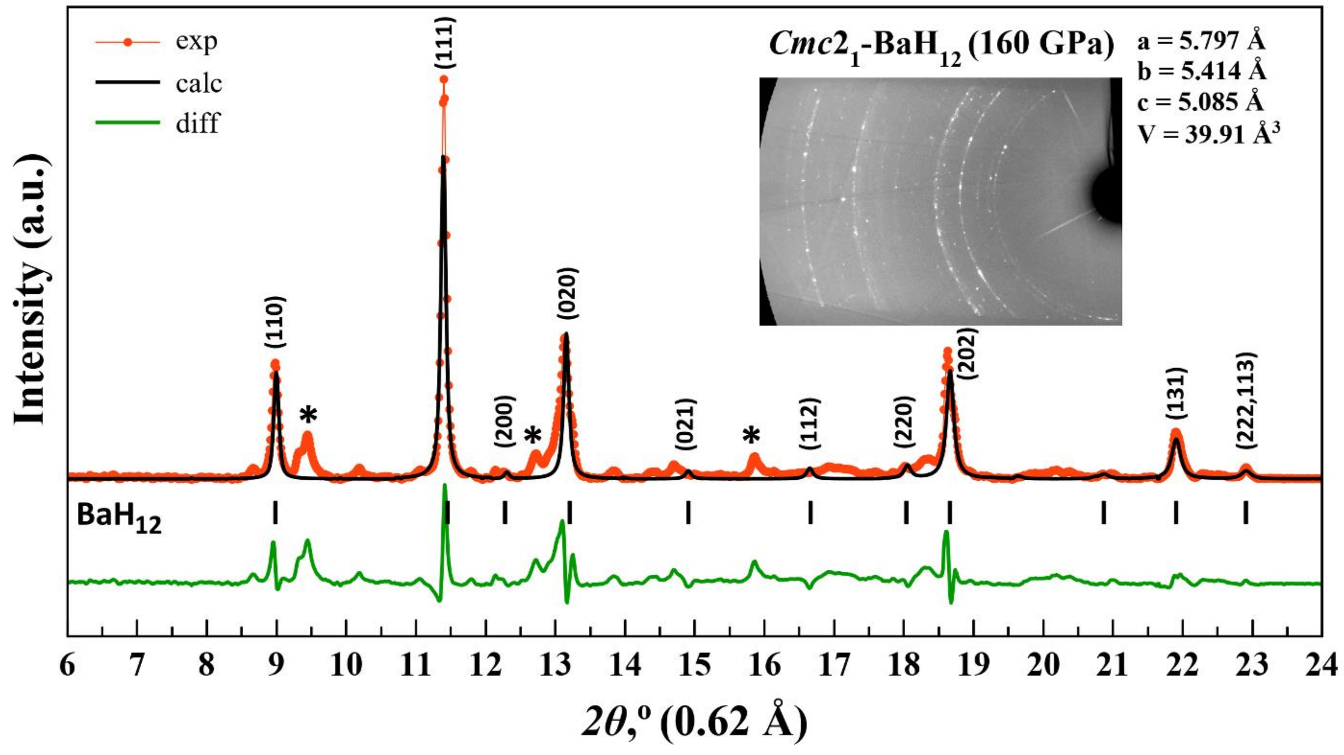

Fig. S9. Le Bail refinement of distorted *Cmc*2_1-BaH_{12} and the experimental XRD pattern at 160 GPa, including the reflection at 9° (cell #B1). The experimental data, model fit for the structure, and residues are shown in red, black, and green, respectively. Unidentified reflections are indicated by asterisks.

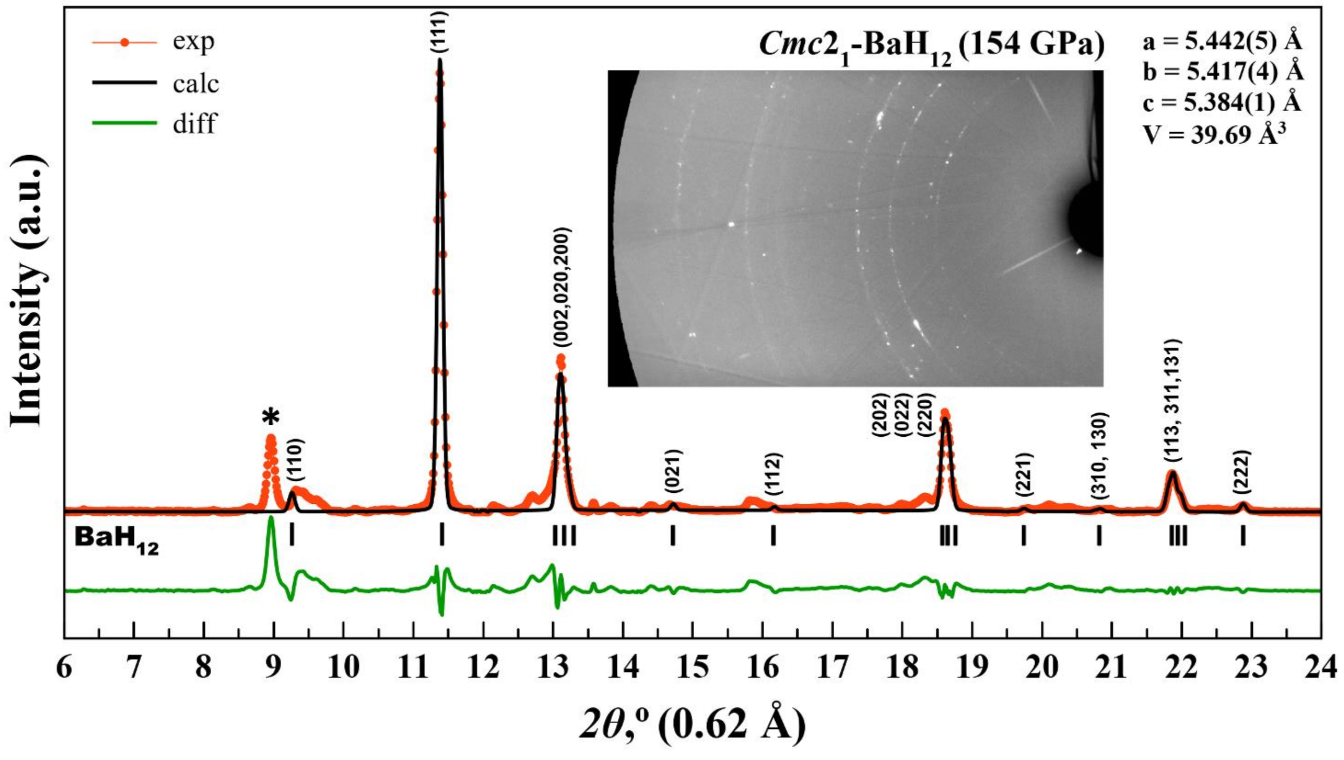

Fig. S10. Le Bail refinement of distorted *Cmc*2_1-BaH_{12} and the experimental XRD pattern at 154 GPa (cell #B1). The experimental data, model fit for the structure, and residues are shown in red, black, and green, respectively. Unidentified reflections are indicated by asterisks.

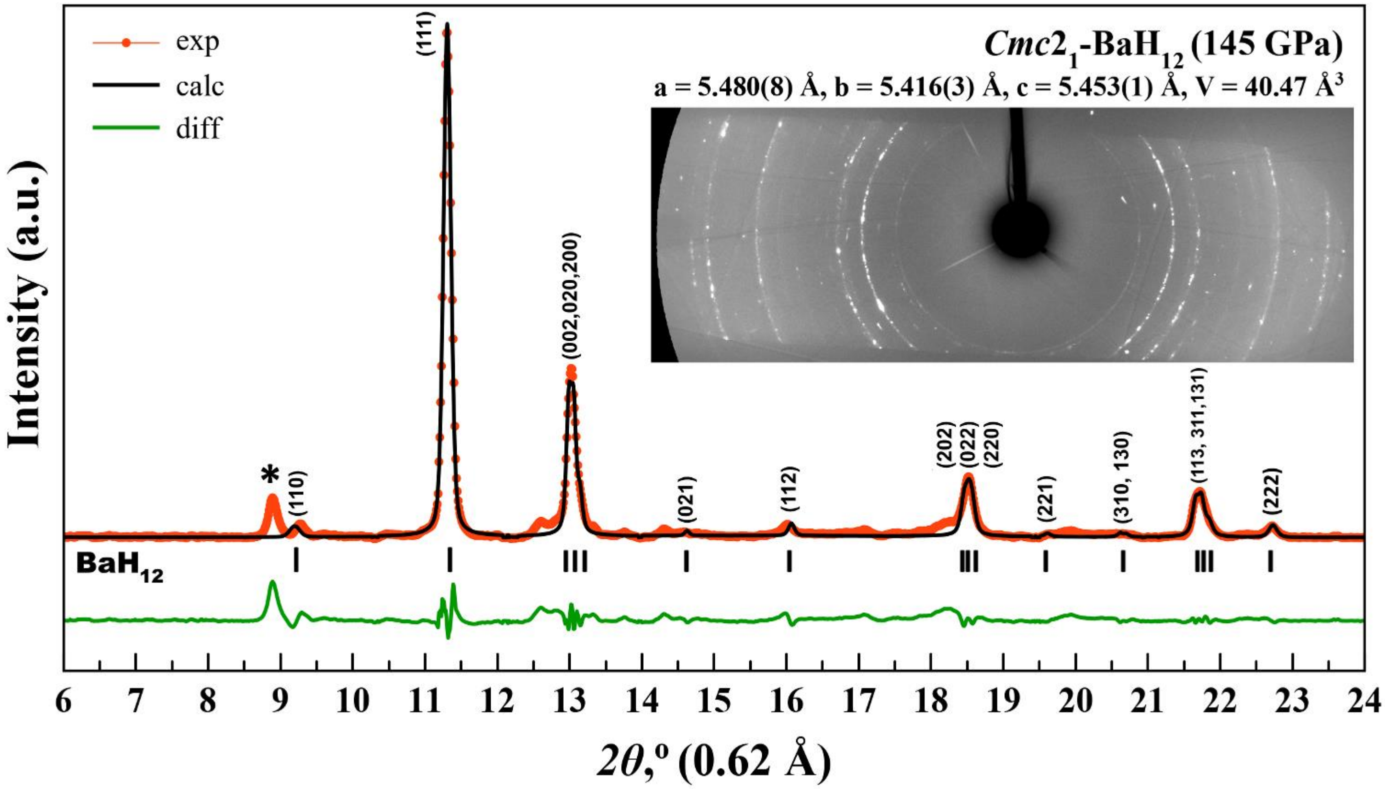

Fig. S11. Le Bail refinement of distorted *Cmc*2_1-BaH_{12} and the experimental XRD pattern at 145 GPa (cell #B1). The experimental data, model fit for the structure, and residues are shown in red, black, and green, respectively. Unidentified reflections are indicated by asterisks.

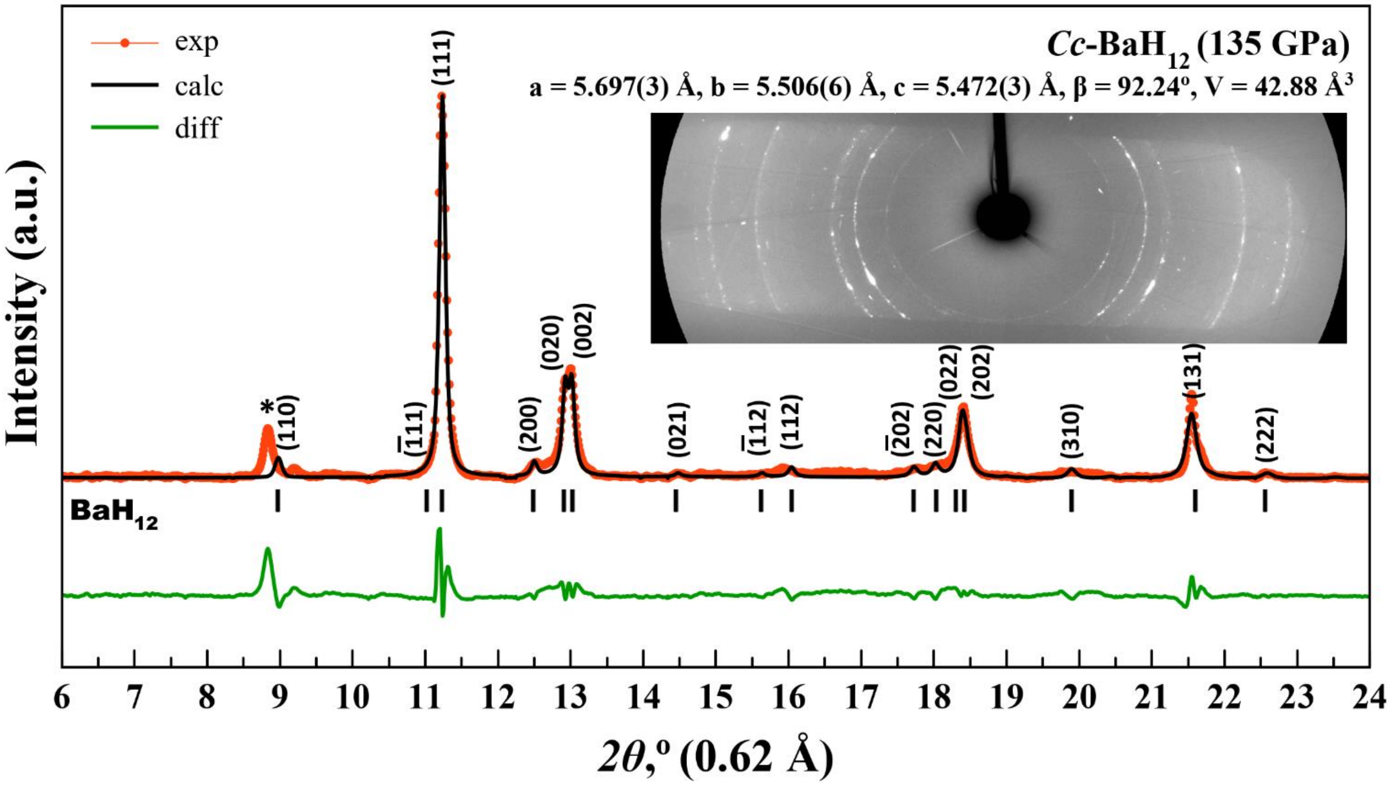

Fig. S12. Le Bail refinement of distorted *Cmc*2_1-BaH_{12} and the experimental XRD pattern at 135 GPa (cell #B1). The experimental data, model fit for the structure, and residues are shown in red, black, and green, respectively. Unidentified reflections are indicated by asterisks.

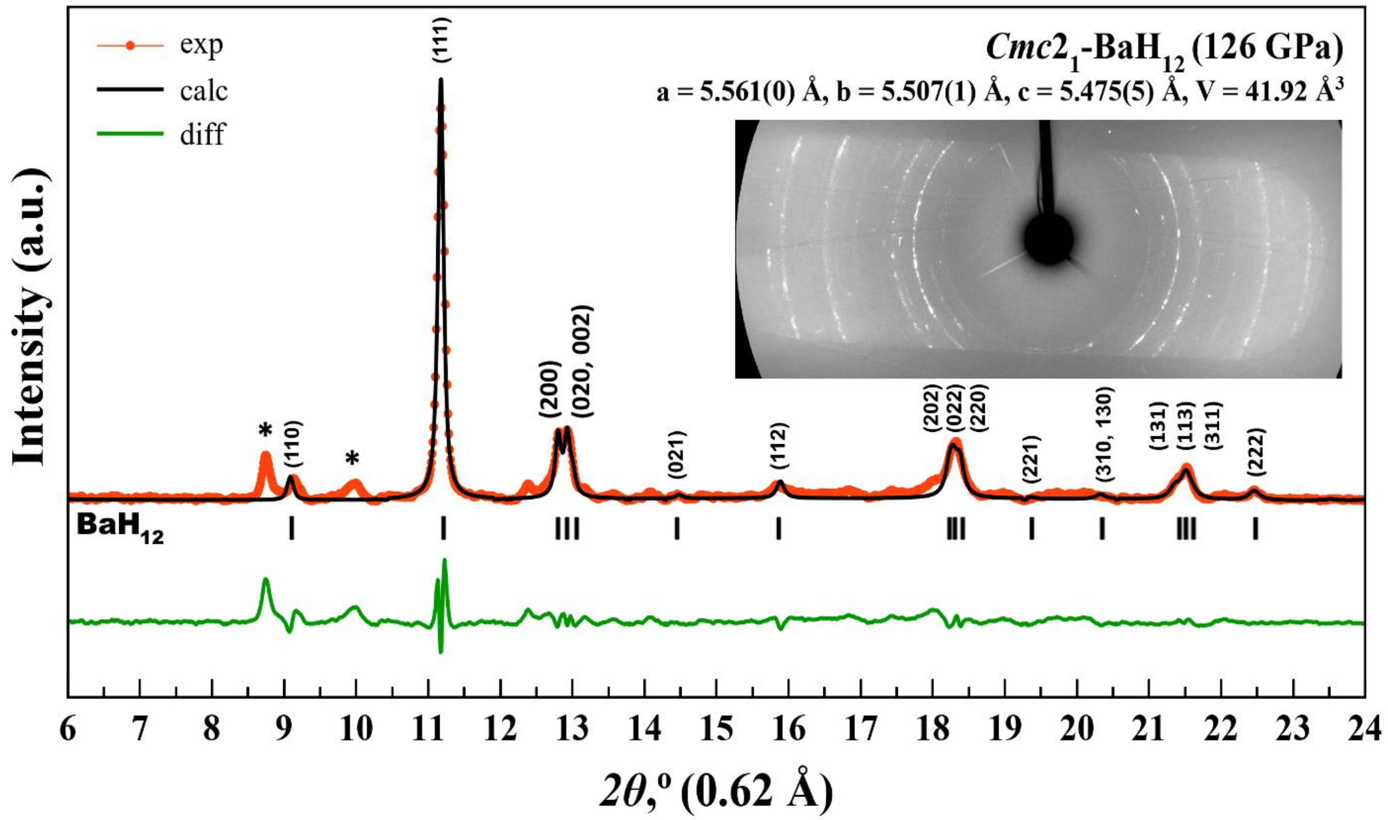

Fig. S13. Le Bail refinement of pseudocubic *Cmc*2_1-BaH_{12} and the experimental XRD pattern at 126 GPa (cell #B1). The experimental data, model fit for the structure, and residues are shown in red, black, and green, respectively. Unidentified reflections are indicated by asterisks.

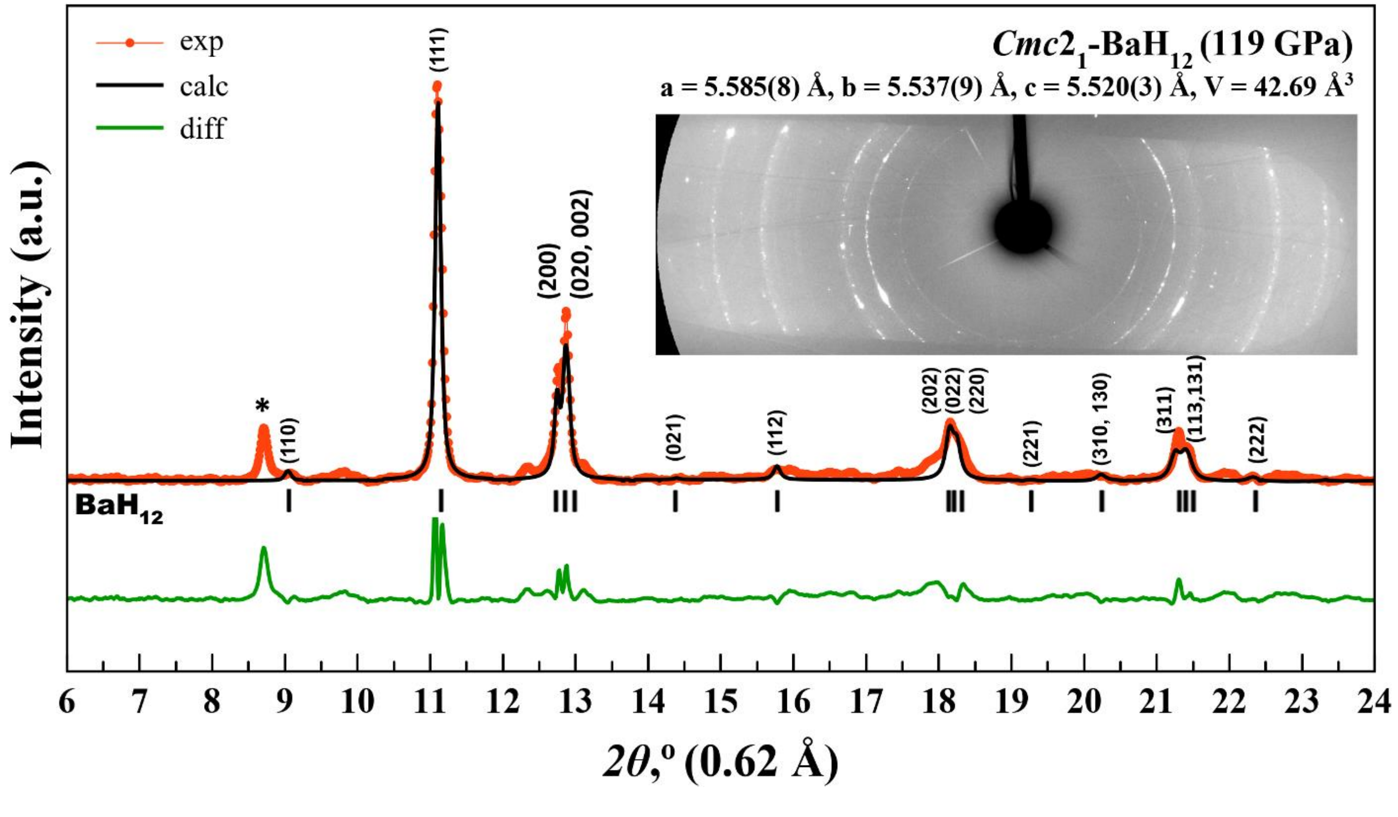

Fig. S14. Le Bail refinement of pseudocubic *Cmc*2_1-BaH_{12} and the experimental XRD pattern at 119 GPa (cell #B1). The experimental data, model fit for the structure, and residues are shown in red, black, and green, respectively. Unidentified reflections are indicated by asterisks.

Table S15. Experimental cell parameters of the refined pseudocubic *Cmc*2_1-BaH_{12} structure in different points of cell #B2 (see the map, Fig. S13–S16). Volumes are given per Ba atom.

Point #	*a*, Å	*b*, Å	*c*, Å	*V*, Å^3
01	5.500(1)	5.481(6)	5.539(3)	41.75
02	5.512(0)	5.543(2)	5.485(4)	41.90
03	5.516(5)	5.550(1)	5.490(0)	42.02
04	5.520(5)	5.552(8)	5.494(0)	42.10

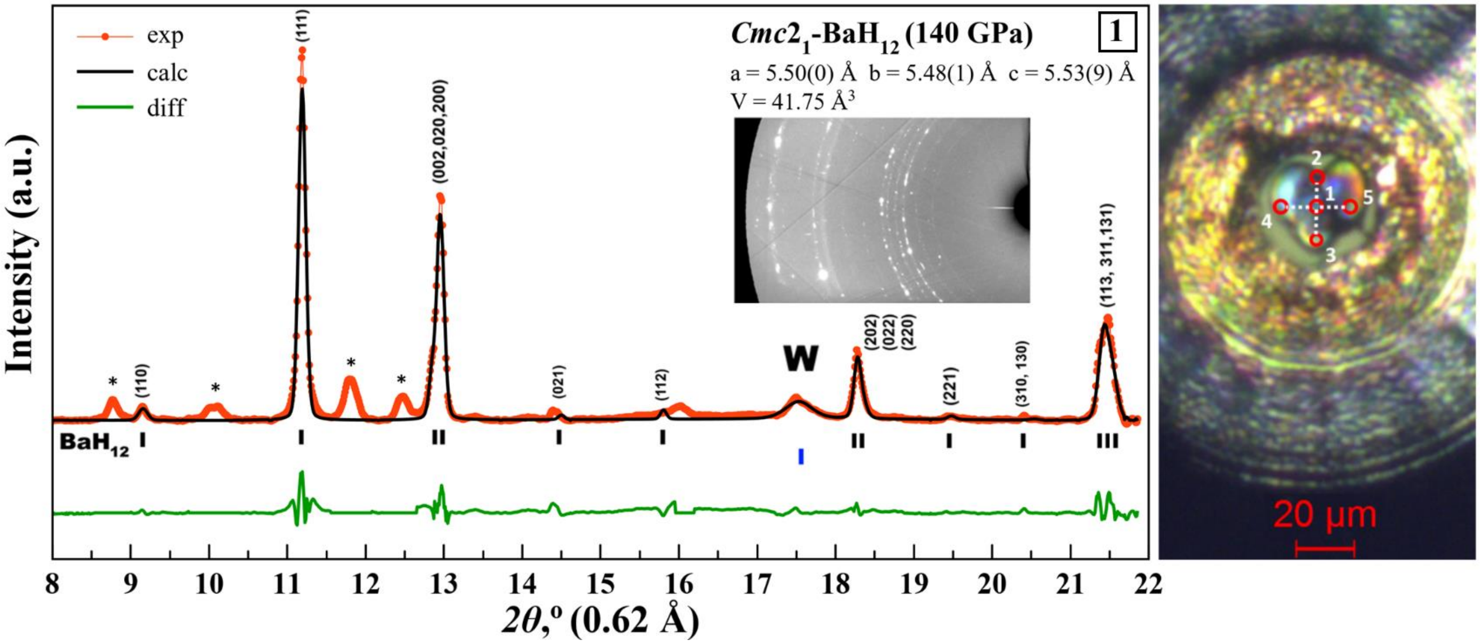

Fig. S15. Le Bail refinement of pseudocubic *Cmc*2_1-BaH_{12} and the experimental XRD pattern at ~140 GPa at point #1 (see the map, cell #B2). The experimental data, model fit for the structure, and residues are shown in red, black, and green, respectively. Unidentified reflections are indicated by asterisks.

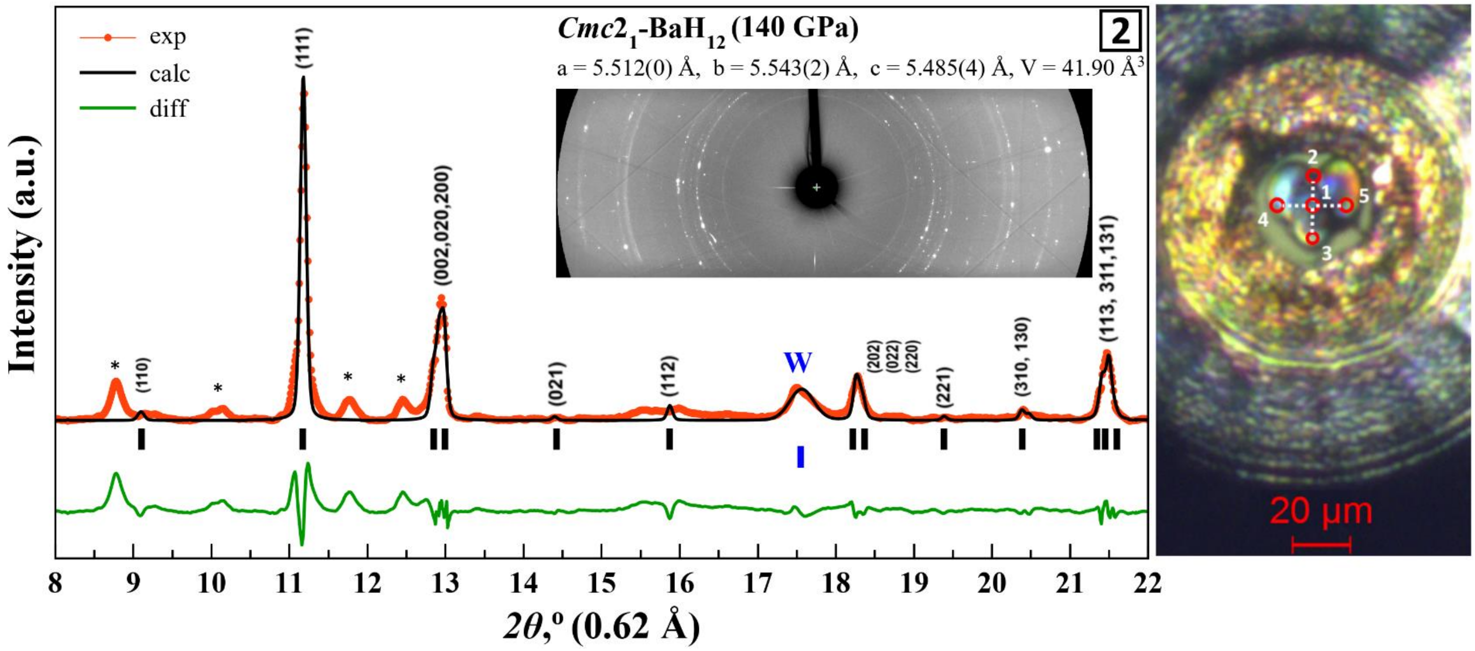

Fig. S16. Le Bail refinement of pseudocubic *Cmc*2_1-BaH_{12} and the experimental XRD pattern at ~140 GPa at point #2 (see the map, cell #B2). The experimental data, model fit for the structure, and residues are shown in red, black, and green, respectively. Unidentified reflections are indicated by asterisks.

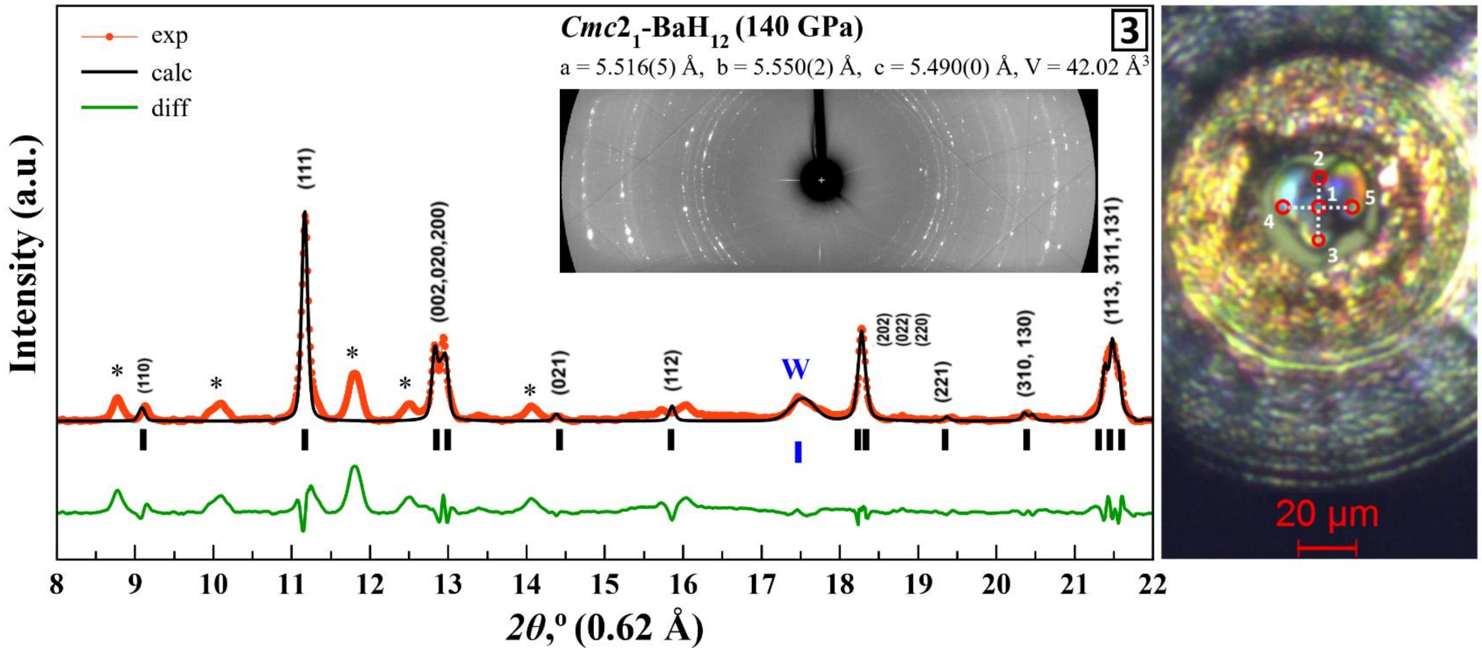

Fig. S17. Le Bail refinement of pseudo-cubic $Cmc2_1$-BaH_{12} and the experimental XRD pattern at ~140 GPa at point #3 (see the map, cell #B2). The experimental data, model fit for the structure, and residues are shown in red, black, and green, respectively. Unidentified reflections are indicated by asterisks.

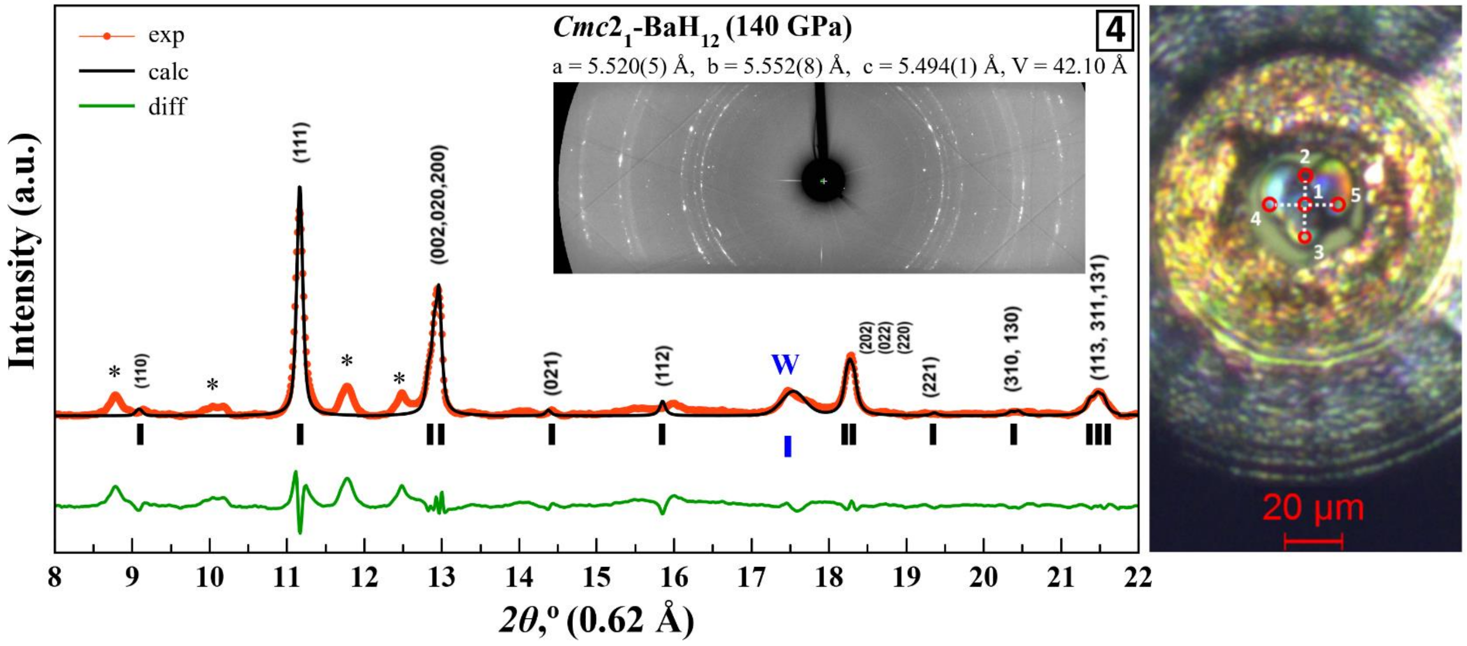

Fig. S18. Le Bail refinement of pseudocubic $Cmc2_1$-BaH_{12} and the experimental XRD pattern at ~140 GPa at point #4 (see the map, cell #B2). The experimental data, model fit for the structure, and residues are shown in red, black, and green, respectively. Unidentified reflections are indicated by asterisks.

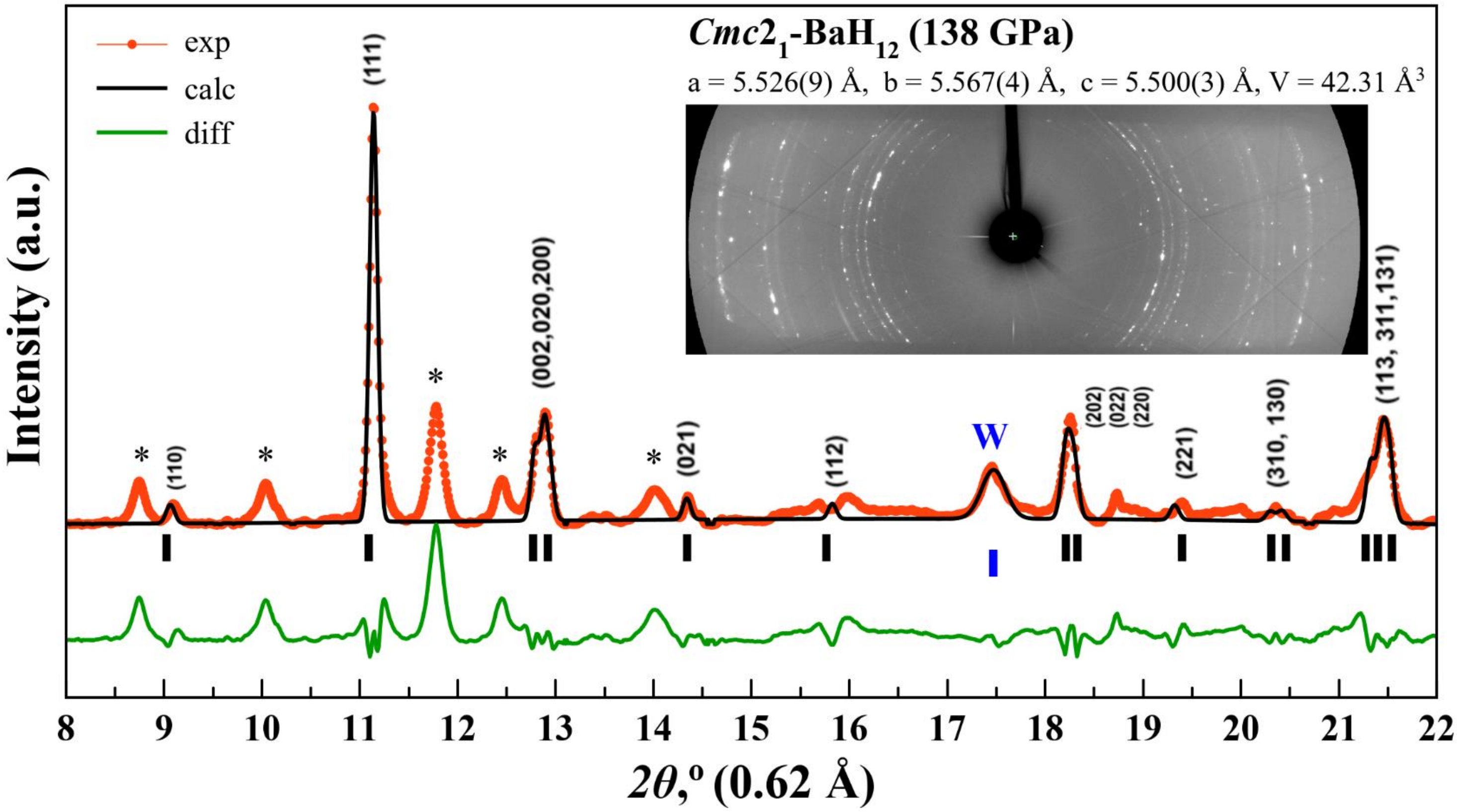

Fig. S19. Le Bail refinement of pseudocubic *Cmc*2_1-BaH_{12} and the experimental XRD pattern at 138 GPa (cell #B2). The experimental data, model fit for the structure, and residues are shown in red, black, and green, respectively. Unidentified reflections are indicated by asterisks.

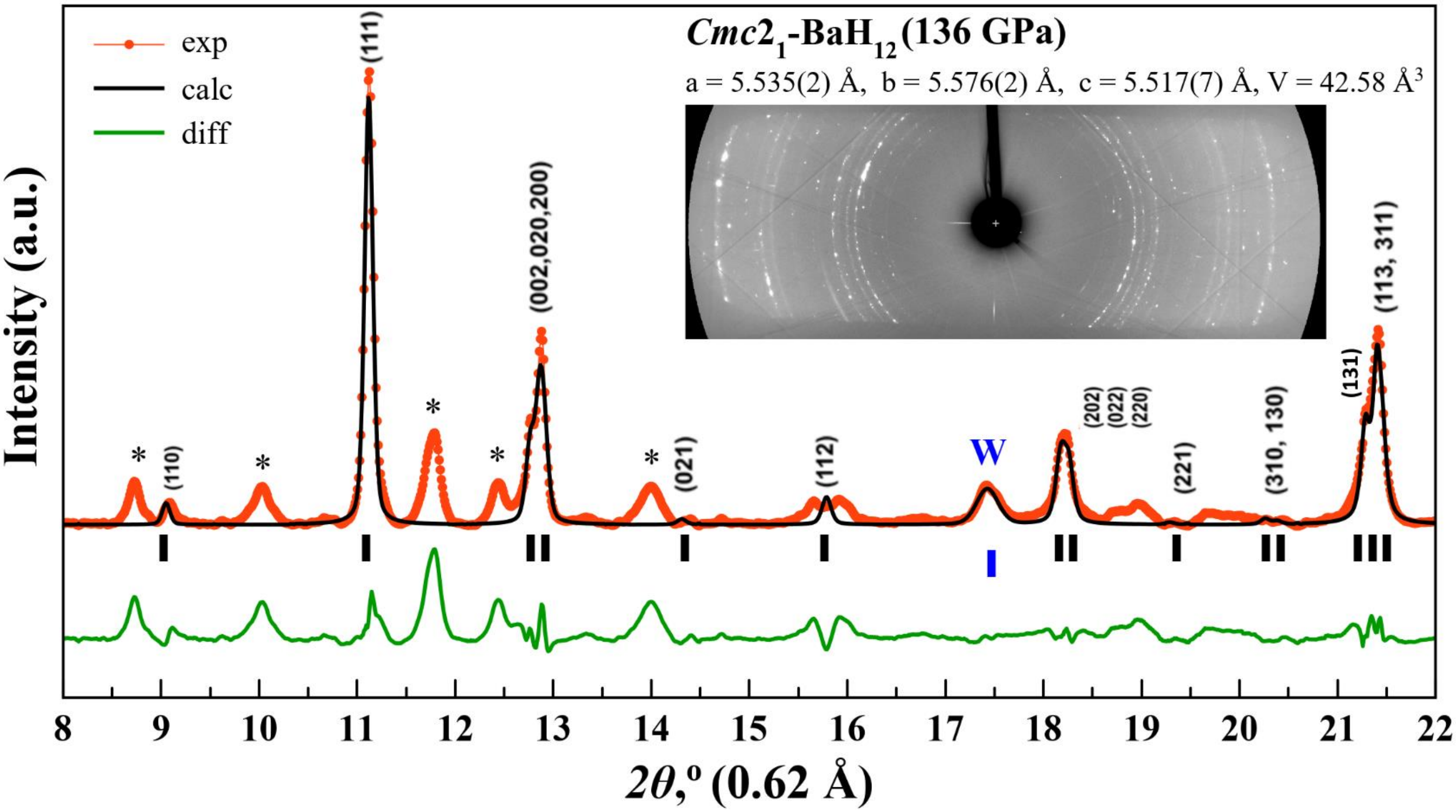

Fig. S20. Le Bail refinement of pseudocubic *Cmc*2_1-BaH_{12} and the experimental XRD pattern at 136 GPa (cell #B2). The experimental data, model fit for the structure, and residues are shown in red, black, and green, respectively. Unidentified reflections are indicated by asterisks.

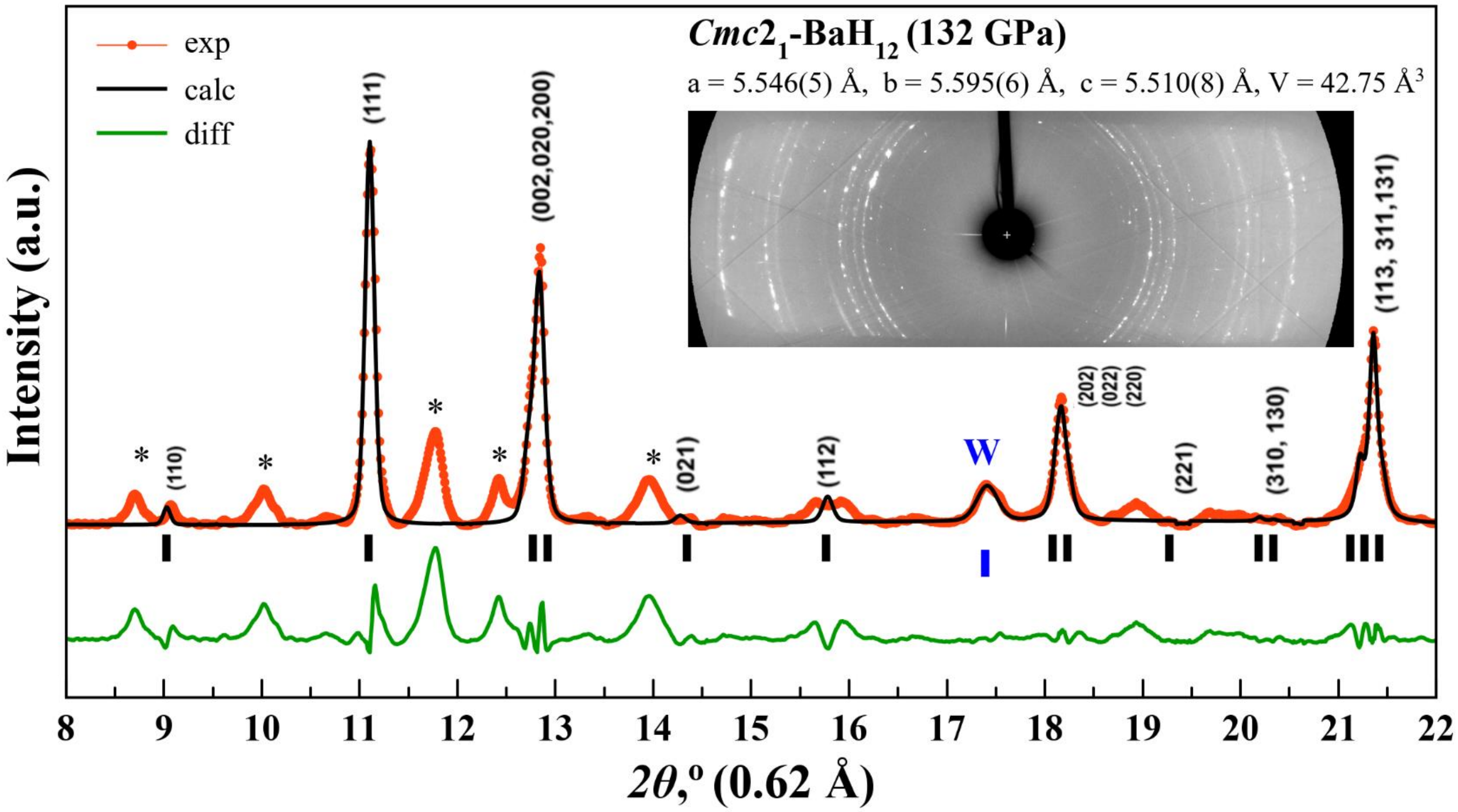

Fig. S21. Le Bail refinement of pseudocubic *Cmc*2₁-BaH₁₂ and the experimental XRD pattern at 132 GPa (cell #B2). The experimental data, model fit for the structure, and residues are shown in red, black, and green, respectively. Unidentified reflections are indicated by asterisks.

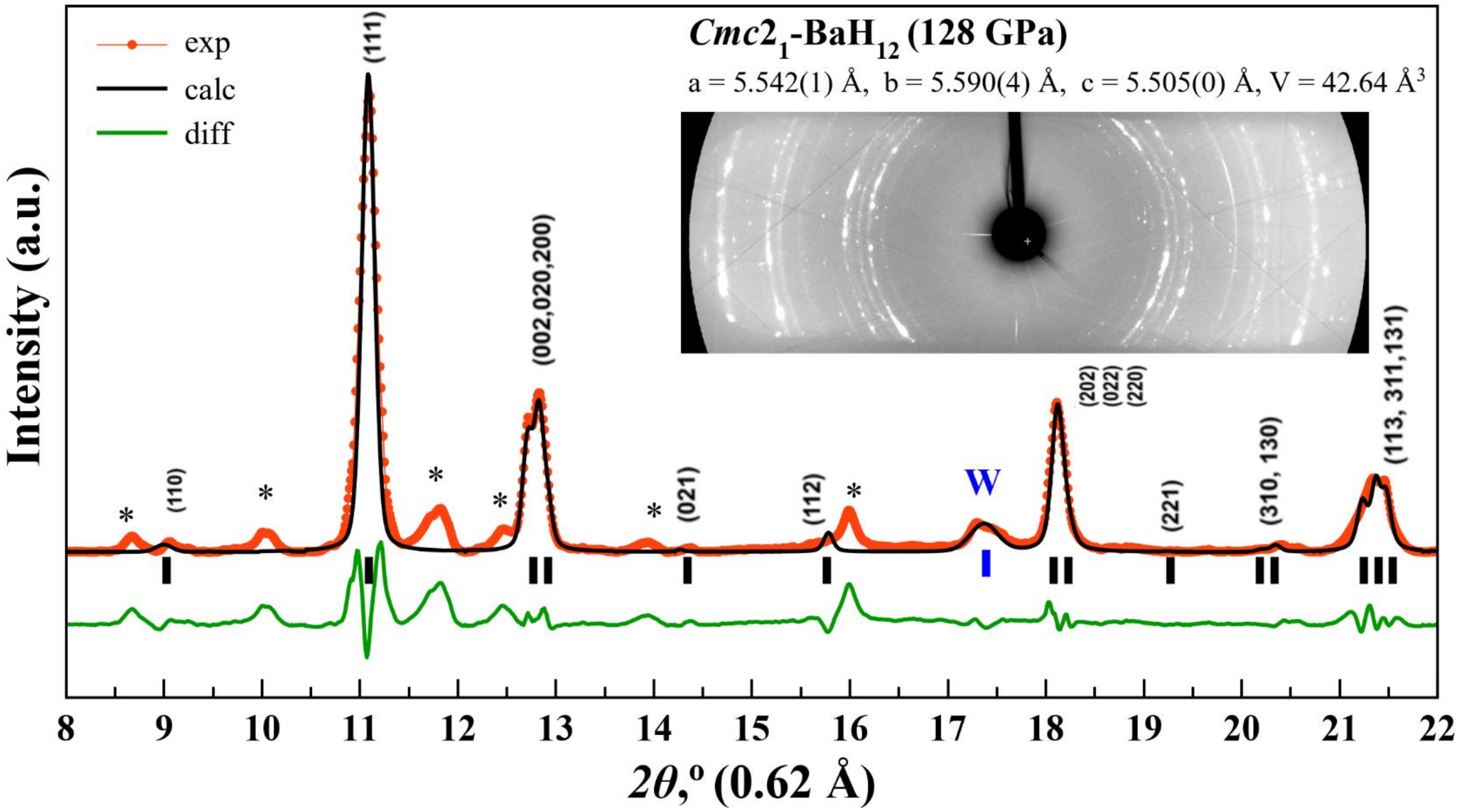

Fig. S22. Le Bail refinement of pseudocubic *Cmc*2₁-BaH₁₂ and the experimental XRD pattern at 128 GPa (cell #B2). The experimental data, model fit for the structure, and residues are shown in red, black, and green, respectively. Unidentified reflections are indicated by asterisks.

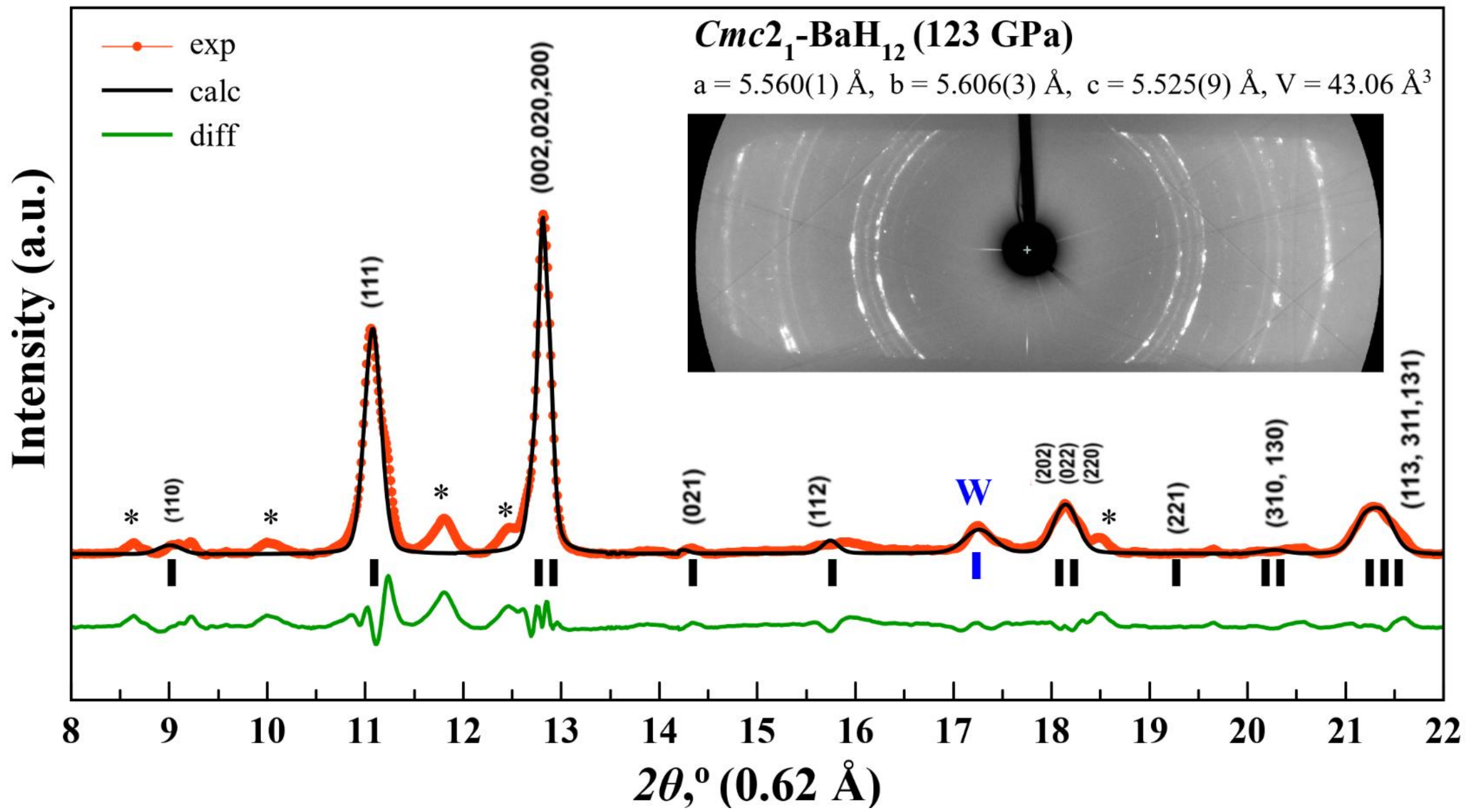

Fig. S23. Le Bail refinement of pseudocubic *Cmc*2_1-BaH_{12} and the experimental XRD pattern at 123 GPa (cell #B2). The experimental data, model fit for the structure, and residues are shown in red, black, and green, respectively. Unidentified reflections are indicated by asterisks.

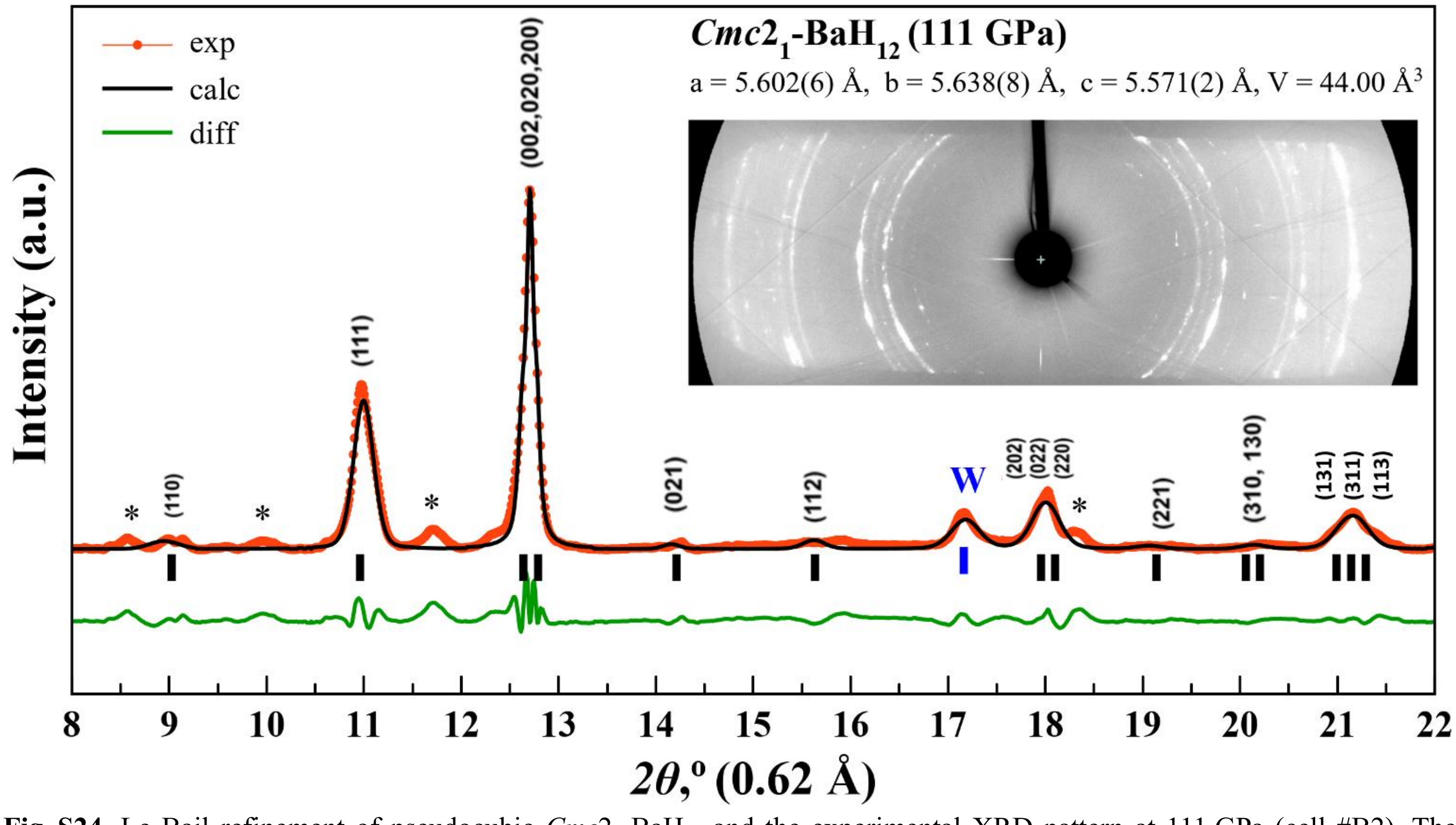

Fig. S24. Le Bail refinement of pseudocubic *Cmc*2_1-BaH_{12} and the experimental XRD pattern at 111 GPa (cell #B2). The experimental data, model fit for the structure, and residues are shown in red, black, and green, respectively. Unidentified reflections are indicated by asterisks.

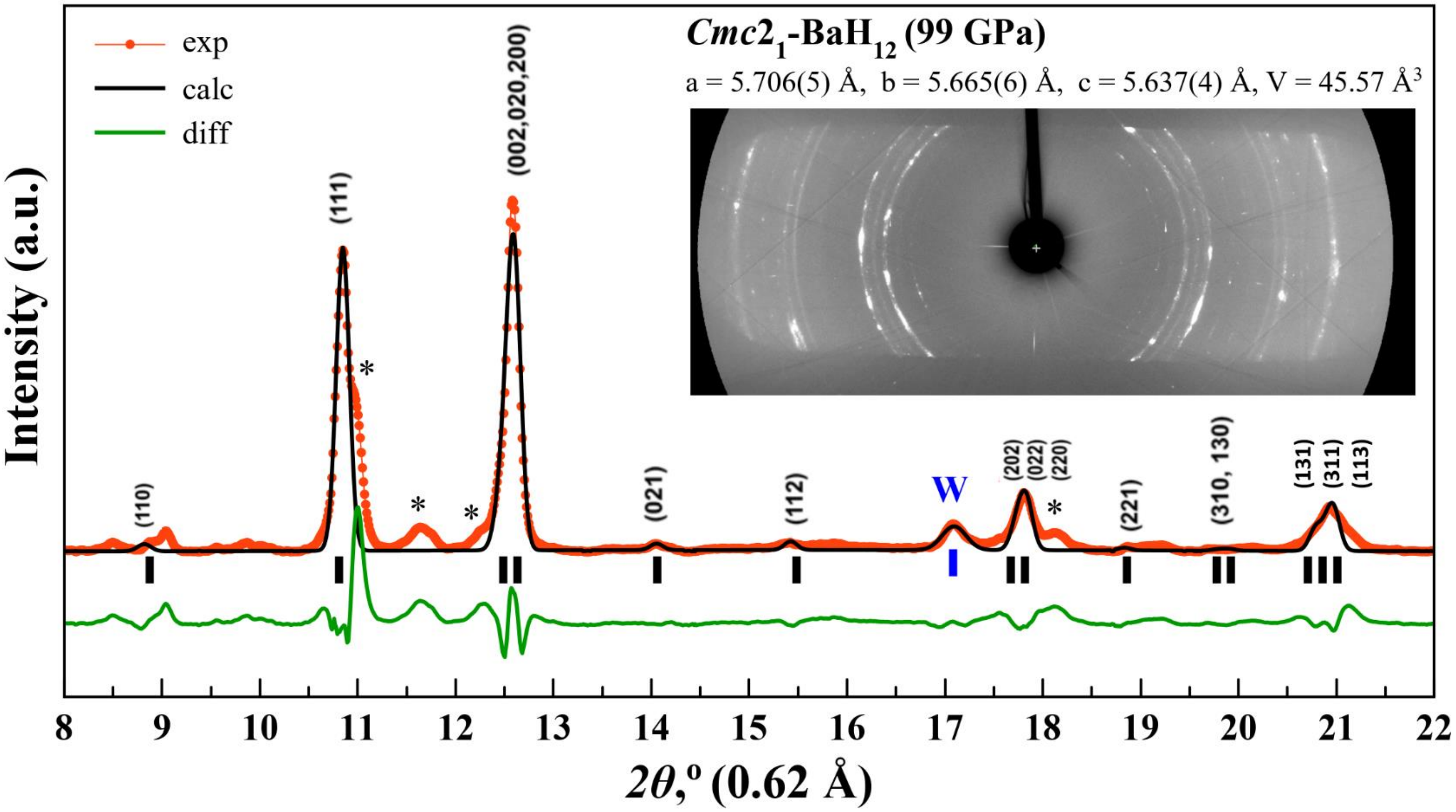

Fig. S25. Le Bail refinement of pseudocubic *Cmc*2_1-BaH_{12} and the experimental XRD pattern at 99 GPa (cell #B2). The experimental data, model fit for the structure, and residues are shown in red, black, and green, respectively. Unidentified reflections are indicated by asterisks.

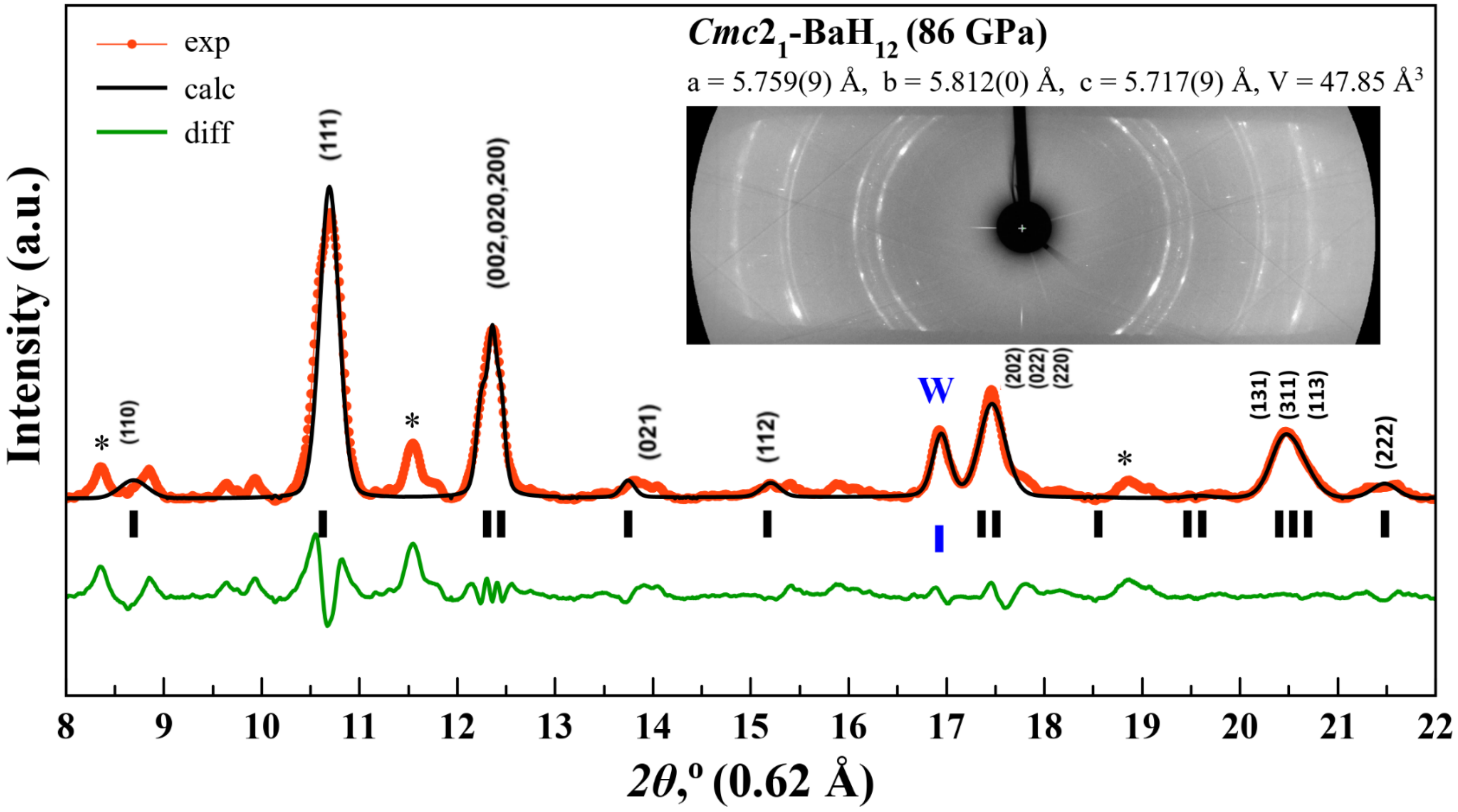

Fig. S26. Le Bail refinement of pseudocubic *Cmc*2_1-BaH_{12} and the experimental XRD pattern at 86 GPa (cell #B2). The experimental data, model fit for the structure, and residues are shown in red, black, and green, respectively. Unidentified reflections are indicated by asterisks.

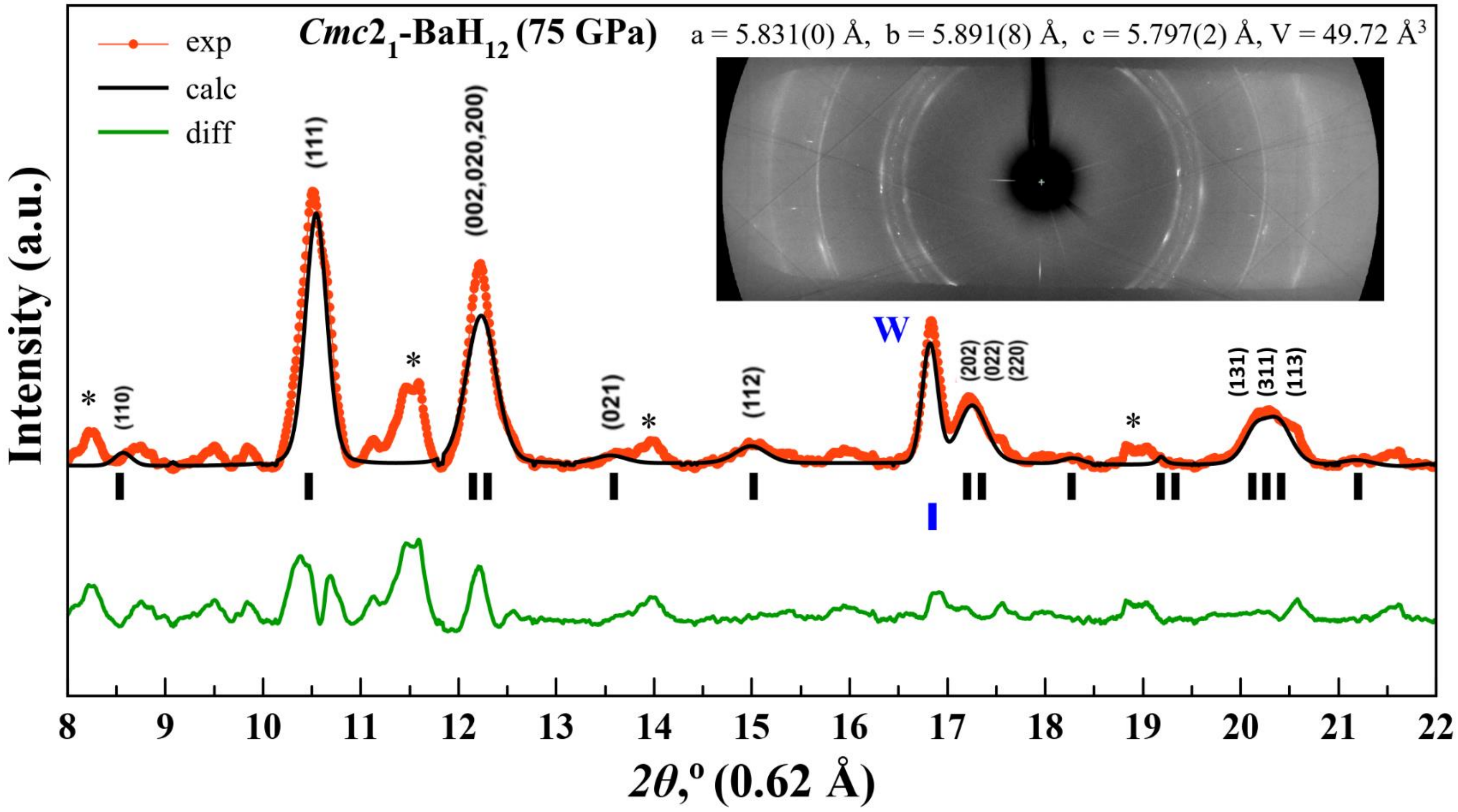

Fig. S27. Le Bail refinement of pseudocubic *Cmc*2_1-BaH_{12} and the experimental XRD pattern at 75 GPa (cell #B2). The experimental data, model fit for the structure, and residues are shown in red, black, and green, respectively. Unidentified reflections are indicated by asterisks.

Synthesis at 173 and 154 GPa: Side Products

The highest pressure we studied for the Ba–H system was reached in DAC #B0 with a culet size of 50 μm and Ba sample diameter ~17 μm (Fig. S27 and S29). As a result, an inhomogeneous mixture of several phases was obtained: *P6/mmm*-BaH_2 + *hcp*-Ba (Fig. S6 and S7), pseudocubic *Cmc*2₁-BaH_{12}, and possibly $P\bar{6}m2$-$BaH_{\sim 10}$ (Fig. S27 and S28). The obtained pattern is similar to the diffraction images from other diamond anvil cells, only byproducts are different. Unfortunately, after the pressure was reduced to 154 GPa, the diamond anvil cell spontaneously collapsed, therefore in this experiment we collected the information at only two pressure points.

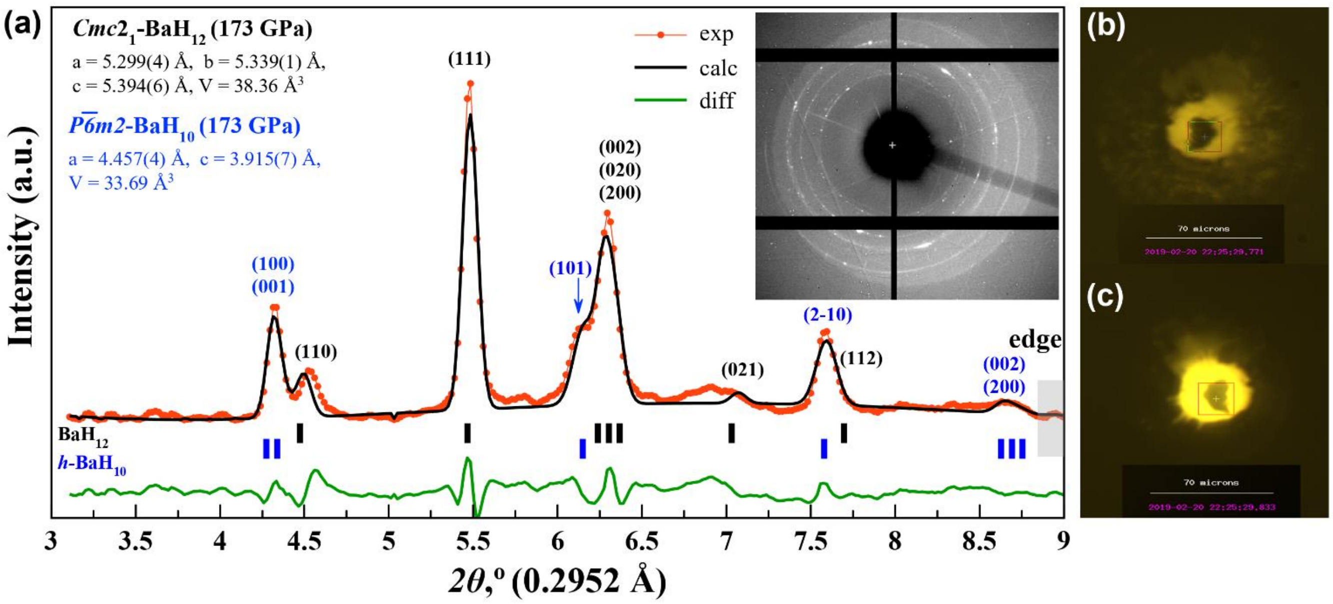

Fig. S28. (a) Experimental X-ray diffraction pattern and the Le Bail refinement of pseudocubic *Cmc*2₁-BaH_{12} and possible $P\bar{6}m2$-BaH_{10} from DAC #B0 at 173 GPa. The experimental data, fitted line, and residues are shown in red, black, and green, respectively. Edge: limitation of the diffraction angle due to the small size of the "WC seat" window. Culet of cell #B0 with a sample in (b) reflected and (c) transmitted light after the laser heating.

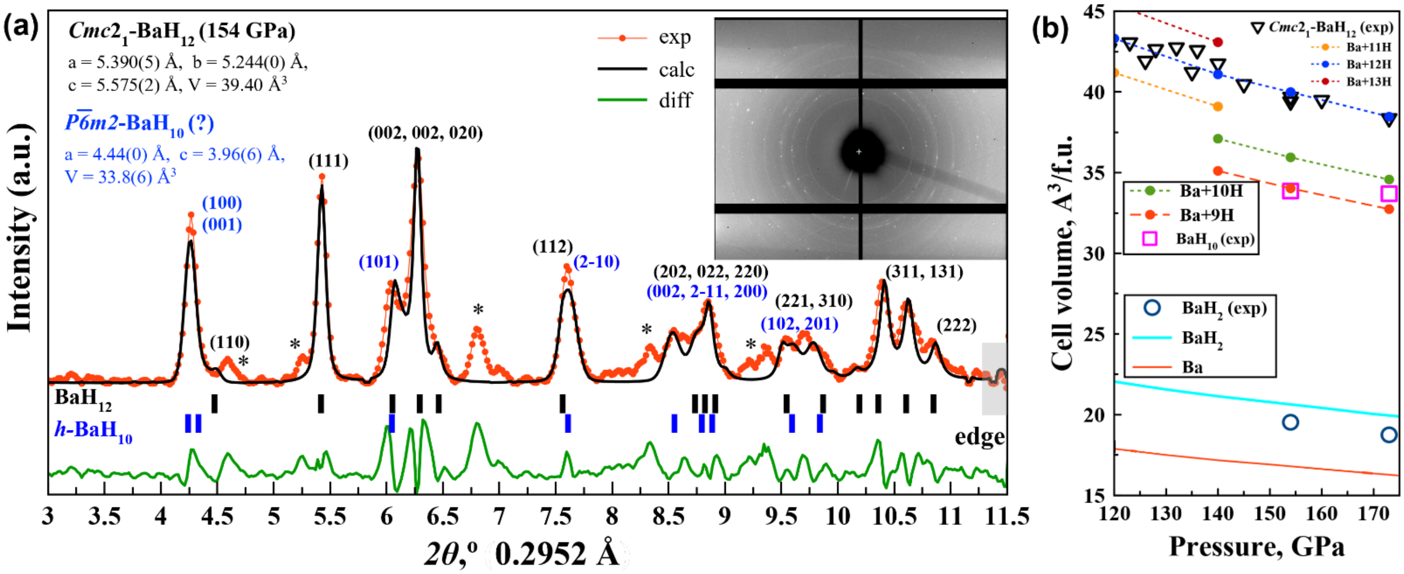

Fig. S29. (a) Experimental X-ray diffraction pattern and the Le Bail refinement of pseudocubic *Cmc*2₁-BaH_{12} and possible $P\bar{6}m2$-BaH_{10} from DAC #B0 at 154 GPa. The experimental data, fitted line, and residues are shown in red, black, and green, respectively. Edge: limitation of the diffraction angle. The cell was reoriented to get more reflections. Unidentified reflections are marked by asterisks. (b) Volume-pressure diagram showing the comparison of the experimental data with the theoretical predictions.

The volume and cell parameters of refined BaH_{12} (Table S13) are in close agreement with the DFT-predicted equation of state and the results of other experiments shown in Fig. S28b by black triangles.

Table S16. Experimental cell parameters of refined pseudocubic $Cmc2_1$-BaH_{12} and proposed $P\bar{6}m2$-BaH_{10} (DAC #B0). Volumes are given per Ba atom. When the pressure decreased below 154 GPa, DAC #B0 broke.

	Pressure, GPa	*a*, Å	*b*, Å	*c*, Å	*V*, Å^3	V_{DFT}, Å^3
BaH_{12}	173	5.299(4)	5.339(1)	5.394(6)	38.36	38.46
	154	5.390(5)	5.244(0)	5.575(2)	39.40	39.99
BaH_{10}	173	4.457(4)	-	3.915(7)	33.69	34.22
	154	4.44(0)	-	3.96(6)	33.8(6)	35.23

An additional experimental synthesis of barium hydrides was performed at 142 GPa in the DAC with a 100 µm culet, resulting in another compound whose X-ray diffraction pattern can be indexed mainly by the hexagonal space groups $P\bar{6}m2$ or *P6/mmm* (Fig. S31) with $a = 4.096$ Å and $c = 3.878$ Å. The analysis of the equations of state for the stoichiometric mixtures Ba+3H_2 and Ba+2.5H_2 shows that the new compound should have a composition close to $BaH_{\sim 6}$ (Fig. S32b). Alternatively, this pattern can correspond to metastable $P2/m$-BaH_{10} with $a = 3.738$ Å, $b = 3.88$ Å, $c = 5.234$ Å, $\beta = 107.01°$, and volume of 36.29 Å^3, which has almost the same XRD spectrum. The formation of lower barium hydrides instead of BaH_{12} at similar conditions may be caused by a lack of hydrogen, whose Raman signal was not detected after the laser heating, probably because of the full absorption by the Ba particle (see Raman Spectra section). We also cannot exclude formation of complex compounds as a result of the decomposition of ammonia borane, Ba–AB salts, and reactions of N and B with the sample, which may explain the extra reflections in XRD.

With a slight decrease in pressure to 139 GPa, this BaH_6 (or $P2/m$-BaH_{10}) phase decomposes, and the reflections previously identified as impurities (e.g., at 11.3°) become stronger (Fig. S32a). The XRD pattern becomes more complex with the appearance of many broad reflections from amorphous phases that are difficult to interpret. The cell parameters of the proposed hexagonal BaH_6 determined on the basis of the experimental data are presented in Table S17.

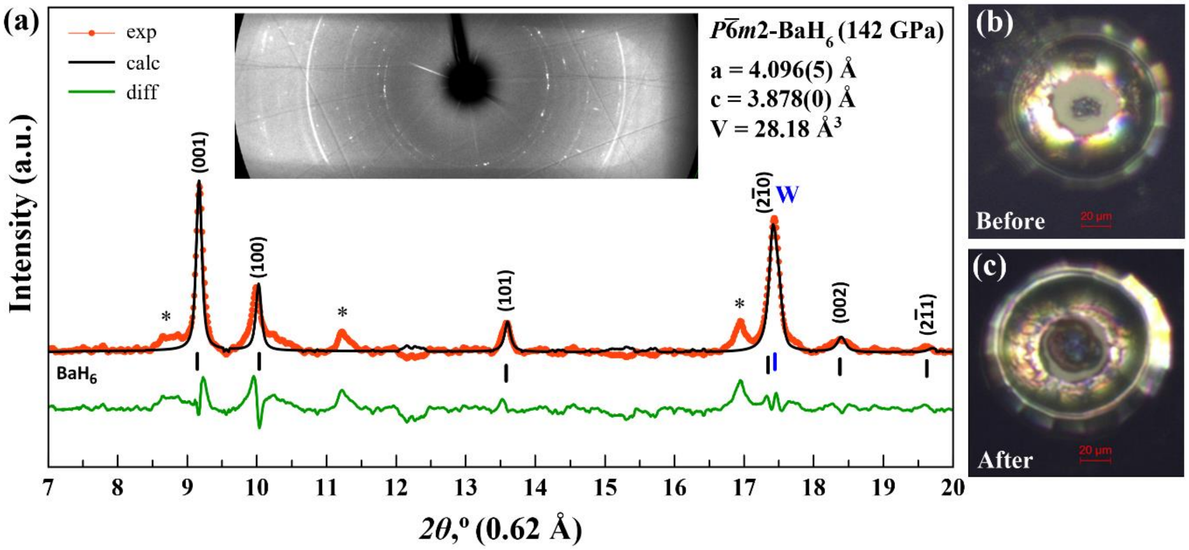

Fig. S30. (a) Experimental X-ray diffraction pattern and Le Bail refinement of $P\bar{6}m2$-BaH_6 at 142 GPa. (b, c) DAC before and after the laser heating. The sample underwent a substantial expansion; its optical transparency is heterogeneous.

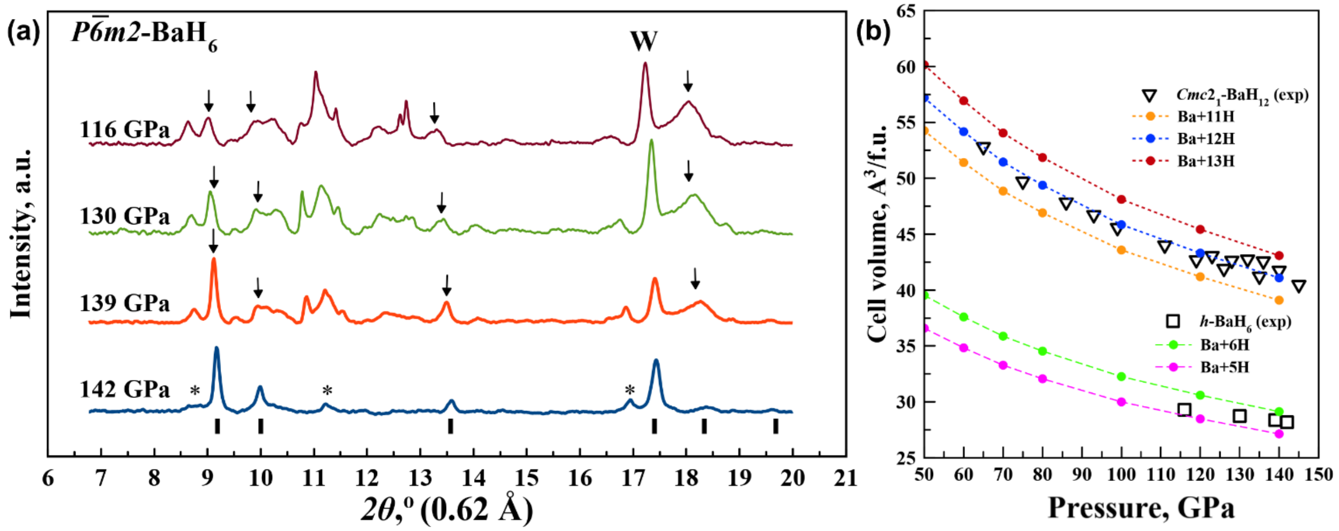

Fig. S31. (a) Experimental XRD patterns at 142–116 GPa demonstrating the decomposition of possible $P\bar{6}m2$-BaH_6 (marked by arrows). Uninterpreted reflections are marked by asterisks. (b) Equations of state of stoichiometric mixtures of Ba and H_2 with the experimental points of BaH_{12} and proposed BaH_6.

Table S17. Experimental cell parameters of refined proposed $P\bar{6}m2$-BaH_6. Volumes are given per Ba atom. The pressure was determined by the (110) reflex from the tungsten gasket.[36] When the pressure decreases below 116 GPa, the refinement using this structure is no longer possible.

Pressure, GPa	*a*, Å	*c*, Å	*V*, Å^3	V_{DFT} (*Imm*2),* Å^3
142	4.096(5)	3.878(0)	28.18	29.00
139	4.09(9)	3.89(9)	28.37	29.14
130	4.11(0)	3.92(9)	28.73	29.75
116	4.13(6)	3.95(4)	29.29	30.81

* The DFT results are given for *Imm*2-BaH_6, the most thermodynamically stable phase according to the USPEX calculations.

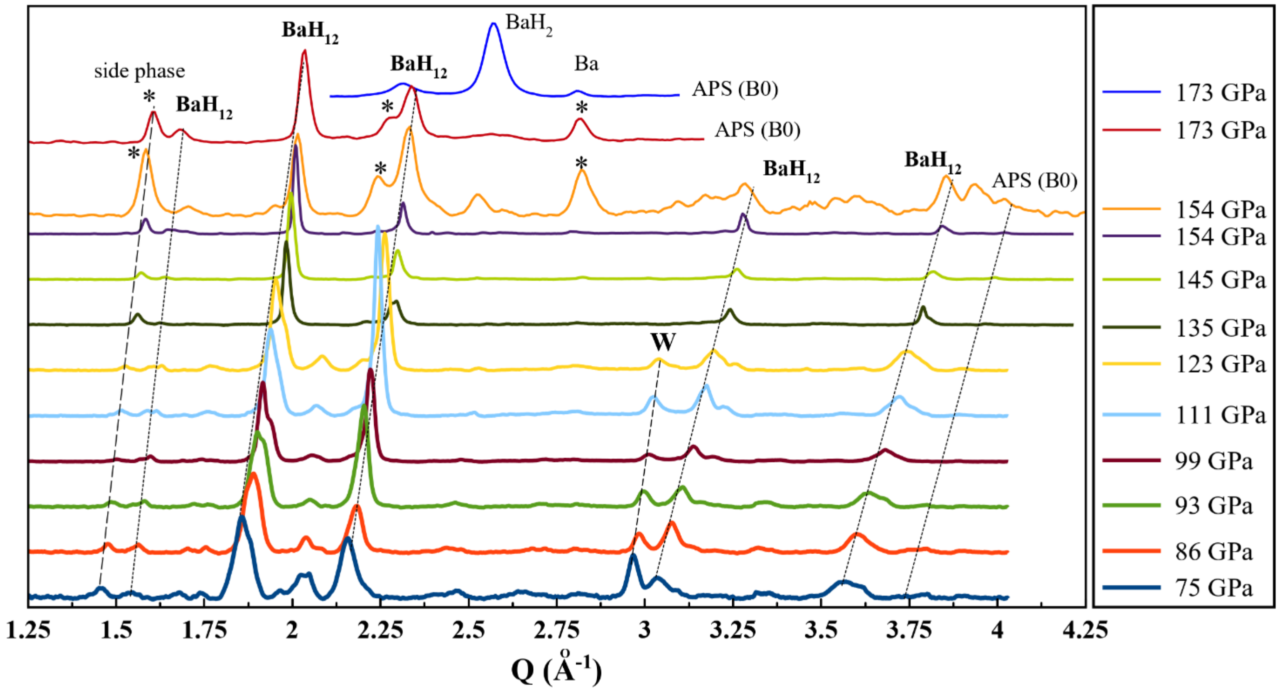

Fig. S32. Diffraction patterns from DACs #B0, B1, and B2 in q-space. A small amount of a side phase (*) synthesized in DAC #B0 at 154–173 GPa is also observed in other experiments.

Experimental Stability of BaH_{12}

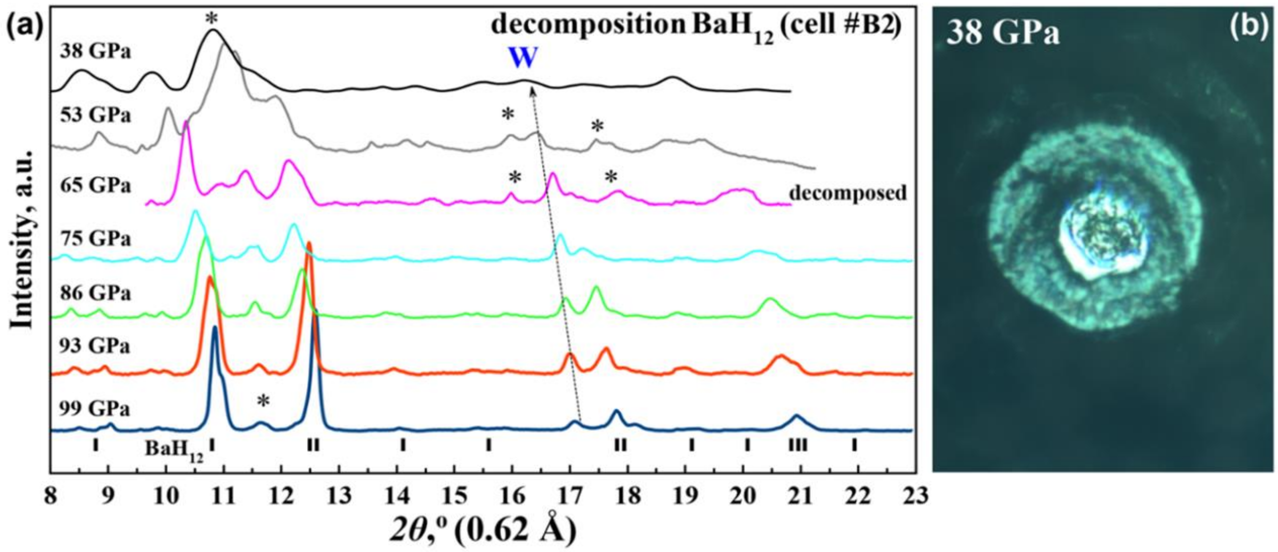

Fig. S33. (a) Experimental XRD patterns of BaH_{12} from cell #B2 at pressures of 99–38 GPa. The weak reflections from side phases and decomposition products are indicated by asterisks and a dotted line. (b) At 38 GPa, the sample becomes transparent and the observed XRD corresponds to the full decomposition of BaH_{12}. At 65 GPa, the (202) reflection of the *fcc* structure almost vanishes from the diffraction pattern, whereas the intensity of a side reflection at 11–11.5º, which is placed between (111) and (002), continues to grow.

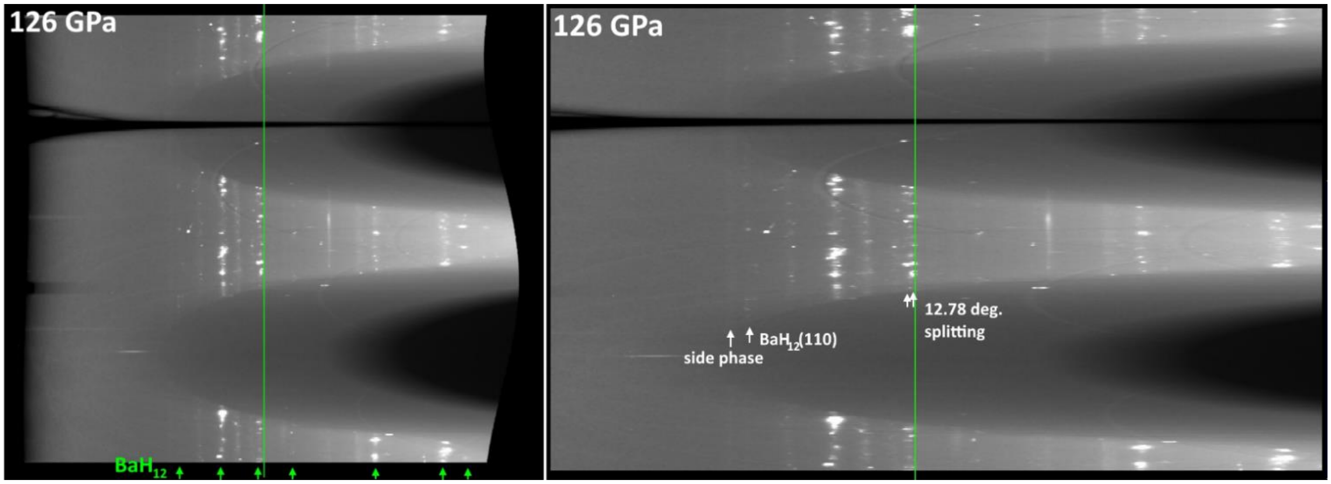

Fig. S34. XRD diffraction pattern (“cake”) of the BaH_{12} sample (DAC #B2) at 126 GPa. Green marks indicate the reflections with pronounced granularity belonging to pseudocubic BaH_{12}. At 12.8º, the splitting of the ideal *fcc* (002) reflection due to distortion (*Cmc*2_1) is shown. The side phase pattern does not have a pronounced texture.

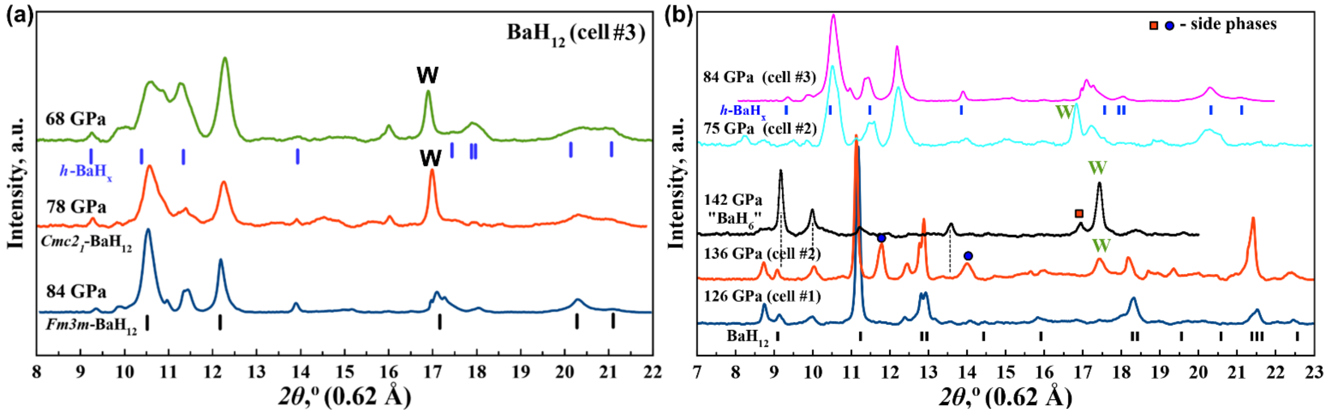

Fig. S35. (a) Decompression of DAC #B3 with the decomposition of pseudocubic BaH_{12} followed by an increase in the concentration of *h*-BaH_{12}. (b) Comparison of the impurities detected in DACs #B1–B3. The pressure points were selected to simplify the matching.

Electronic Properties of Barium Superhydrides

Spin-polarized calculations demonstrate that in the pressure range of 50–200 GPa all barium hydrides are nonmagnetic.

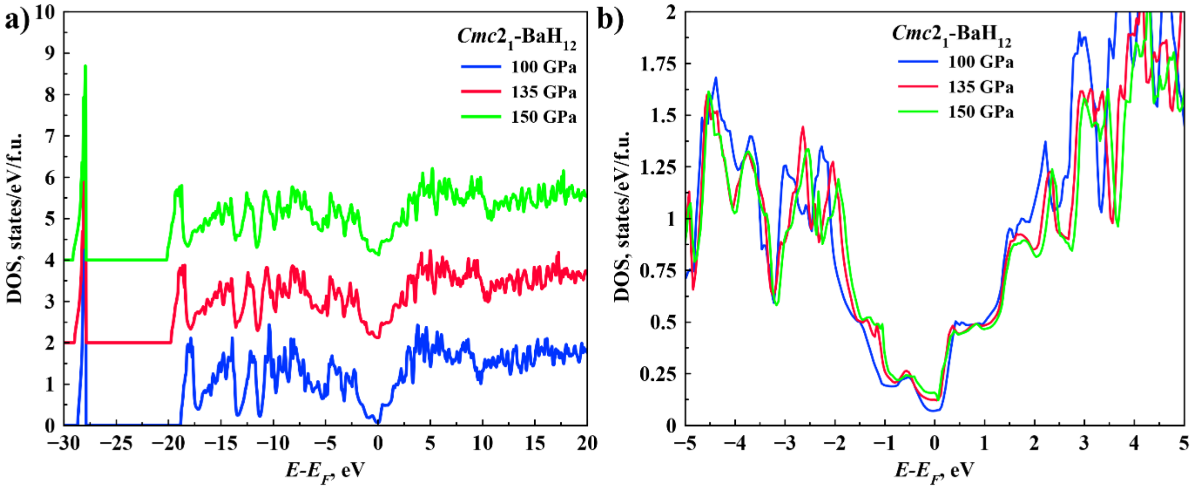

Fig. S36. Electron density of states of $Cmc2_1$-BaH$_{12}$ at 100, 135, and 150 GPa.

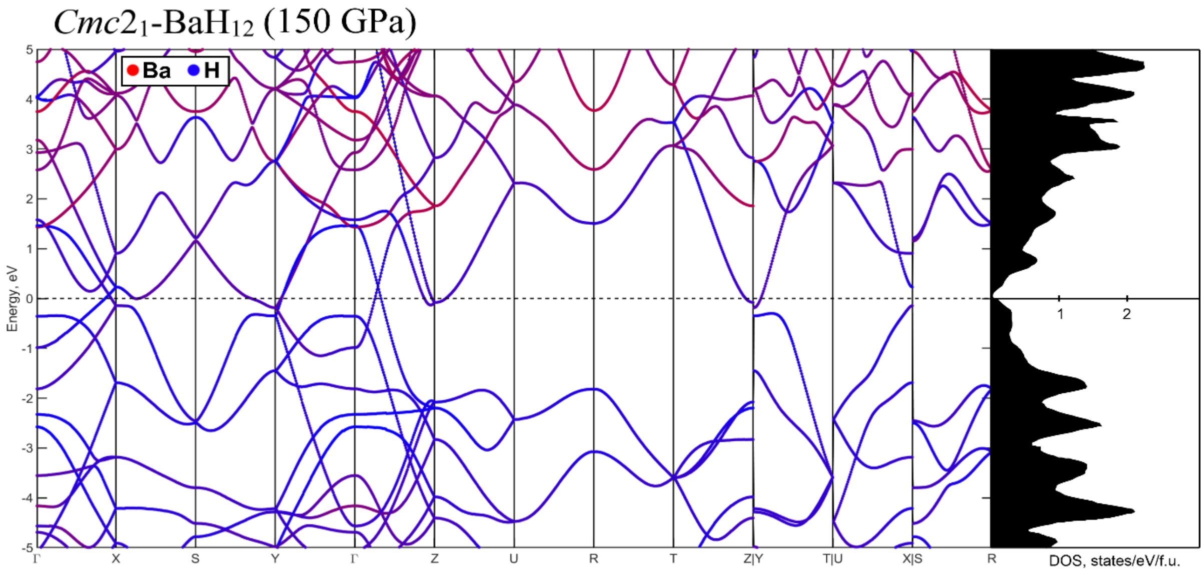

Fig. S37. Electron band structure of semimetallic $Cmc2_1$-BaH$_{12}$ at 150 GPa. Red and blue curves show the Ba and hydrogen bands, respectively. In the electronic band structure the Ba and H bands are well-separated, which suggests a weak interaction between the hydrogen and barium sublattices.

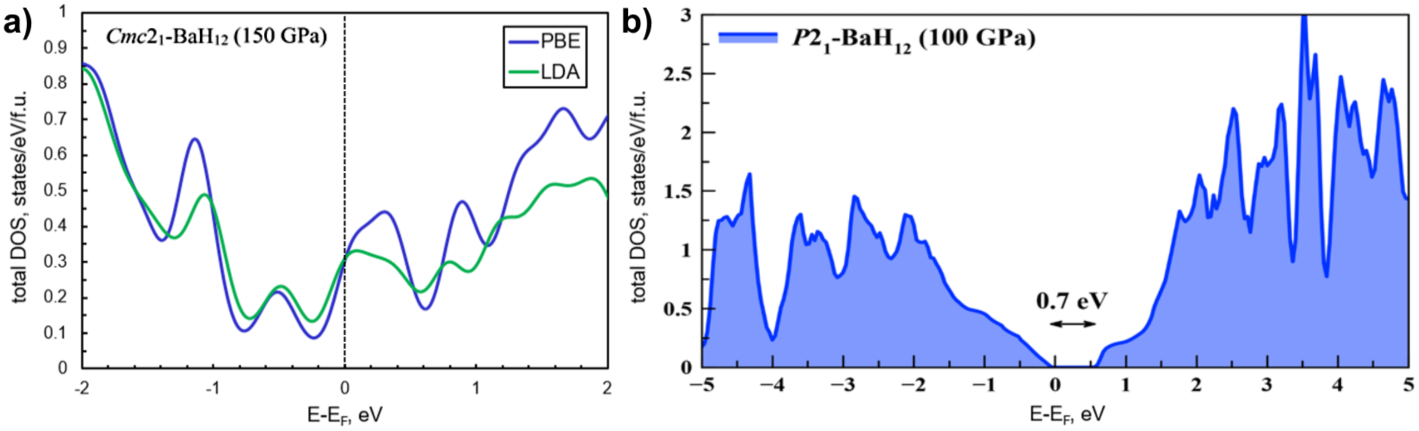

Fig. S38. (a) Electron density of states of $Cmc2_1$-BaH$_{12}$ at 150 GPa calculated using the PBE and LDA functionals with tetrahedron method. (b) Electron density of states of stable $P2_1$-BaH$_{12}$ at 100 GPa.

Bader Charges

The charge of the barium atoms in BaH_{12} is +1.15 at 150 GPa, whereas most of the hydrogen atoms have a negative charge. In the H_3 fragments, the charge of the end atoms is close to –0.2 and –0.27, while the H bridge has a small positive charge of +0.06. In general, H_3^- anion has a total charge of –0.4|*e*|, whereas H_2 molecular fragments ($d_{H–H}$ = 0.78 Å) have a charge of only –0.1|*e*|.

Table S18. Bader atomic charges of *Cmc*2_1-BaH_{12} at 150 GPa calculated using Critic2 software.

Atom	Z	Electron population	Bader charge
Ba1	10	8.85	1.153
Ba2	10	8.85	1.153
H1	1	1.27	-0.266
H2	1	1.27	-0.266
H3	1	1.27	-0.266
H4	1	1.27	-0.266
H5	1	1.07	-0.067
H6	1	1.07	-0.067
H7	1	1.07	-0.067
H8	1	1.07	-0.067
H9	1	0.941	0.059
H10	1	0.941	0.059
H11	1	0.941	0.059
H12	1	0.941	0.059
H13	1	1.12	-0.122
H14	1	1.12	-0.122
H15	1	1.12	-0.122
H16	1	1.12	-0.122
H17	1	1.20	-0.197
H18	1	1.20	-0.197
H19	1	1.20	-0.197
H20	1	1.20	-0.197
H21	1	0.983	0.017
H22	1	0.983	0.017
H23	1	0.983	0.017
H24	1	0.983	0.017
Total	44	44.0	0.000

Raman Spectra

Raman spectra were recorded on LabRAM HR Evol spectrometer, green and red lasers with wavelengths of 532 and 633 nm were used, the signal accumulation time was 20 s, diffraction grating was 1800 grates/mm, frequency range studied was from 100 to 4500 cm^{-1}.

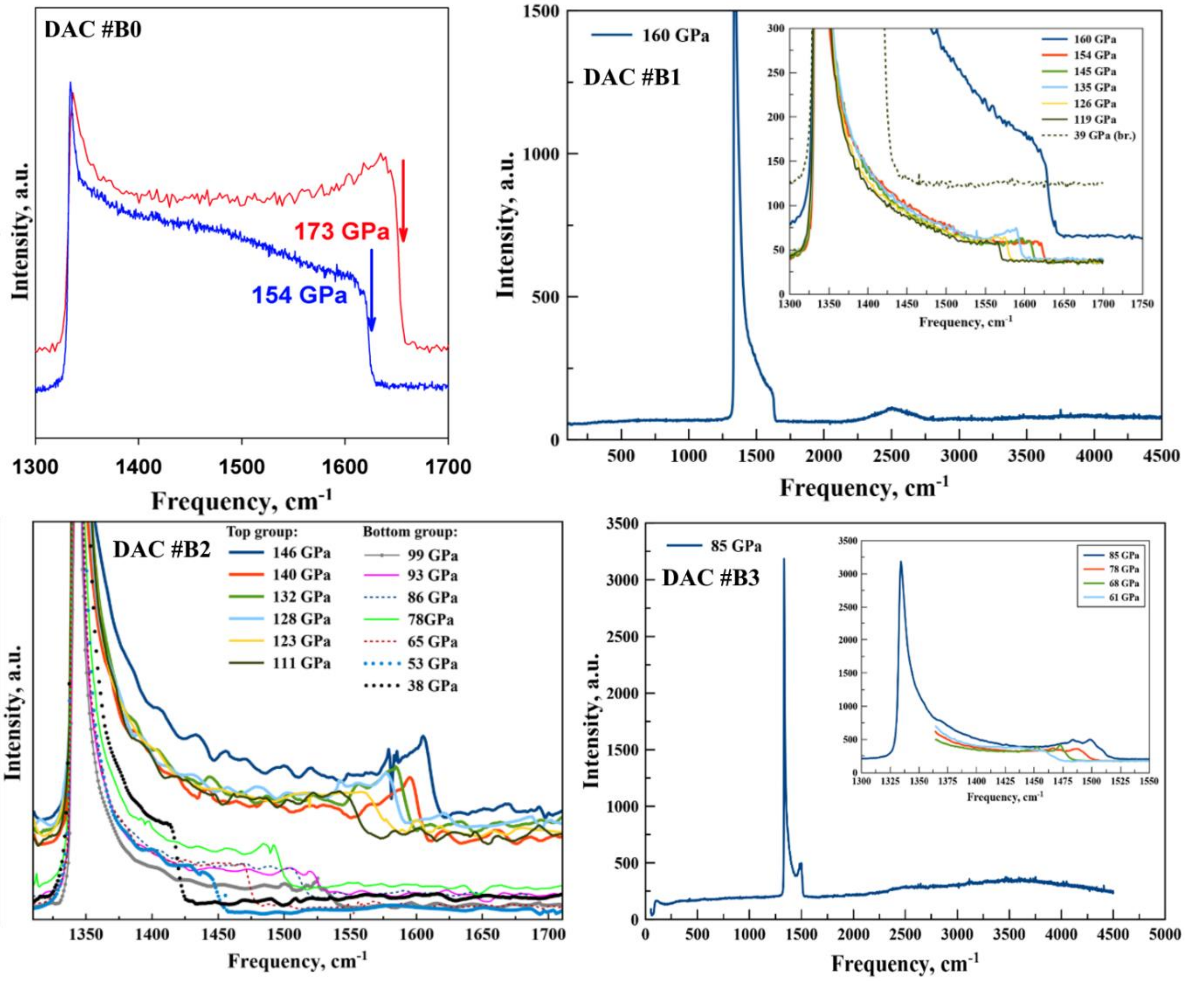

Fig. S39. Raman spectra of DACs #B0–B3. Hydrogen was not detected in the center of the samples, possibly because of the complete absorption of the samples (H_2 was detected at their edges). No signals from nonmetallic phases were present.

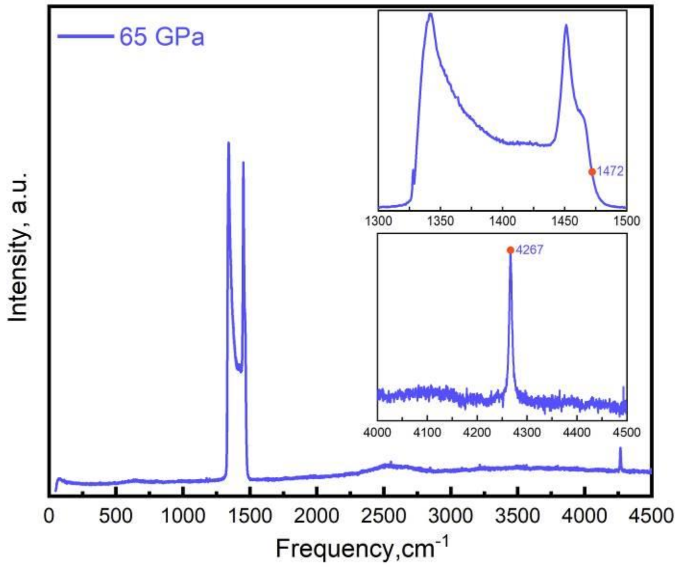

Fig. S40. Raman spectra of the crashed DAC #B4 (140 → 65 GPa) with the measured superconducting transition at 20 K (at 140 GPa).

Elastic properties

The elastic tensors of the *Cmc*2$_1$-BaH$_{12}$ were calculated using the stress-strain relations:

$$C_{ij} = \frac{\partial \sigma_i}{\partial \eta_j}, \quad \text{(S2)}$$

where σ_i is the i-th component of the stress tensor, η_j is the j-th component of the strain tensor.

The bulk (B) and shear (G) moduli and Young's modulus (E) were calculated in GPa via Voigt-Reuss-Hill averaging. [37,38] Using obtained values of elastic moduli, we calculated the velocities of longitudinal and transverse acoustic waves:

$$v_{LA} = \sqrt{\frac{C_{11}}{\rho}}, \quad v_{TA} = \sqrt{\frac{C_{11} - C_{12}}{2\rho}}, \quad \text{(S3)}$$

where C_{11}, C_{12} are elastic constants, ρ is a density of compound. Obtained values allow us to estimate Debye temperature as [39]:

$$\vartheta_D = \frac{h}{k_B}\left[\frac{3n}{4\pi}\left(\frac{N_A \cdot \rho}{M}\right)\right]^{\frac{1}{3}} v_m, \quad \text{(S4)}$$

where h, k_B, N_A are Planck's, Boltzmann's and Avogadro constants, v_m is an average velocity of acoustic waves calculated by the following formula

$$v_m = \left[\frac{1}{3}\left(\frac{2}{v_{TA}^3} + \frac{1}{v_{LA}^3}\right)\right]^{-1/3}. \quad \text{(S5)}$$

Table S19. Elastic and thermodynamic parameters of *P*2$_1$-BaH$_{12}$ (Z = 4) at 140 GPa.

Parameter	140 GPa
a, Å	5.4394
b, Å	5.4461
c, Å	5.4286
V_{DFT}, Å^3	40.2 (per f.u.)
C_{11}, GPa	831
C_{12}, GPa	314
C_{13}, GPa	255
C_{22}, GPa	802
C_{23}, GPa	213
C_{33}, GPa	902
C_{44}, GPa	82
C_{55}, GPa	165
C_{66}, GPa	175
B, GPa	443
G, GPa	181
E, GPa	479
Poisson ratio η	0.32
Density ρ, kg/m^3	6167
Transverse sound velocity v_t, m/s	5421
Longitudinal sound velocity v_l, m/s	10536
Debye temperature θ_D, K	1241

Temperature Dependence of Resistance

As in all metal hydrides and polyhydrides, metal atoms donate some electrons to the hydrogen atoms. These electrons occupy the antibonding orbitals in the H_2 molecules and weaken the H–H bonds. If no electrons are transferred, the H_2 molecules will persist and will not contribute to superconductivity. If one electron is transferred, the hydride H^- ions will be formed, with no H–H bonds and little or no contribution to superconductivity. At intermediate electron doping levels (it has been found[40,41] that the optimum is ~0.3 electrons), weak H–H bonds (e.g., as in clathrate polyhydrides) are formed. In BaH_{12}, each H atom accepts on average 0.16 electrons. As a result, H_2 and H_3 groups are still present in the structure, and T_C is rather low. Using the standard BCS formalism (see below), we also estimated for *Cmc*2_1-BaH_{12} the coherence length ξ_{BCS} = 68 Å (μ^* = 0.1), superconducting gap of ~9 meV, and isotope coefficient α = 0.47 at 150 GPa.

Table S20. Experimental parameters of electric DACs that were used to measure the temperature dependence of the resistance of barium hydrides. The current was 1 mA.

Cell #	Synthesis pressure, GPa	Culet size, µm	Sample size, µm	Composition
E1	120	100	55×41	Ba/BH_3NH_3
E2	104	100	77×40	Ba/BH_3NH_3
E3	100	100	66×59	Ba/BH_3NH_3
E4	132	100	12×28	Ba/BH_3NH_3
E5*	140	80	45×32	Ba/BH_3NH_3

*After the laser heating at 1600 K. When we tried to change the pressure, the cell collapsed and the pressure dropped to 65 GPa.

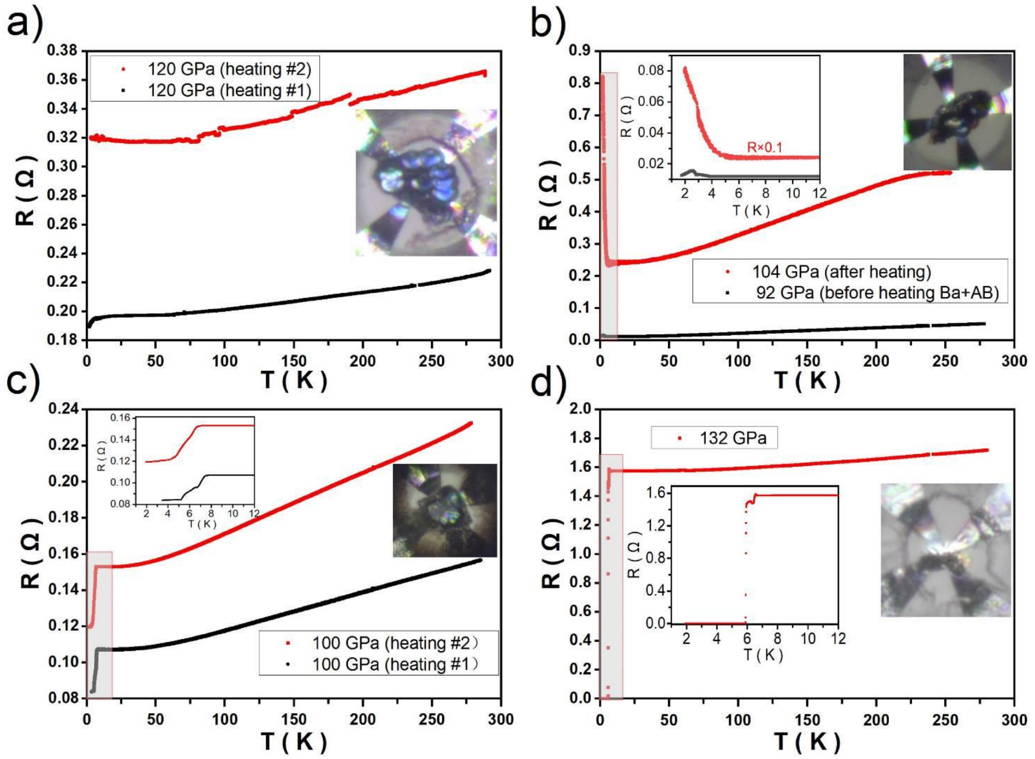

Fig. S41. Temperature dependence of the resistance *R*(*T*) of BaH_x samples prepared by the laser heating of Ba/AB at (a) 120 GPa, (b) 92 and 104 GPa, (c) 100 GPa, and (d) 132 GPa. All measurements were performed using the four-probe technique. The resistance of all samples increased after each heating. The detected sharp resistance drops (or jump in (b)) around 5–7 K may relate to the superconducting transitions in the BaH_x samples. In all the studied cells, the BaH_x samples demonstrated clear metallic behavior of *R*(*T*).

It is possible to estimate the Debye temperature (θ_D) of the compressed BaH_{12} from the dependence of electrical resistance on temperature (Fig. S41) using the fit of experimental R(T) to Bloch-Grüneisen formula [42,43]:

$$R(T) = R_0 + A\left(\frac{T}{\theta_D}\right)^5 \int_0^{\theta D/T} \frac{x^5}{(e^x - 1)(1 - e^{-x})} dx \qquad \text{(S6)}$$

where the first two coefficients R_0, which is related to defects of crystalline lattice, and A – may be defined by maximum and minimum of R(T), while θ_D – is a free-fitting parameter.

At 140 GPa the measured R-T data has sufficient quality to determine $\theta_D(BaH_{12})$ that was found to be 520-560 K. At 132 GPa similar θ_D = 550 K was found. Despite the fact that the McMillan's $T_C(BaH_{12})$, calculated using the obtained θ_D is in better agreement with the experimental data than calculated from $\alpha^2F(\omega)$, the estimates should be carefully considered, as we do not take into account the contribution to R(T) from Mo-electrodes used in these E1-E5 cells.

Superconductivity

To calculate the isotopic coefficient β, the Allen–Dynes interpolation formulas[22] were used:

$$\beta_{McM} = -\frac{d\ln T_C}{d\ln M} = \frac{1}{2}\left[1 - \frac{1.04(1+\lambda)(1+0.62\lambda)}{[\lambda - \mu^*(1+0.62\lambda)]^2}\mu^{*2}\right], \quad \text{(S7)}$$

$$\beta_{AD} = \beta_{McM} - \frac{2.34\mu^{*2}\lambda^{3/2}}{(2.46+9.25\mu^*)\cdot((2.46+9.25\mu^*)^{3/2}+\lambda^{3/2})} - \frac{130.4\cdot\mu^{*2}\lambda^2(1+6.3\mu^*)\left(1-\frac{\omega_{\log}}{\omega_2}\right)\frac{\omega_{\log}}{\omega_2}}{\left(8.28+104\mu^*+329\mu^{*2}+2.5\cdot\lambda^2\frac{\omega_{\log}}{\omega_2}\right)\cdot\left(8.28+104\mu^*+329\mu^{*2}+2.5\cdot\lambda^2\left(\frac{\omega_{\log}}{\omega_2}\right)^2\right)}, \quad \text{(S8)}$$

where the last two correction terms are usually small (~0.01).

The superconducting transition temperature T_C was estimated using the Allen–Dynes formula[22] in the following form:

$$T_C = \omega_{log}\frac{f_1 f_2}{1.2}\exp\left(\frac{-1.04(1+\lambda)}{\lambda-\mu^*-0.62\lambda\mu^*}\right), \quad \text{(S9)}$$

where

$$f_1 f_2 = \sqrt[3]{1+\left(\frac{\lambda}{2.46(1+3.8\mu^*)}\right)^{\frac{3}{2}}}\cdot\left(1-\frac{\lambda^2(1-\omega_2/\omega_{log})}{\lambda^2+3.312(1+6.3\mu^*)^2}\right). \quad \text{(S10)}$$

The EPC constant λ, logarithmic average frequency ω_{log}, and mean square frequency ω_2 were calculated as:

$$\lambda = \int_0^{\omega_{max}} \frac{2\cdot\alpha^2 F(\omega)}{\omega}d\omega, \quad \text{(S11)}$$

$$\omega_{log} = \exp\left(\frac{2}{\lambda}\int_0^{\omega_{max}}\frac{d\omega}{\omega}\alpha^2 F(\omega)\ln(\omega)\right), \qquad \omega_2 = \sqrt{\frac{1}{\lambda}\int_0^{\omega_{max}}\left[\frac{2\alpha^2 F(\omega)}{\omega}\right]\omega^2 d\omega}, \quad \text{(S12)}$$

where μ^* is the Coulomb pseudopotential, for which we used widely accepted lower and upper bounds of 0.10 and 0.15.

The Sommerfeld constant was found as

$$\gamma = \frac{2}{3}\pi^2 k_B{}^2 N(0)(1+\lambda), \quad \text{(S13)}$$

and was used to estimate the upper critical magnetic field and superconducting gap in BaH_{12} at 150 GPa using the well-known semiempirical equations of the BCS theory (see Ref.[44], equations 4.1 and 5.11), working satisfactorily for $T_C/\omega_{log} < 0.25$:

$$\frac{\gamma T_C{}^2}{(\mu_0 H_{C_2}(0))^2} = 0.168\left[1-12.2\left(\frac{T_C}{\omega_{\log}}\right)^2\ln\left(\frac{\omega_{\log}}{3T_C}\right)\right], \quad \text{(S14)}$$

$$\frac{2\Delta(0)}{k_B T_C} = 3.53\left[1+12.5\left(\frac{T_C}{\omega_{\log}}\right)^2\ln\left(\frac{\omega_{\log}}{2T_C}\right)\right]. \quad \text{(S15)}$$

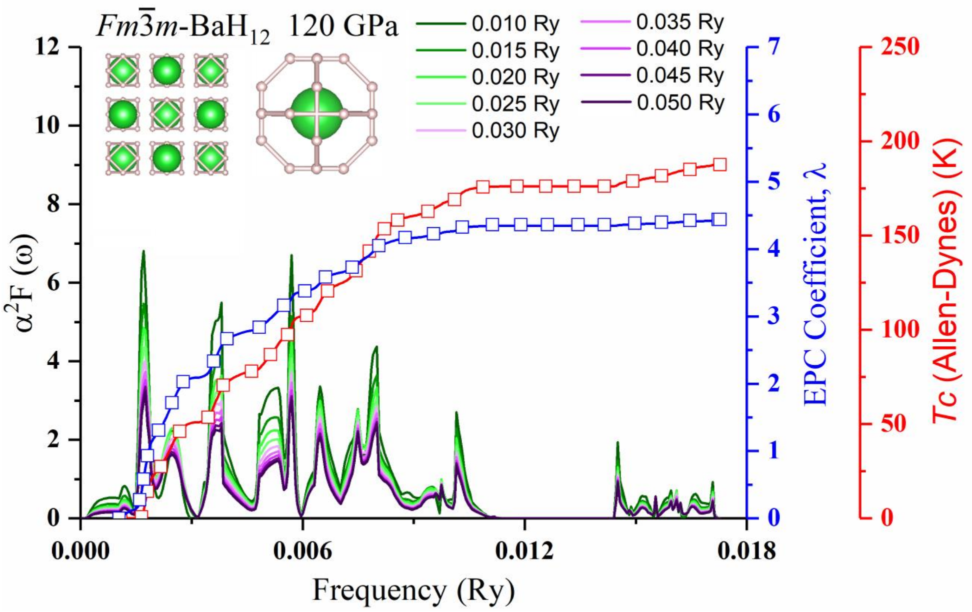

Fig. S42. Eliashberg function and superconducting parameters of *Fm*$\overline{3}$*m*-BaH_{12} with the structure of *Fm*$\overline{3}$*m*-YB_{12} at 120 GPa. The cell parameters are given in Tables S1 and S2.

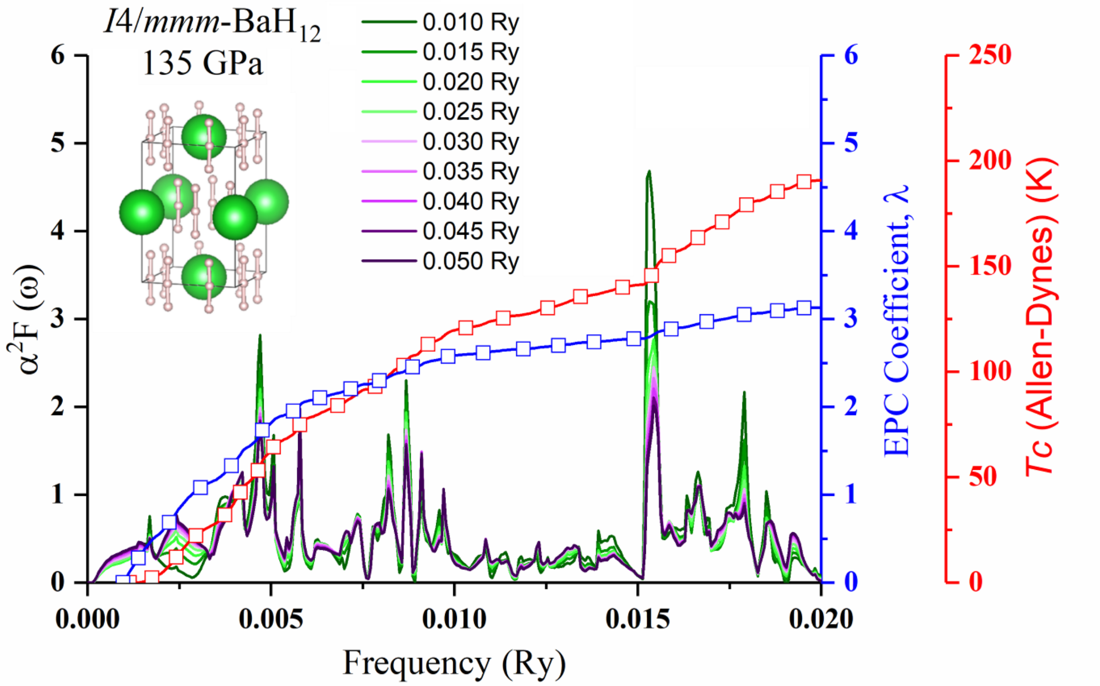

Fig. S43. Eliashberg function and superconducting parameters of *I4/mmm*-BaH_{12} at 135 GPa. The structure parameters are given in Table S1.

Scripts for XRD Postprocessing with USPEX

1. split_CIFs.py

The script splits the structures with the lowest enthalpy of a variable-composition USPEX run into multiple CIF files. It takes the following arguments from the command line:

1. the output file extended_convex_hull from USPEX
2. the output file extended_convex_hull_POSCARS from USPEX
3. the value of the external pressure used for the USPEX run

The script performs the following operations:

1. for each structure, reads the parameters enthalpy and fitness from extended_convex_hull and the geometry from extended_convex_hull_POSCARS
2. for each reduced formula, selects five structures with the lowest enthalpy
3. outputs the selected structures as CIF files into a new *results* folder

The name of each CIF file has the format: i_ID_fitness_enthalpy_iupacformula_pressure_symmetry.cif, where

i is a natural number that orders the output by increasing fitness
ID is the structure ID from the USPEX run
fitness is the fitness of the structure
enthalpy is the enthalpy of the structure
iupacformula is the IUPAC formula of the structure
pressure is the pressure used for the USPEX run
symmetry is the space group number, determined with a tolerance of 0.2

<u>Example</u>: python split_CIFs.py extended_convex_hull extended_convex_hull_POSCARS 50GPa

2. sublattice_split_CIFs.py

The script reads the results of a variable composition USPEX run, removes all the hydrogen atoms, and splits the structures with the lowest enthalpy into multiple CIF files. It takes the following arguments from the command line:

1. the output file extended_convex_hull from USPEX
2. the output file extended_convex_hull_POSCARS from USPEX
3. the value of the external pressure used for the USPEX run

The script performs the following operations:

1. for each structure, reads the parameters enthalpy and fitness from extended_convex_hull and the geometry from extended_convex_hull_POSCARS
2. removes all the hydrogen atoms
3. for each reduced formula, selects five structures with the lowest enthalpy
4. outputs the selected structures as CIF files into a new *results* folder

The name of each CIF file has the format i_ID_fitness_enthalpy_iupacformula_pressure_symmetry.cif, where

i is a natural number that orders the output by increasing fitness
ID is the structure ID from the USPEX run
fitness is the fitness of the structure
enthalpy is the enthalpy of the structure
iupacformula is the IUPAC formula of the structure, with hydrogen
pressure is the pressure used for the USPEX run
symmetry is the space group number, determined with a tolerance of 0.2 and without hydrogen

Example: python sublattice_split_CIFs.py extended_convex_hull extended_convex_hull_POSCARS 50GPa

3. fixcomp_split_CIFs.py

The script splits all the structures of a fixed composition USPEX run into multiple CIF files. It takes the following arguments from the command line:

1. the output file Individuals from USPEX
2. the output file gatheredPOSCARS from USPEX
3. the value of the external pressure used for the USPEX run

The script performs the following operations:

1. for each structure, reads the parameter enthalpy from Individuals and the geometry from gatheredPOSCARS
2. computes the real fitness = enthalpy / total number of atoms
3. outputs the structures as CIF files in a new *results* folder

The name of each CIF file has the format i_ID_realfitness_enthalpy_iupacformula_pressure_symmetry.cif, where

i is a natural number that orders the output by increasing real fitness
ID is the structure ID from the USPEX run
realfitness is the real fitness of the structure
enthalpy is the enthalpy of the structure
iupacformula is the IUPAC formula of the structure
pressure is the pressure used for the USPEX run
symmetry is the space group number, determined with a tolerance of 0.2

Example: python fixcomp_split_CIFs.py Individuals gatheredPOSCARS 50GPa

4. exclusion.py

The script quickly filters a multitude of CIF files, removing those which have significant peaks in a user-defined exclusion region of the X-ray spectrum. It takes the following arguments from the command line:

1. the wavelength of the incident radiation in angstroms
2. the peak cutoff, in percent of the maximum intensity
3. the number of intervals in degrees, expressed as two angles separated by a hyphen (-), defining the exclusion region

The script works in a folder with many CIF files and performs the following:

1. opens, one by one, all CIF files and predicts the XRD pattern of the structure according to the given wavelength
2. if the predicted pattern contains any peak in the exclusion regions that is bigger than the given cutoff, deletes the CIF file

Example: python exclusion.py 0.6199 25 25-28 31-32

5. find_peak.py

The script quickly filters a multitude of CIF files, removing those which do not have significant peaks in all user-defined search intervals of the X-ray spectrum. It takes the following arguments from the command line:

1. the wavelength of the incident radiation in angstroms
2. the peak cutoff, in percent of the maximum intensity
3. the number of search intervals in degrees, expressed as two angles separated by a hyphen (-)

The script works in a folder with many CIF files and performs the following:

1. it opens, one by one, all CIF files and predicts the XRD pattern of the structure according to the given wavelength
2. if there is at least one search region which does not contain any peak bigger than the given cutoff, deletes the CIF file

Example: python find peak.py 0.6199 15 25-28 31-32

6. change_pressure.py

The script translates the structures contained in many CIF files to a different pressure, deforming the lattice parameters using a second-order Taylor expansion of the Birch–Murnaghan equation. More details about the underlying theory can be found in the description of the script xr_screening.py.

The script takes the following arguments from the command line:

1. the initial pressure
2. the final pressure

The script works in a folder with many CIF files and performs the following:

1. reads the structures contained in all CIF files
2. deforms the lattice parameters according to the new pressure
3. creates new CIF files containing the deformed structures

The name of the new CIF files has the format OLDNAME_toNEWPRESSURE, where

OLDNAME is the name of the file with the structure at the initial pressure
NEWPRESSURE is the final pressure

Example: python change pressure.py 50 58

7. relax_new_pressure.py

The script makes an accurate translation of the structures contained in many CIF files to a different pressure, relaxing them using VASP.[15-17] It uses the VASP calculator class of the Atomic Simulation Environment (ASE) library[45] and is meant to be run on a computer cluster.

The script takes the following arguments from the command line:

1. the initial pressure
2. the final pressure

In addition, the parameters of the VASP calculations need to be tuned by hand, by editing the file relax_new_pressure.py. The script works in a folder with many CIF files and performs the following:

1. reads the structures contained in all CIF files
2. launches VASP for relaxing the structures at the desired final pressure
3. creates new CIF files containing the reoptimized structures

The name of the new CIF files has the format OLDNAME_toNEWPRESSURE, where

OLDNAME is the name of the file with the structure at the initial pressure
NEWPRESSURE is the final pressure

By default, VASP relaxations are performed sequentially, and every relaxation takes four computing cores. For more details, refer to the documentation of the ASE VASP calculator.

8. xr screening.py

The script performs a screening of the USPEX results, looking for the structures that best match the experimental XRD spectrum. It contains a number of various input parameters.

The script performs the following operations:

1. for each structure, reads the geometry from gatheredPOSCARS
2. computes the theoretical powder XRD spectrum using pymatgen[7]
3. computes the fitness, describing how much the theoretical spectrum agrees with the experimental one
4. outputs a graph with the theoretical and experimental spectra, where the file name starts with the value of the computed fitness

We developed a code which computes, from the experimental spectrum and the USPEX output, the degree of agreement (fitness) of each relaxed structure with the experimental data. The USPEX calculations and the experimental spectrum do not need to be exactly at the same pressure, but the two pressures need to be no more than 20 GPa apart. Because we have a pressure difference, we first translate each calculated structure into the experimental pressure using the Birch–Murnaghan equation:

$$\Delta P = \frac{3B_0}{2}\left[\left(\frac{V_0}{V}\right)^{\frac{7}{3}} - \left(\frac{V_0}{V}\right)^{\frac{5}{3}}\right]\left\{1 + \frac{3}{4}\left(B_0' - 4\right)\left[\left(\frac{V_0}{V}\right)^{\frac{2}{3}} - 1\right]\right\} \quad \text{(S16)}$$

where ΔP is the pressure difference, V_0 is the volume of the unit cell at the calculated pressure, V is the volume at the experimental pressure, B_0 is the bulk modulus, and B_0' is its derivative with respect to pressure. In this work we used $B_0 = 300$ GPa and $B_0' = 4$.

Assuming that for small pressure variations V_0/V will also be close to 1, we approximate the Birch–Murnaghan equation by a second-order Taylor expansion and get the volume V at the experimental pressure as:

$$V = \frac{300}{150 + \sqrt{22500 + 300\Delta P}} V_0 \quad \text{(S17)}$$

Then we define the following scaling factor:

$$k = \sqrt[3]{\frac{300}{150 + \sqrt{22500 + 300\Delta P}}} \quad \text{(S18)}$$

that will be used to rescale the lattice parameters of all the calculated structures.

After this rescaling, the relaxed structures are symmetrized with a tolerance of 0.2 and the theoretical XRD spectra are computed. Both theoretical and experimental spectra are in the form of a series of peaks. For each peak we know the diffraction angle and relative intensity (the intensity of the highest peak in each spectrum has been conventionally given the value 100). We define a *match* between a theoretical and an experimental peak if the two peaks are less than 0.25º apart, regardless of their intensities.

The fitness between the calculated and experimental XRD spectrum is defined by the following Gaussian-like fitness function:

$$\begin{aligned} F = &\sum_{i,j}^{match} \frac{(x_i^{exp} - x_j^{th})^2}{\Delta\alpha^2} f(h_i^{exp}) + \sum_{i}^{rest} \frac{(x_i^{exp})^2}{\Delta\alpha^2} f(h_i^{exp}) + \sum_{i}^{rest} \frac{(x_i^{th})^2}{\Delta\alpha^2} f(h_i^{th}) \\ &\sum_{i,j}^{match} \frac{(h_i^{exp} - h_j^{th})^2}{100^2} f(h_i^{exp}) + \sum_{i}^{rest} \frac{(h_i^{exp})^2}{100^2} f(h_i^{exp}) + \sum_{i}^{rest} \frac{(h_i^{th})^2}{100^2} f(h_i^{th}) \end{aligned} \quad \text{(S19)}$$

where x_i are diffraction angles, h_i are intensities, $\Delta\alpha$ is the total width of the spectra, and $f(h_i)$ is the weight function defined as:

$$f(t) = \begin{cases} 5 & \text{if } t > 90 \\ 1 & \text{if } 50 < t \leq 90 \\ 0.25 & \text{if } 10 < t \leq 50 \\ 0.02 & \text{if } 1 < t \leq 10 \\ 0 & \text{if } t \leq 1 \end{cases} \quad \text{(S20)}$$

The first term of the fitness is a sum over the matched peaks, and the closer the two peaks are to each other, the smaller each term of this sum is. At the denominator is the total width $\Delta\alpha$ of the spectra, which is the maximum value for an angle, allowing us to get dimensionless terms. The weight coefficients determine that higher peaks make a

more significant contribution to the fitness than smaller peaks, and that peaks with an intensity smaller than 1 make no contribution. The second term is a sum over the experimental peaks that are left after our matching, we call these peaks *experimental rest*. It has a similar form as the first term, but its addends have a much higher value because there is no subtraction in the numerator. The third term, analogously, is a sum over the *theoretical rest*. The remaining three terms are very similar to the first three, with intensities in place of angles.

A low value of F means close agreement between the calculated and experimental spectra, making possible a quick identification of promising candidates in the USPEX output.

References

1. Akahama, Y. & Kawamura, H. Pressure calibration of diamond anvil Raman gauge to 410 GPa. *J. Phys.: Conf. Ser.* **215**, 012195 (2010).
2. Prescher, C. & Prakapenka, V. B. DIOPTAS: a program for reduction of two-dimensional X-ray diffraction data and data exploration. *High Pressure Research* **35**, 223–230 (2015).
3. Paufler, P. R. A. Young (ed.). The Rietveld Method. International Union of Crystallography. Oxford University Press 1993. 298 p. Price £ 45.00. ISBN 0–19–855577–6. *Crystal Research and Technology* **30**, 494–494 (1995).
4. Petříček, V., Dušek, M. & Palatinus, L. Crystallographic Computing System JANA2006: General features. *Zeitschrift für Kristallographie - Crystalline Materials* **229**, 345–352 (2014).
5. Bail, A. L. Whole powder pattern decomposition methods and applications: A retrospection. *Powder Diffraction* **20**, 316–326 (2005).
6. Birch, F. Finite Elastic Strain of Cubic Crystals. *Phys. Rev.* **71**, 809–824 (1947).
7. Ong, S. P. *et al.* Python Materials Genomics (pymatgen): A robust, open-source python library for materials analysis. *Comp. Mat. Sci.* **68**, 314–319 (2013).
8. Hohenberg, P. & Kohn, W. Inhomogeneous electron gas. *Phys Rev* **136**, B864–B871 (1964).
9. Kohn, W. & Sham, L. J. Self-consistent equations including exchange and correlation effects. *Phys Rev* **140**, A1133–A1138 (1965).
10. Perdew, J. P., Burke, K. & Ernzerhof, M. Generalized gradient approximation made simple. *Physical review letters* **77**, 3865–3868 (1996).
11. Blöchl, P. E. Projector augmented-wave method. *Phys. Rev. B* **50**, 17953–17979 (1994).
12. Kresse, G. & Joubert, D. From ultrasoft pseudopotentials to the projector augmented-wave method. *Phys. Rev. B* **59**, 1758–1775 (1999).
13. Kresse, G. & Furthmüller, J. Efficient iterative schemes for ab initio total-energy calculations using a plane-wave basis set. *Phys. Rev. B* **54**, 11169–11186 (1996).
14. Kresse, G. & Hafner, J. Ab initio molecular dynamics for liquid metals. *Phys. Rev. B* **47**, 558–561 (1993).
15. Kresse, G. & Hafner, J. Ab initio molecular-dynamics simulation of the liquid-metal amorphous-semiconductor transition in germanium. *Phys. Rev. B* **49**, 14251–14269 (1994).
16. Angel, R. J., Alvaro, M. & Gonzalez-Platas, J. EosFit7c and a Fortran module (library) for equation of state calculations. *Zeitschrift für Kristallographie - Crystalline Materials* **229**, 405–419 (2014).
17. Togo, A. & Tanaka, I. First principles phonon calculations in materials science. *Scripta Materialia* **108**, 1–5 (2015).
18. Togo, A., Oba, F. & Tanaka, I. First-principles calculations of the ferroelastic transition between rutile-type and CaCl2-type SiO2 at high pressures. *Phys. Rev. B* **78**, 134106 (2008).
19. Giannozzi, P. *et al.* QUANTUM ESPRESSO: a Modular and Open-Source Software Project for Quantum Simulations of Materials. *Journal of Physics: Condensed Matter* **21**, 395502 (2009).
20. Giannozzi, P. *et al.* Advanced capabilities for materials modelling with Quantum ESPRESSO. *J. Phys.: Condens. Matter* **29**, 465901 (2017).
21. Baroni, S., de Gironcoli, S., Dal Corso, A. & Giannozzi, P. Phonons and Related Crystal Properties from Density-Functional Perturbation Theory. *Reviews of modern Physics* **73**, 515–562 (2001).
22. Allen, P. B. & Dynes, R. C. Transition temperature of strong-coupled superconductors reanalyzed. *Phys. Rev. B* **12**, 905–922 (1975).
23. Otero-de-la-Roza, A., Blanco, M. A., Pendás, A. M. & Luaña, V. Critic: a new program for the topological analysis of solid-state electron densities. *Computer Physics Communications* **180**, 157–166 (2009).
24. Otero-de-la-Roza, A., Johnson, E. R. & Luaña, V. Critic2: A program for real-space analysis of quantum chemical interactions in solids. *Computer Physics Communications* **185**, 1007–1018 (2014).
25. Yu, M. & Trinkle, D. R. Accurate and efficient algorithm for Bader charge integration. *J Chem Phys* **134**, 064111

(2011).
26. Perdew, J. P., Burke, K. & Ernzerhof, M. Generalized Gradient Approximation Made Simple. *Phys. Rev. Lett.* **77**, 3865–3868 (1996).
27. Blöchl, P. E. Projector augmented-wave method. *Physical Review B* **50**, 17953–17979 (1994).
28. Kresse, G. & Hafner, J. Ab initio molecular dynamics for liquid metals. *Phys. Rev. B* **47**, 558–561 (1993).
29. Kresse, G. & Hafner, J. Ab initio molecular-dynamics simulation of the liquid-metal-amorphous-semiconductor transition in germanium. *Phys. Rev. B* **49**, 14251–14269 (1994).
30. Kresse, G. & Furthmüller, J. Efficient iterative schemes for ab initio total-energy calculations using a plane-wave basis set. *Phys. Rev. B* **54**, 11169–11186 (1996).
31. Nosé, S. A unified formulation of the constant temperature molecular dynamics methods. *The Journal of Chemical Physics* **81**, 511–519 (1984).
32. Hoover, W. G. Canonical dynamics: Equilibrium phase-space distributions. *Phys. Rev. A* **31**, 1695–1697 (1985).
33. Kresse, G. & Hafner, J. Ab initio molecular dynamics for liquid metals. *Physical Review B* **47**, 558–561 (1993).
34. Kresse, G. & Hafner, J. Ab initio molecular-dynamics simulation of the liquid-metal-amorphous-semiconductor transition in germanium. *Physical Review B* **49**, 14251–14269 (1994).
35. Kresse, G. & Furthmüller, J. Efficient iterative schemes for ab initio total-energy calculations using a plane-wave basis set. *Physical Review B* **54**, 11169–11186 (1996).
36. Hixson, R. S. & Fritz, J. N. Shock compression of tungsten and molybdenum. *Journal of Applied Physics* **71**, 1721–1728 (1992).
37. Hill, R. The Elastic Behaviour of a Crystalline Aggregate. *Proc. Phys. Soc. A* **65**, 349 (1952).
38. Anderson, O. L. A simplified method for calculating the debye temperature from elastic constants. *Journal of Physics and Chemistry of Solids* **24**, 909–917 (1963).
39. Ravindran, P. *et al.* Density functional theory for calculation of elastic properties of orthorhombic crystals: Application to TiSi2. *Journal of Applied Physics* **84**, 4891–4904 (1998).
40. Semenok, D. V., Kruglov, I. A., Kvashnin, A. G. & Oganov, A. R. On Distribution of Superconductivity in Metal Hydrides. *Curr. Opin. Solid State Mater. Sci.* 100808 (2020) doi:10.1016/j.cossms.2020.100808.
41. Peng, F. *et al.* Hydrogen Clathrate Structures in Rare Earth Hydrides at High Pressures: Possible Route to Room-Temperature Superconductivity. *Phys. Rev. Lett.* **119**, 107001–107007 (2017).
42. Bloch, F. Zum elektrischen Widerstandsgesetz bei tiefen Temperaturen. *Z. Physik* **59**, 208–214 (1930).
43. Blatt, F. J. *Physics of Electronic Conduction in Solids*. (McGraw Hill, 1968).
44. Carbotte, J. P. Properties of boson-exchange superconductors. *Rev. Mod. Phys.* **62**, 1027–1157 (1990).
45. Larsen, A. H. *et al.* The atomic simulation environment—a Python library for working with atoms. *J. Phys.: Condens. Matter* **29**, 273002 (2017).
